# Category-Native Solomonoff Approximation: From Algorithmic Geometry to Kernels, Operators, and Induction †

**DOI:** 10.3390/e28091005

**Published:** 2026-09-08

**Authors:** Boumediene Hamzi, Marcus Hutter

**Affiliations:** 1 Department of Computing and Mathematical Sciences, California Institute of Technology, Pasadena, CA 91125, USA; 2 The Alan Turing Institute, London NW1 2DB, UK; 3 Google DeepMind, London N1C 4DJ, UK; 4 School of Computing, Australian National University, Canberra 2601, Australia

**Keywords:** Solomonoff induction, category-native approximation, position paper, Kolmogorov complexity, minimum description length, Gromov–Wasserstein distance, metric–measure spaces, model selection, kernel methods

## Abstract

Solomonoff induction mixes all computable explanations with description-length weights, but it is incomputable. This theory-and-position paper argues that practical approximation must be *category-native*: one should first declare the mathematical category in which a computable shadow will live, then use that category’s native comparison functional, complexity code, and inductive object. The proposal is not an omnibus theorem asserting that all categories are equivalent. It is a research architecture that separates comparison, representation, and prediction and makes the information lost by each projection explicit. The metric–measure branch supplies the developed realization. Compression data define an empirical Solomonoff space; Gromov–Wasserstein (GW) distance supplies relational distortion; minimum description length (MDL) controls candidate complexity; and distance-to-kernel embedding produces a positive-semidefinite predictor. For finite or countable coded classes, we prove existence and stability results, a held-out validation oracle inequality, a Kolmogorov–Solomonoff kernel unification, conditional empirical-GW consistency, and a coding-redundancy bound. Stronger learning-oracle statements remain conditional on marked/predictive selection, candidate-family adequacy, and kernel stability. Topological, Banach/Barron, graph, tree, and operator branches are presented as a constructional and testable research programme, with their maturity stated explicitly.

## 1. Introduction

Solomonoff induction is a normative ideal for sequential prediction: it averages all computable explanations with weights that decrease exponentially in description length. Its universality and incomputability are inseparable; therefore, any practical approximation chooses what structure to retain, i.e., a distance, measure, topology, norm, graph, spectrum, operator, tree, or predictive law. The choice is made before choosing an estimator, and determines which invariances are respected and which information is discarded.

This paper advances a position about that choice: computable shadows of Solomonoff induction should be *category-native*, that is, the target category should supply its comparison functional, complexity code, and downstream inductive object. The phrase is methodological, not a claim that one theorem already covers every mathematical category. Specifically, it says that Gromov–Wasserstein (GW) distortion is appropriate for relational metric–measure data, persistence distances for topological summaries, norm or variation discrepancies for Banach/Barron models, graph or diffusion distances for graph structure, and operator distances for spectral models. Forcing all of these objects through a single Hilbertian or metric functional would erase the very structure that the approximation is meant to preserve.

The metric–measure (mm) branch is this paper’s theorem-bearing realization. Observations are encoded as an empirical mm-space in which distances are compressor-based surrogates for algorithmic distance and masses are empirical or Occam-weighted. Candidate mm-spaces are compared to this object by GW distance and selected by

$$\hat{\theta }\in \underset{\theta }{arg min}\left\{d_{\mathrm{GW},p}^{p}\left({\hat{S}}_{n},M_{\theta }\right)+\lambda \;\mathrm{Code}\left(\theta \right)\right\}.$$

For supervised tasks, the objective must also contain a marked or held-out predictive term. This distinction is substantive: unmarked GW is invariant to relabeling, and can preserve relational geometry while destroying prediction. The resulting design rule is that the native comparison must include the marks or predictive loss relevant to the task, and that the candidate family must retain the structure on which those marks depend.

**Empirical scope.** The numerical section evaluates the finite surrogate through mechanism checks rather than a benchmark claim. Controlled string corpora test recovery of planted low-complexity block structure, rejection of structureless noise, the need to penalize fitted flexibility against memorizing candidates, and held-out relational selection. Separate finite studies probe spectral calibration under exact and approximate orthogonality, GW-to-kernel stability, the pairing dependence of metric repair, cross-compressor stability, concentration under Occam-reweighted mass, and the marked-coupling mechanism. Downstream prediction is then stress-tested in two harder settings. On a cellular-automaton classification task, geometry-only selection favors a one-block quotient and produces chance-level balanced accuracy; a nested stratified selector that is allowed to retain the raw compression geometry avoids this collapse but does not improve on the raw compression-kernel baseline. On a balanced subset of the UCI SMS Spam Collection, fused marked selection does not rescue average-linkage block quotients whose partitions are label-mixed, whereas the uncoarsened compression geometry retains strong predictive signal. Even a post-hoc oracle over the fitted block kernels remains at chance. Together these studies distinguish two requirements: task alignment can protect against an unsuitable geometry-only objective, but neither marks nor validation can recover predictive information already discarded by an inadequate candidate family.

This paper makes five contributions at two deliberately separated levels:

1. It formulates category-native Solomonoff approximation as a common *problem architecture*: choose a target category, a native distortion, a prefix-decodable model code, and a native inductive object. This is the paper’s positional and organizational contribution, not a cross-category equivalence theorem.
2. It supplies a maturity map for topological, Hilbert/Banach, graph, tree, operator, predictive, and metric–measure branches. The map makes the proposed objects and the missing stability or prediction results explicit, so the vision is testable rather than merely rhetorical.
3. It develops the metric–measure branch: the empirical Solomonoff mm-space, the GW + MDL selector, and a marked extension that aligns geometry and task loss through the same coupling.
4. It proves the finite/countable technical core: existence and perturbation results, a held-out validation oracle inequality for coded candidates, a finite transfer statement, downstream kernel/operator recovery, conditional empirical-GW consistency, and a coding redundancy bound for mixtures over coded mm-models.
5. It supplies numerical stress tests of structural recovery, negative controls, spectral and kernel stability, compressor sensitivity, marked coupling, and downstream prediction. The cellular-automaton and real SMS studies show that unmarked selection can collapse and that task marks cannot repair a quotient family that has already discarded predictive structure. A validation safeguard that can retain the raw geometry prevents collapse. These results correct an unconditional “geometry first” reading without abandoning the wider claim that different representational categories require different, task-aligned notions of fit.

The logical status of the two levels is summarized in Table 1. A position can be substantive without necessarily being a theorem: it should organize previously separate constructions, state design commitments, expose failure conditions, and generate a programme of discriminating results. The metric–measure theorems then demonstrate that one branch can be carried from the position to a computable selector and conditional learning guarantees. In particular, the conceptual GW + MDL definition does not imply the later learning oracle inequality. That inequality additionally assumes a coded candidate class, independent validation or an equivalent generalization device, marked/predictive alignment, and stability of the induced kernel learner.

**What kind of paper this is.** The claims have three statuses: *(A) Position:* Category-native approximation is an organizing proposal, defended by the separation of native comparison, representation, and induction and by the distinct questions it generates; it is not presented as a derived cross-category theorem. *(B) Proved core:* The metric–measure branch contains the finite/countable results stated with their hypotheses. *(C) Research programme:* The topological, Banach/Barron, graph, tree, and broad operator branches specify constructions and proof obligations that remain open. These levels are complementary, not interchangeable.

**How to read the paper.** Readers interested primarily in the technical case may read Section 5, Section 7, Section 11, Section 15 and Section 17 and the predictive-collapse and real-data marked-selection analyses in Section 14. Readers evaluating the positional contribution should additionally follow the three category-by-category sweeps, covering native comparisons in Section 8, representation and kernelization in Section 11, and induction in Section 11.5. The maturity map in Section 2 states which claims are proved, constructional, or open.

Parts I–VI of the *Bridging Algorithmic Information Theory and Machine Learning* series [1,2,3,4,5,6] studied compression-based kernels, spectra, and Gaussian/Hilbert constructions. The present paper supplies the metric–measure selection layer that precedes those constructions and shows precisely when the earlier kernels are recovered. A broader kernel-first synthesis of Parts I–XXI, including the category-native extensions, their comparison–representation–induction atlas, and a registry separating proved, conditional, empirical, and programme-level claims, is developed in the accompanying research monograph preprint [7]. The present paper remains responsible for the claims made here; the monograph supplies wider programme-level context rather than replacing the metric–measure arguments below. Neither work claims that a kernel, Gaussian process, or mm-space equals the universal semimeasure. Table 2 collects the acronyms used in the manuscript, and Table 3 fixes the notation that separates ideal, empirical, and selected objects.

The manuscript keeps two threads visible: Section 2 states the category-native position and the commitments by which it should be judged, while the remainder develops metric–measure approximation, kernel recovery, error decomposition, and finite/countable guarantees as the most mature realization. This asymmetry is intentional; a position paper can propose the architecture of a field while also being explicit about which branch has reached theorem level. Table 4 records that maturity assessment branch by branch.

## 2. Category-Native Solomonoff Approximation: The Position

The central position is that no category-independent scalar discrepancy can preserve every computable shadow of Solomonoff induction. A topology forgets metric scale; a metric without mass forgets posterior weight; a spectrum can forget eigenfunctions; a kernel commits to a Hilbertian representation; and an unmarked geometry forgets labels. These are not interchangeable defects. They are category-specific projection choices. The right question is not “which single distance approximates Solomonoff induction?” but “which invariant should be preserved for the representation and task at hand?”

Let S denote a Solomonoff-side source: a prior prefix tree, a posterior space of explanations, or a computable empirical shadow. For a target category C, the organizing problem is

(1) $$\Delta_{C}\left(S\right)=\underset{A\in C}{\inf}\left\{D_{C}\left(S,F_{C}\left(A\right)\right)+\lambda \mathrm{Code}\left(A\right)+\eta \;{\mathrm{PredLoss}}_{C}\left(A\right)\right\}.$$

Here, $F_{C}$ exposes the part of a candidate that can be compared with the source, $D_{C}$ respects the invariances native to C, Code(A) prices the computable candidate, and the predictive term is included whenever structure alone is insufficient for the task. The commonality lies in the architecture of the objective; its first and last terms are deliberately category- and task-dependent.

“Comparison functional” here is intentionally broader than “metric minimized at zero”. A multiplicative distortion with optimum (1) may be logged and a similarity maximized at (1) may be converted into a loss, but its orientation, normalization, and null equivalence must be declared. Persistence and spectral summaries may also be invariant without being faithful to the underlying object. Thus, (1) is a typed optimization schema, not a universal metric axiom; forcing every row to satisfy one metric definition would contradict the category-native premise rather than formalizing it.

The accompanying research-monograph preprint develops this architecture across Parts I–XXI and gives a more extensive atlas of metric, metric–measure, Hilbert/RKHS, Banach/Barron, operator, tree/graph, topological, and predictive law targets [7]. The present paper supplies the detailed metric–measure realization within that wider programme.

Three commitments make this more than a naming exercise. First, *native invariance*: the comparison must respect the equivalences of the target representation rather than importing an arbitrary Euclidean proxy. Second, *compositional accountability*: comparison, representation, and induction must be stated separately, so that a good structural match is not silently converted into a predictive guarantee. Third, *empirical corrigibility*: the cellular-automaton collapse and the real-data marked-selection results must be allowed to revise the objective. In supervised settings, permutation-invariant unmarked fit is inadequate; marks, validation loss, or another task-aware term must enter, and the candidate family must retain task-relevant structure.

The programme would fail as a scientific position if category-specific choices could never change model rankings, stability statements, or predictions. It instead generates concrete questions: when does a persistence comparison retain information that GW discards? when does a Barron variation code outperform an RKHS code? when does a graph diffusion preserve task-relevant structure better than its shortest-path mm-space shadow? and when does a direct predictive mixture dominate every single structured projection? This paper answers the corresponding metric–measure question farthest. The other rows remain invitations to construct and falsify branch-specific theories, not decorative claims of completed unification.

## 3. Metric–Measure Realization

This section identifies the Solomonoff-side source objects represented as metric–measure spaces. GW is not asked to discover the space where induction “really lives.” A source mm-space or finite shadow is fixed first; GW then measures the distortion incurred by representing it inside a restricted candidate class.

### 3.1. Variational, Not Ontological

The operative distinction is:

$$\begin{array}{c} \mathrm{GW}\;\mathrm{is}\;\mathrm{variational},\;\mathrm{not}\;\mathrm{ontological}. \end{array}$$

For a source mm-space S and a restricted class G, define

(2) $$\Delta_{G}\left(S\right)=\underset{M\in G}{\inf}\left\{d_{\mathrm{GW},p}^{p}\left(S,M\right)+\lambda \mathrm{Code}\left(M\right)\right\}.$$

The code or declared MDL score prevents a vacuous preference for an increasingly flexible copy of the source.

This restriction is essential. If G contains every mm-space and S itself is admissible at no complexity cost, the GW term can be zero. The research content lies in computability, code length, finite rank, ultrametric, graph-induced, manifold, spectral, or task-compatibility restrictions on G.

### 3.2. Three Solomonoff-Side Source Objects

There are three source objects which should not be conflated.

**Prior-side output tree.** Solomonoff induction is classically defined on finite and infinite binary strings. Let

$${\left\{0,1\right\}}^{\leq \infty }={\left\{0,1\right\}}^{*}\cup {\left\{0,1\right\}}^{N}$$

and equip it with the prefix ultrametric

$$d_{\mathrm{tree}}\left(\omega ,\omega^{\prime }\right)=\left\{\begin{array}{cc} 0, & \omega =\omega^{\prime },\\ 2^{-|\mathrm{lcp}(\omega ,\omega^{\prime })|}, & \omega \neq \omega^{\prime }. \end{array}\right.$$

The monotone universal semimeasure is normalized at the empty string, with any remaining initial deficit assigned to that node. Assigning each prefix mass deficit to its finite-output node then gives a probability measure on this compact extension. This gives the prior-side mm-space

$$S_{\mathrm{tree}}=\left({\left\{0,1\right\}}^{\leq \infty },d_{\mathrm{tree}},M\right).$$

This is canonical and useful for sequence-prediction questions, but captures output-prefix geometry rather than the posterior geometry of explanations after data have been observed.

**Posterior explanation geometry.** For a dataset or history *D*, let $H_{U}$ be a computable hypothesis, program, grammar, dictionary, compressor state, or semimeasure class. In the measure–mixture presentation, write

$$\pi_{D}\left(h\right)=\frac{2^{-\ell (h)}\nu_{h}\left(D\right)}{\sum_{g}2^{-\ell (g)}\nu_{g}\left(D\right)}.$$

Assume the denominator is finite and positive. In the monotone program presentation, consistency replaces the likelihood. Choose a bounded measurable pseudometric $d_{\exp}$, quotient by its zero-distance relation ∼, push the posterior mass forward to that quotient, and take its metric completion (with the pushed-forward measure supported on the original quotient). Define

(3) $$S_{D}=\left(H_{U}/\;∼,\;d_{\exp},\;\pi_{D}\right).$$

The predictive pseudometric specified below provides one such construction. Algorithmic or hybrid choices are admissible only after their metric and quotient properties have been established. This is the native object closest to Solomonoff induction as a data-conditioned learning procedure: its points are explanations, not observed data points.

**Empirical data-side shadow.** The computable object used in Section 5 is

$${\hat{S}}_{n}=\left(X_{n},{\hat{d}}_{\mathrm{Sol}},{\hat{\mu }}_{n}\right).$$

This is a behavioral shadow of $S_{D}$: observations inherit a finite relational geometry from compressor surrogates, finite program dictionaries, or other computable approximations to algorithmic distance. This is the object we can compare by GW in practice.

Therefore, the intended conceptual order is

$$\begin{array}{c} \begin{array}{c} S_{\mathrm{tree}}\;\mathrm{or}\;S_{D}\;⟶\;{\hat{S}}_{n}\;⟶\;\mathrm{restricted}\;\mathrm{mm}\text{-}\mathrm{space}\\ ⟶\;\mathrm{induced}\;\mathrm{kernel}/\mathrm{operator}\;⟶\;\mathrm{predictor}. \end{array} \end{array}$$

### 3.3. Triage of Approximation Problems

The following table records which approximation cells are mathematical content and which are calibration or sanity checks.

The paper’s main objective in Equation (5) should be read as a restricted approximation principle: the candidate family G is doing mathematical work; without that restriction, the problem is vacuous, while with it GW can measure how expensive it is to represent Solomonoff relational structure in the chosen geometry class. Table 5 triages the restricted problems by data regime and candidate class.

## 4. Materials and Methods: Mathematical and Computational Framework

### 4.1. Kolmogorov Complexity and Algorithmic Distances

Fix a universal-prefix Turing machine *U*. We use standard notation from Kolmogorov complexity and universal induction [8,9,10,11,12,13,14,15,16,17]; see [18] for a treatment of universal induction in this journal, [19] for a philosophical critique, and [20] for general information-theoretic background. The prefix Kolmogorov complexity of a finite object *x* is

K(x)=min{|p|:U(p)=x}.

The conditional complexity is

K(x∣y)=min{|p|:U(p,y)=x}.

The algorithmic mutual information is

$$I_{\mathrm{alg}}\left(x:y\right)=K\left(x\right)+K\left(y\right)-K\left(x,y\right).$$

The normalized information distance is an ideal algorithmic dissimilarity

$$\mathrm{NID}\left(x,y\right)=\frac{\max\{K(x|y),K(y|x)\}}{\max\{K(x),K(y)\}}$$

whose metric properties hold only up to the usual precision terms. Exact metric–measure theorems below require a specified genuine metric (or a pseudometric quotient and completion); an unrepaired dissimilarity is used only in the finite network objective. For notation, we write $d_{\mathrm{Sol}}$ for an ideal algorithmic distance of this kind and ${\hat{d}}_{\mathrm{Sol}}$ for its computable compressor surrogate; hatless occurrences always refer to the ideal object. Since *K* is incomputable, applications use computable surrogates such as normalized compression distance [21,22,23,24]

$${\mathrm{NCD}}_{C}\left(x,y\right)=\frac{C(x\circ y)-\min\{C(x),C(y)\}}{\max\{C(x),C(y)\}},$$

where *C* is a lossless compressor and x∘y denotes a self-delimiting concatenation of the two strings, following the compression distance viewpoint of [22,23].

For the purposes of this paper, ${\hat{d}}_{\mathrm{Sol}}$ denotes any computable algorithmic dissimilarity intended to approximate an ideal Solomonoff or Kolmogorov distance.

**Remark 1.**
  *The framework does not require a single canonical empirical Solomonoff distance. Different compressors, program dictionaries, or KC-kernel surrogates give different empirical views of algorithmic structure. This is a feature, not a bug: cross-compressor stability becomes an empirical test of whether a discovered geometry is genuinely algorithmic rather than compressor-specific.*

### 4.2. Metric–Measure Spaces

The metric–measure viewpoint, its topology of isomorphism classes, and its convergence theory originated with Gromov and Sturm [25,26]; see also [27].

**Definition 1** (Metric–measure space)**.**  *A metric–measure space, or mm-space, is a triple*

$$M=(X,d_{X},\mu_{X}),$$

*where $\left(X,d_{X}\right)$ is a complete separable metric space and $\mu_{X}$ is a Borel probability measure on X. If X is finite, we identify $\mu_{X}$ with a probability vector. Isomorphism statements concern the supports of the measures; zero-mass points do not affect GW.*

Two mm-spaces are identified if there is a measure-preserving isometry between their supports. This is the correct equivalence relation for relational geometry; labels and coordinates are irrelevant.

### 4.3. Gromov–Wasserstein Distance

Let $M=(X,d_{X},\mu_{X})$ and $N=(Y,d_{Y},\mu_{Y})$. A coupling between $\mu_{X}$ and $\mu_{Y}$ is a probability measure $\pi \in \Pi (\mu_{X},\mu_{Y})$ on X×Y with marginals $\mu_{X}$ and $\mu_{Y}$. GW distances were introduced by Mémoli [28], and in the form used here, by Sturm [29]; computational aspects and entropic solvers are surveyed in [30,31,32,33,34], and structured-data variants include fused GW [35], graph matching and node embedding [36], network invariants [37], generative alignment across incomparable spaces [38], low-rank solvers [39], Gaussian restrictions [40,41], Procrustes metrics on covariance operators [42], generalized spectral clustering via GW learning [43], and GW-based structure learning of latent graphs and dictionaries [44,45,46].

**Definition 2** (Gromov–Wasserstein distance)**.**  *For p≥1, the p-Gromov–Wasserstein distance is*

$$d_{\mathrm{GW},p}^{p}\left(M,N\right)=\frac{1}{2}\underset{\pi \in \Pi (\mu_{X},\mu_{Y})}{\inf}\int {\left|d_{X}\left(x,x^{\prime }\right)-d_{Y}\left(y,y^{\prime }\right)\right|}^{p}\;d\pi \left(x,y\right)\;d\pi \left(x^{\prime },y^{\prime }\right).$$

For finite spaces $X={\left\{x_{i}\right\}}_{i=1}^{n}$, $Y={\left\{y_{a}\right\}}_{a=1}^{m}$, this becomes

$$d_{\mathrm{GW},p}^{p}\left(M,N\right)=\frac{1}{2}\min_{\Pi \in U(\alpha ,\beta )}\sum_{i,i^{\prime },a,a^{\prime }}{\left|D_{ii^{\prime }}^{X}-D_{aa^{\prime }}^{Y}\right|}^{p}\Pi_{ia}\Pi_{i^{\prime }a^{\prime }},$$

where $D^{X},D^{Y}$ are distance matrices, $\alpha_{i}=\mu_{X}\left(\left\{x_{i}\right\}\right)$ and $\beta_{a}=\mu_{Y}\left(\left\{y_{a}\right\}\right)$ are the probability vectors of the two measures, and U(α,β) is the transportation polytope with those marginals.

### 4.4. D2KE and Geometry-Induced Kernels

Given a metric–measure space M=(Z,d,μ), one can construct positive semidefinite kernels from *d* in several ways. If $d^{2}$ is conditionally negative definite, then

$$k_{\gamma }\left(z,z^{\prime }\right)=\exp\left(-\gamma d{\left(z,z^{\prime }\right)}^{2}\right)$$

is positive semidefinite by Schoenberg’s theorem [47,48]. More generally, D2KE (distance-to-kernel-and-embedding, due to Wu et al. [49]) constructs a positive semidefinite kernel by using distances to landmarks, in the spirit of the KC-kernel and Mercerization constructions of the earlier BAITML papers [1,2,3]; general RKHS background is standard [50,51,52]:

(4) $$k_{D2\mathrm{KE},\gamma }^{M}\left(z,z^{\prime }\right)=\int_{Z}\exp\left(-\gamma d\left(z,\omega \right)\right)\exp\left(-\gamma d\left(z^{\prime },\omega \right)\right)\;d\mu \left(\omega \right).$$

This kernel is positive semidefinite because it is an inner product of feature maps

$$\Phi_{z}\left(\omega \right)=\exp\left(-\gamma d\left(z,\omega \right)\right)\;\mathrm{in}\;L^{2}\left(Z,\mu \right).$$

In the present paper, (4) is the default way of passing from learned geometry to RKHS/GP machinery. This step guarantees a positive-semidefinite representation; it does *not* guarantee that the induced kernel metric reproduces the input distance. For a finite weighted sample, a practical audit should report both a conditionally negative-definite defect obtained from the negative spectrum of the centered matrix $-\frac{1}{2}JD^{\circ 2}J$ and the mass-weighted distortion between *D* and the D2KE-induced kernel distance. A large defect means that Hilbertization can change neighborhoods or rankings even though the resulting kernel remains valid for prediction. That loss belongs to $\delta_{\ker}$ in the error budget, and is precisely why the category-native comparison is performed prior to D2KE.

## 5. The Empirical Solomonoff Metric–Measure Space

### 5.1. Definition

**Definition 3** (Empirical Solomonoff mm-space)**.**  *Let $X_{n}=\left\{x_{1},\ldots ,x_{n}\right\}$ be a dataset and let ${\hat{d}}_{\mathrm{Sol}}:X_{n}\times X_{n}\to R_{+}$ be a symmetric computable algorithmic dissimilarity with zero diagonal. Let ${\hat{\mu }}_{n}$ be either the uniform empirical measure or an Occam-reweighted empirical measure. The empirical Solomonoff mm-space is*

$${\hat{S}}_{n}=\left(X_{n},{\hat{d}}_{\mathrm{Sol}},{\hat{\mu }}_{n}\right).$$

The distance ${\hat{d}}_{\mathrm{Sol}}$ need not be a perfect metric. In practice, NCD-like quantities may have small violations of the triangle inequality or even mild asymmetries. There are three standard repairs:

$${\hat{d}}_{\mathrm{sym}}\left(x,y\right)=\frac{1}{2}\left(\hat{d}\left(x,y\right)+\hat{d}\left(y,x\right)\right),$$

$${\hat{d}}_{\mathrm{met}}\left(x,y\right)=\mathrm{shortest}\text{-}\mathrm{path}\;\mathrm{metric}\;\mathrm{generated}\;\mathrm{by}\;{\hat{d}}_{\mathrm{sym}},$$

or direct use of a distortion functional for dissimilarity spaces rather than metric spaces. The clean theory below assumes a bounded metric, but the algorithmic formulation can be applied to repaired empirical distances as well.

### 5.2. Choice of Empirical Mass

The simplest mass is uniform:

$${\hat{\mu }}_{n}=\frac{1}{n}\sum_{i=1}^{n}\delta_{x_{i}}.$$

For a more Solomonoff-like empirical object, use an Occam-reweighted measure. Here, $\hat{K}\left(x_{i}\right)$ denotes a computable code length for $x_{i}$, for example a compressed bit length plus any delimiter or model-header convention used by the compressor. In exact algebra, one may write

$${\hat{\mu }}_{n}^{\mathrm{Occam}}\left(x_{i}\right)\propto 2^{-\hat{K}\left(x_{i}\right)}.$$

For numerical work, this expression should be evaluated in log-space and with a declared effective code ${\hat{K}}^{†}$,

$$K_{\min}^{†}:=\min_{1\leq j\leq n}{\hat{K}}^{†}\left(x_{j}\right),\;{\hat{\mu }}_{n,\tau }^{\mathrm{Occam}}\left(x_{i}\right)=\frac{\exp\{-\tau \left({\hat{K}}^{†}\left(x_{i}\right)-K_{\min}^{†}\right)\}}{\sum_{j=1}^{n}\exp\left\{-\tau \left({\hat{K}}^{†}\left(x_{j}\right)-K_{\min}^{†}\right)\right\}},\;\tau \geq 0.$$

For objects of comparable lengths, one may take ${\hat{K}}^{†}=\hat{K}$. When raw lengths vary materially, raw Solomonoff weighting intentionally favors physically shorter objects; if that is not the intended estimand, one must instead declare a conditional code ${\hat{K}}^{†}\left(x\right)=\hat{K}\left(x||x|\right)$, stratify by length, or use another explicitly justified nuisance-length adjustment. Dividing by |x| gives a compression rate, but does not automatically provide a Kraft-valid object code and should not be called Solomonoff mass without an additional coding argument. Subtracting $K_{\min}^{†}$ does not change the normalized weights but prevents floating-point underflow. The temperature τ controls the strength of the Occam bias (throughout, log denotes the natural logarithm and code lengths are in bits): τ=ln2 recovers the bit-length convention, smaller values give a softer Solomonoff tilt, and τ=0 gives the uniform empirical measure. To make clear when the Occam measure has collapsed toward a small number of atoms, in numerical experiments one should report ${\hat{K}}^{†}$, τ, any clipping or flooring of weights, and the effective sample size (which is an entropic quantity; $\mathrm{ESS}\left(\hat{\mu }\right)=\exp\left(H_{2}\left(\hat{\mu }\right)\right)$, the exponential of the Rényi-2 entropy, so the collapse diagnostic below is a Rényi-entropy floor):

$$\mathrm{ESS}\left(\hat{\mu }\right)=\frac{1}{\sum_{i}{\hat{\mu }}_{i}^{2}}.$$

The uniform version treats the dataset as observed samples, while the Occam version treats shorter descriptions as more central in the empirical geometry. The correct choice depends on whether the goal is descriptive modeling of the observed distribution or approximation of a universal prior.

### 5.3. Marked Empirical Solomonoff Spaces

For supervised learning, the empirical object should include labels. Let $y_{i}\in Y$. A marked empirical Solomonoff space is

$${\hat{S}}_{n}^{\;\mathrm{mark}}=\left(X_{n},{\hat{d}}_{\mathrm{Sol}},{\hat{\mu }}_{n},\ell_{n}\right),$$

where $\ell_{n}\left(x_{i}\right)=y_{i}$. The labels are not inserted by changing the input metric alone; rather, they enter through a marked GW objective. This avoids confusing algorithmic similarity of inputs with predictive relevance.

## 6. Behavioral Transfer from Explanation Geometry to Data Geometry

The empirical space ${\hat{S}}_{n}$ is useful only if it preserves enough of the posterior explanation geometry $S_{D}$. This section makes that bridge explicit. It is a load-bearing assumption for interpreting data-side GW as evidence about explanation-side GW.

Let

$$B_{D}:H_{U}/\;∼\;⟶\;B_{n}$$

be the behavior map sending an explanation to its finite behavior on the observed sample, histories, labels, or query set. In the ideal case,

$${\hat{\mu }}_{n}\approx {\left(B_{D}\right)}_{\#}\pi_{D},$$

and ${\hat{d}}_{\mathrm{Sol}}\left(B_{D}h,B_{D}g\right)$ approximates the explanation distance $d_{\exp}\left(h,g\right)$ on the posterior mass that matters.

**Definition 4** (Behavioral faithfulness)**.**  *The behavior map is (c,C,ε,δ)-faithful on posterior mass, with normalized constants 0<c≤1≤C<∞, if there exists a measurable set $E\subseteq H_{U}/\;∼$ with $\pi_{D}\left(E\right)\geq 1-\delta$ such that for all h,g∈E,*

$$c\;d_{\exp}\left(h,g\right)-\varepsilon \leq {\hat{d}}_{\mathrm{Sol}}\left(B_{D}h,B_{D}g\right)\leq C\;d_{\exp}\left(h,g\right)+\varepsilon .$$

*There is no loss in this normalization; any valid positive lower constant can be replaced by min(c,1) and any valid upper constant by max(C,1), only weakening the two inequalities.*

**Lemma 1** (Behavioral transfer, template)**.**  *Assume that $B_{D}$ is (c,C,ε,δ)-faithful on posterior mass with 0<c≤1≤C and that ${\hat{S}}_{n}$ approximates the push-forward ${\left(B_{D}\right)}_{\#}S_{D}$ with sampling/compressor error $\eta_{n}$ in bounded GW distance. Then, simultaneously for every R bounding the diameters of $S_{D}$, its image ${\left(B_{D}\right)}_{\#}S_{D}$, ${\hat{S}}_{n}$, and the candidate mm-space M,*

$$\left|d_{\mathrm{GW},p}\left(S_{D},M\right)-d_{\mathrm{GW},p}\left({\hat{S}}_{n},M\right)\right|\leq \Phi_{p}\left(c,C,\varepsilon ,\delta ,\eta_{n},R\right),$$

*with the explicit modulus*

$$\Phi_{p}\;\leq \;2^{-1/p}\varepsilon +\left(\max(C-1,\;1-c)\right)R+R\;{\left(2\delta \right)}^{1/p}+\eta_{n}.$$

*Thus, in particular, $\Phi_{p}\to 0$ as c,C→1 and $\varepsilon ,\delta ,\eta_{n}\to 0$. Evaluating the modulus requires the faithfulness constants; when they are unknown, the bound is uninstantiable rather than false, which is the honest reading of Remark 2 below.*

**Proof.**  By the triangle inequality for $d_{\mathrm{GW},p}$, $\left|d_{\mathrm{GW},p}\left(S_{D},M\right)-d_{\mathrm{GW},p}\left({\hat{S}}_{n},M\right)\right|\leq d_{\mathrm{GW},p}\left(S_{D},{\hat{S}}_{n}\right)\leq d_{\mathrm{GW},p}\left(S_{D},{\left(B_{D}\right)}_{\#}S_{D}\right)+\eta_{n}$, the second step by the hypothesis that ${\hat{S}}_{n}$ approximates the push-forward within $\eta_{n}$. Therefore, it suffices to bound the first term by the remaining three summands. Couple $S_{D}$ with its push-forward by the graph coupling $\pi ={\left(\mathrm{id}\times B_{D}\right)}_{\#}\pi_{D}$, which is admissible because its marginals are $\pi_{D}$ and ${\left(B_{D}\right)}_{\#}\pi_{D}$. Then, by Definition 2,

$$d_{\mathrm{GW},p}^{p}\left(S_{D},{\left(B_{D}\right)}_{\#}S_{D}\right)\leq \frac{1}{2}\;\int \int \;{\left|d_{\exp}\left(x,x^{\prime }\right)-{\hat{d}}_{\mathrm{Sol}}\left(B_{D}x,B_{D}x^{\prime }\right)\right|}^{p}\;d\pi_{D}\left(x\right)\;d\pi_{D}\left(x^{\prime }\right).$$

Let *E* be the faithful set with $\pi_{D}\left(E\right)\geq 1-\delta$. Split the double integral. On the bad event $\left\{x\notin E\right\}\cup \left\{x^{\prime }\notin E\right\}$, of $\pi_{D}⊗\pi_{D}$-mass at most $1-{\left(1-\delta \right)}^{2}\leq 2\delta$, both dissimilarities lie in [0,R] (here, the diameter hypothesis on $S_{D}$ and on the image is used), so the integrand is at most $R^{p}$. On the good event, (c,C,ε)-faithfulness gives $\left|{\hat{d}}_{\mathrm{Sol}}\left(B_{D}x,B_{D}x^{\prime }\right)-d_{\exp}\left(x,x^{\prime }\right)\right|\leq \max\left(C-1,\;1-c\right)\;d_{\exp}\left(x,x^{\prime }\right)+\varepsilon \leq \max\left(C-1,\;1-c\right)\;R+\varepsilon$. Combining,

$$d_{\mathrm{GW},p}^{p}\leq \frac{1}{2}{\left(\max(C-1,1-c)R+\varepsilon \right)}^{p}+\frac{1}{2}\;\left(2\delta \right)\;R^{p}.$$

Taking *p*th roots and using ${\left(a+b\right)}^{1/p}\leq a^{1/p}+b^{1/p}$ for a,b≥0,

$$d_{\mathrm{GW},p}\leq 2^{-1/p}\left(\max(C-1,1-c)R+\varepsilon \right)+R\;\delta^{1/p}\leq 2^{-1/p}\varepsilon +\max\left(C-1,1-c\right)\;R+R\;{\left(2\delta \right)}^{1/p},$$

where the last step drops the factor $2^{-1/p}\leq 1$ on the bi-Lipschitz term and enlarges $\delta^{1/p}\leq {\left(2\delta \right)}^{1/p}$; both replacements only weaken the bound. Adding $\eta_{n}$ completes the proof.    □

**Proposition 1** (Finite faithful-transfer instance)**.**  *Let $E=\{h_{1},\ldots ,h_{m}\}$ be a finite posterior explanation support with masses $\pi_{i}$ and explanation metric $d_{\exp}$. Suppose that the behavior map $B_{D}$ is injective on E and write*

$$S_{E}=\left(E,d_{\exp},\pi \right),\;{\hat{S}}_{E}=\left(B_{D}E,{\hat{d}}_{\mathrm{Sol}},{\left(B_{D}\right)}_{\#}\pi \right).$$

*If the compressor or program dictionary distance is uniformly faithful on this support,*

$$\max_{i,j}\left|{\hat{d}}_{\mathrm{Sol}}\left(B_{D}h_{i},B_{D}h_{j}\right)-d_{\exp}\left(h_{i},h_{j}\right)\right|\leq \varepsilon ;$$

*then, for every candidate metric–measure space M,*

$$\left|d_{\mathrm{GW},p}\left(S_{E},M\right)-d_{\mathrm{GW},p}\left({\hat{S}}_{E},M\right)\right|\leq 2^{-1/p}\varepsilon .$$

*If a second empirical finite shadow, denoted ${\tilde{S}}_{n}$, is additionally within bounded GW distance $\eta_{n}$ of ${\hat{S}}_{E}$, the right-hand side becomes $2^{-1/p}\varepsilon +\eta_{n}$.*

**Proof.**  Using the bijection $h_{i}\mapsto B_{D}h_{i}$ to identify the two finite supports and keep the same masses, for any coupling between $S_{E}$ and M, we can use the corresponding coupling between ${\hat{S}}_{E}$ and M. The two GW integrands differ only by the source metric perturbation, which is bounded by ε. Taking the $L^{p}$ norm and remembering the 1/2 convention in the definition of $d_{\mathrm{GW},p}$ gives the factor $2^{-1/p}$. Repeating the argument in the reverse direction gives the absolute value. The final statement is the triangle inequality.    □

**Remark 2** (Why a transfer condition is necessary)**.**  *Without a co-Lipschitz or injectivity on posterior support condition, the behavior map can collapse the explanation geometry. Two programs can agree on $X_{n}$ while remaining far apart algorithmically; in that case, ${\hat{S}}_{n}$ may be close to a candidate even though $S_{D}$ is not. The marked objective, cross-compressor checks, and out-of-sample behavior probes are practical attempts to detect this collapse.*

## 7. Solomonoff–Gromov Model Selection

### 7.1. Candidate Geometries

A model class is now a family of mm-spaces

$$G=\{M_{\theta }=\left(Z_{\theta },d_{\theta },\mu_{\theta }\right):\theta \in \Theta \}.$$

Examples include:

1. Finite latent metric spaces with *m* points.
2. Weighted graphs with shortest-path or diffusion distances.
3. Ultrametric trees, especially prefix trees.
4. Riemannian manifolds with volume measures.
5. Latent Euclidean spaces with learned embeddings.
6. Covariance-operator geometries induced by Gaussian processes.
7. Program dictionaries with prefix or edit distances.
8. Barycenters of several empirical Solomonoff spaces.

The parameter θ may encode a finite matrix, neural embedding, graph, tree, manifold family, compressor family, kernel hyperparameter, or operator truncation. Two complexity regimes must be distinguished. For a countable family, Code(θ) denotes a genuine Kraft-summable prefix code length. For continuously fitted parameters, the practical objective may instead use a BIC/MDL complexity score such as an index code plus $\frac{1}{2}q\left(\theta \right)\log_{2}n$, where q(θ) counts fitted real parameters. The latter is an asymptotic complexity penalty; unless parameter values are explicitly quantized and encoded, it is *not* a literal two-part prefix code over all fitted configurations. The distinction matters, as the Kraft-based mixture and countable-class theorems below apply only to the first regime or to an explicitly coded finite-precision discretization.

### 7.2. Unsupervised Objective

**Definition 5** (Solomonoff–Gromov objective)**.**  *Given ${\hat{S}}_{n}$ and a candidate family G, the unsupervised Solomonoff–Gromov objective is*

(5) $$J_{n}\left(\theta \right)=d_{\mathrm{GW},p}^{p}\left({\hat{S}}_{n},M_{\theta }\right)+\lambda \;\mathrm{Code}\left(\theta \right).$$

*Any minimizer*

$$\hat{\theta }\in \underset{\theta \in \Theta }{arg min}J_{n}\left(\theta \right)$$

*is called a Solomonoff–Gromov model.*

**Example 1** (A fully worked three-model selection)**.**  *Take three observations with uniform mass and empirical distance matrix*

$$D^{S}=\left(\begin{array}{ccc} 0 & 1 & 1\\ 1 & 0 & 2\\ 1 & 2 & 0 \end{array}\right).$$

*Compare three prefix-coded candidates: A reproduces $D^{S}$ and has a code length of 9 bits, B is the three-point equilateral space*

$$D^{B}=\left(\begin{array}{ccc} 0 & 1 & 1\\ 1 & 0 & 1\\ 1 & 1 & 0 \end{array}\right)$$

*with code length of *3* bits, and C is a one-point space with code length of *1* bit. The code lengths are Kraft-valid because $2^{-9}+2^{-3}+2^{-1}<1$.*

*For p=2, the identity/permutation coupling assigns mass 1/3 to each matched pair. Candidate A has zero distortion. For B, only the ordered source pairs (2,3) and (3,2) each differ from the candidate by one, so*

$$d_{\mathrm{GW},2}^{2}\left(S,A\right)=0,\;d_{\mathrm{GW},2}^{2}\left(S,B\right)=\frac{1}{2}\frac{2}{9}=\frac{1}{9}.$$

*For the one-point candidate, every target distance is zero; the six off-diagonal source entries contribute four values of $1^{2}$ and two of $2^{2}$, hence*

$$d_{\mathrm{GW},2}^{2}\left(S,C\right)=\frac{1}{2}\frac{4+2\cdot 4}{9}=\frac{2}{3}.$$

*For completeness, the value for B is globally optimal. For a coupling π between the two uniform three-point measures, put $s_{ij}=\sum_{a}\pi_{ia}\pi_{ja}$. Expanding the half-normalized objective against an equilateral target of distance c gives*

$$C\left(\pi \right)-C\left(\pi_{\mathrm{perm}}\right)=c\sum_{i\neq j}D_{ij}^{S}s_{ij}\geq 0.$$

*Taking c=1 proves the stated optimum. These values were also checked independently by the two finite numerical procedures used throughout Section 14; for B, multistart Frank–Wolfe and exhaustive permutation search both return 1/9. With λ=0.01, the three objectives are*

J(A)=0.0900,J(B)=0.1111+0.0300=0.1411,J(C)=0.6667+0.0100=0.6767,

*so the exact but longer model A is selected. If λ is increased to 0.02, then J(A)=0.1800 and J(B)=0.1711, so the shorter equilateral model B is selected. This trace shows exactly where relational fit, code length, and the MDL scale enter the decision.*

The complexity penalty is the MDL term. Two implementation requirements are validated in Section 14: *fitted flexibility must be penalized* (the experiments use the BIC-style score $\frac{1}{2}\log_{2}n$ per fitted real; with only the Kraft-valid model-index component, a memorizing candidate wins the latent-structure-recovery control), and *λ must be selected without using the final test relations*. We use cross-validated relational risk on fit/validation splits. At larger scale, the reported confirmation frequency uses a separately disclosed supervised calibration stage with known synthetic truth followed by 40 fresh corpora at the frozen penalty. These devices prevent silent tuning on the confirmation set, but do not turn the BIC score into a literal parameter code or provide an unsupervised calibration rule for unknown real-world structure.

### 7.3. A Code-Aware Collapse Diagnostic

The *k*-block experiments use the declared score

(6) $$C_{n}\left(k\right)=1-\log_{2}\;\left(\frac{6}{\pi^{2}k^{2}}\right)+\frac{1}{2}q_{k}\log_{2}n,\;q_{k}=\frac{k(k+1)}{2}.$$

The first term is a one-bit family flag plus a Kraft-valid code over the integer index *k*. The second is the implemented BIC/MDL dimension charge for the declared symmetric block parameters. It is not a literal prefix code for an arbitrary partition or for unquantized fitted reals. If the partition must be transmitted, its code must be added; if singleton blocks have no fitted within-block parameter, the effective $q_{k}$ should be reduced. The numerical threshold below must always be recomputed from the *actual declared penalty*. For (6), the smallest gap above k=1 occurs at k=2 and is

(7) $$\Delta_{C}^{\mathrm{decl}}\left(n\right)=C_{n}\left(2\right)-C_{n}\left(1\right)=2+\log_{2}n.$$

The following finite calculation isolates what this score can force. Because compressor dissimilarities need not satisfy the triangle inequality, “GW” in this diagnostic means the finite network-GW objective unless metricity has separately been established.

**Lemma 2** (One-block network-distortion identity)**.**  *Let n≥2 and let $D\in R^{n\times n}$ be non-negative, symmetric, and zero-diagonal, with uniform mass. Write*

$$\bar{d}=\frac{1}{n(n-1)}\sum_{i\neq j}D_{ij},\;V_{\mathrm{off}}\left(D\right)=\frac{1}{n(n-1)}\sum_{i\neq j}{\left(D_{ij}-\bar{d}\right)}^{2}.$$

*Let $M_{1}$ be the n-point equilateral blow-up with ${\left(M_{1}\right)}_{ab}=\bar{d}\;1\left\{a\neq b\right\}$. Then,*

(8) $$d_{\mathrm{GW},2}^{2}\left(D,M_{1}\right)=B\left(D\right):=\frac{1}{2}\left(1-\frac{1}{n}\right)V_{\mathrm{off}}\left(D\right).$$

**Proof.**  Put $s_{ij}=\sum_{a}\pi_{ia}\pi_{ja}$. Expanding the objective gives

$$2d_{\mathrm{GW},2}^{2}=\underset{\pi }{\inf}\left[C-{\bar{d}}^{2}\sum_{i}s_{ii}+\bar{d}\sum_{i\neq j}\left(2D_{ij}-\bar{d}\right)s_{ij}\right],\;C=\frac{{\bar{d}}^{2}}{n}+\left(1-\frac{1}{n}\right)V_{\mathrm{off}}.$$

The target marginal gives $\sum_{ij}s_{ij}=1/n$, hence $\sum_{i}s_{ii}=1/n-\sum_{i\neq j}s_{ij}$. Substitution cancels the apparently troublesome off-diagonal coefficient and yields

$$2d_{\mathrm{GW},2}^{2}=\underset{\pi }{\inf}\left[C-\frac{{\bar{d}}^{2}}{n}+2\bar{d}\sum_{i\neq j}D_{ij}s_{ij}\right]\geq C-\frac{{\bar{d}}^{2}}{n}=\left(1-\frac{1}{n}\right)V_{\mathrm{off}}\left(D\right),$$

since $D_{ij},\bar{d},s_{ij}\geq 0$. A permutation coupling $\pi_{ia}=n^{-1}1\left\{a=\sigma \left(i\right)\right\}$ has $s_{ij}=0$ for i≠j and attains the lower bound. Dividing by two proves (8) without a separation condition.    □

**Proposition 2** (Penalty-domination certificate)**.**  *Consider a k-block candidate menu containing $M_{1}$ and let $C_{k}$ be its declared penalties. Suppose*

$$\Delta_{C}:=\underset{k\geq 2}{\inf}\left(C_{k}-C_{1}\right)>0.$$

*If*

(9) $$\lambda \Delta_{C}>B\left(D\right),$$

*then every minimizer of the unmarked distortion-plus-penalty objective over that menu has k=1.*

**Proof.**  For every k≥2, non-negativity of the distortion and Lemma 2 give

$$J\left(1\right)-J\left(k\right)\leq B\left(D\right)-\lambda \left(C_{k}-C_{1}\right)\leq B\left(D\right)-\lambda \Delta_{C}<0.$$

Thus, k=1 strictly dominates every other member of the menu.    □

Four scope restrictions are essential. First, $M_{1}$ is an *n*-point equilateral blow-up coded by one block parameter, not the honest one-point quotient. Against the one-point quotient, the distortion is

$$\frac{1}{2}\left(1-\frac{1}{n}\right)\left(V_{\mathrm{off}}\left(D\right)+{\bar{d}}^{2}\right),$$

which can be much larger. Second, Proposition 2 concerns the declared *k*-block menu. A tree, raw-geometry, or other candidate with a smaller penalty gap must be analyzed with its own $\Delta_{C}$. Third, the certificate is scale-dependent: D↦cD sends B(D) to $c^{2}B\left(D\right)$ while leaving the code unchanged. It is meaningful only after the distance normalization and λ calibration have been fixed. Fourth, the threshold $\lambda_{★}=B\left(D\right)/\Delta_{C}$ depends on both sample size and the empirical distance dispersion. For the implemented score, increasing *n* raises $\Delta_{C}^{\mathrm{decl}}\left(n\right)=2+\log_{2}n$, while a representation change that shrinks $V_{\mathrm{off}}$ lowers B(D); either effect can make the sufficient condition easier to satisfy at fixed λ. This is not a universal monotonicity law, as the distribution of *D* may itself change. The controlled elementary cellular automaton (ECA) audit reported in Section 14 supports recalibrating λ whenever the sample size, object representation, or distance normalization changes.

Most importantly, (9) diagnoses the *objective and candidate family*, not the information content of the data. Small $V_{\mathrm{off}}$ indicates that the one-block blow-up is cheap under the particular squared relational loss, not that local neighborhoods, marks, or other statistics of *D* lack predictive signal. The cellular-automaton and SMS prediction studies in Section 14 exhibit exactly this distinction.

### 7.4. Marked Objective

For labeled data, let each candidate geometry carry a candidate-side mark map $m_{\theta }:Z_{\theta }\to Y$, declared in advance or estimated using construction/training data before the marked coupling is solved. This map is distinct from the downstream predictor fitted after kernelization. Given a coupling π between ${\hat{\mu }}_{n}$ and $\mu_{\theta }$, define

$$L_{\mathrm{mark}}\left(\pi ,\theta \right)=\int_{X_{n}\times Z_{\theta }}L\left(y\left(x\right),m_{\theta }\left(z\right)\right)\;d\pi \left(x,z\right),$$

where L is the pointwise prediction loss. This notation keeps the loss distinct from the label map $\ell_{n}$ used in the marked empirical space.

**Definition 6** (Marked Solomonoff–Gromov objective)**.**  *The supervised or marked objective is*

(10) $$\begin{array}{cc} J_{n}^{\mathrm{mark}}\left(\theta \right)=\underset{\pi \in \Pi ({\hat{\mu }}_{n},\mu_{\theta })}{\inf}\; & [\frac{1}{2}\int {\left|{\hat{d}}_{\mathrm{Sol}}\left(x,x^{\prime }\right)-d_{\theta }\left(z,z^{\prime }\right)\right|}^{p}\;d\pi \left(x,z\right)\;d\pi \left(x^{\prime },z^{\prime }\right)\\ \; & \;+\eta \int L(y\left(x\right),m_{\theta }\left(z\right))\;d\pi \left(x,z\right)]+\lambda \;\mathrm{Code}\left(\theta \right). \end{array}$$

This objective matches both algorithmic geometry and task structure. When η=0, it reduces to the unsupervised objective. Structurally, it is the Solomonoff/MDL version of a Fused Gromov–Wasserstein objective: one term aligns pairwise relational geometry and the other aligns attached marks or labels through the same coupling. Existing FGW solvers can be used as implementation templates, with the code penalty and algorithmic distance supplying the Solomonoff-specific layer.

If we want to learn $m_{\theta }$ jointly with the coupling, then it is a different alternating or joint optimization problem and must be stated as such. The implemented labeled block experiments in Section 14 use training-derived block marks before solving marked GW. The later KRR predictor is then fitted separately on the selected kernel.

### 7.5. Interpretation

The optimal coupling $\pi^{★}$ is a soft correspondence between observed data and points in the explanatory geometry. If *z* is a latent program, then $\pi^{★}\left(x,z\right)$ says that program *z* explains data point *x*. If *z* is a node in a prefix tree, the coupling aligns data objects with branches of the algorithmic hierarchy. If *z* is a point on a manifold, then the coupling identifies the manifold coordinates whose internal distances best reproduce the empirical Solomonoff distances.

Therefore, the GW term plays the role of an *algorithmic structural likelihood* that states how well the candidate model reproduces the algorithmic relational pattern in the data.

## 8. Category-Native Comparison Functionals: The Broad Tour

This is the first systematic sweep of the category-native programme. The tour is repeated at two different levels by design: comparison functionals are specified here, kernelization or representation functors in Section 11, and induction rules later in that section. These are three columns of one architecture, not three interchangeable descriptions. Each paragraph below cross-references the corresponding representational and predictive constructions rather than treating them as consequences of the comparison alone.

There is a hierarchy of ways to compare structured objects: isometry, bi-Lipschitz distortion, Gromov–Hausdorff (GH), Gromov–Wasserstein (GW), Wasserstein/MMD on a shared space, and operator-spectral comparison. They are often presented as competitors; in the present framework, however, they control different parts of the recovered object, so the principled construction may be layered rather than governed by a single scalar functional.

- **Isometry** is the ideal, not a separate computational tool. For mm-spaces, $d_{\mathrm{GW}}=0$ if and only if the spaces are measure-preserving isometric [28,29]. Isometry is the perfect score of the GW term.
- **Gromov–Hausdorff versus Gromov–Wasserstein.** GH compares metric geometry without choosing a measure; GW compares metric probability spaces and is sensitive to mass. Neither criterion forces a particular weighting: uniform, empirical, and Occam masses are all possible in GW. A KC or Solomonoff kernel additionally requires the declared landmark weighting of Propositions 8 and 9. GH remains a measure-free geometric check, not a prescription for uniform mass.
- **Operator-spectral comparison**, either $d_{\mathrm{spec}}$ of Section 16 or a richer heat kernel/diffusion comparison, targets the eigenvalue profile, and hence the Occam-calibration layer. It complements relational GW.
- **Wasserstein and MMD** require a shared ambient space, and as such do not directly compare the empirical Solomonoff space with an arbitrary candidate. After kernelization, however, candidates can be represented in feature spaces, and a basis-sensitive discrepancy or task loss becomes natural for the marking or predictive layer of (10).

### Category-by-Category Comparison Functionals

The practical rule is as follows:

$$\begin{array}{c} \mathrm{use}\;\mathrm{GW}\;\mathrm{when}\;\mathrm{both}\;\mathrm{objects}\;\mathrm{are}\;\mathrm{represented}\;\mathrm{as}\;\mathrm{metric}–\mathrm{measure}\;\mathrm{spaces}. \end{array}$$

**Figure 1:**
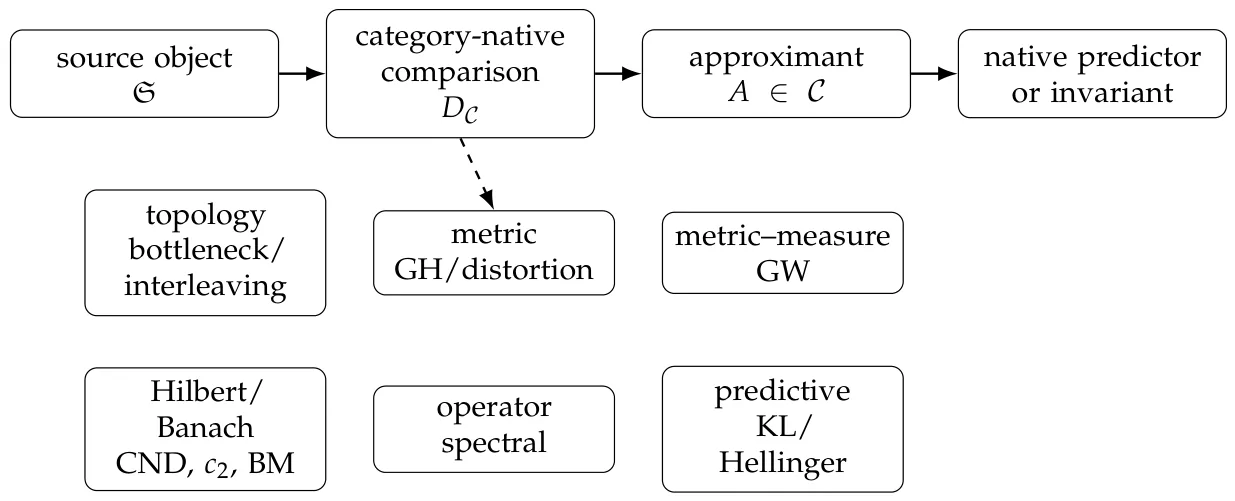
Category-native approximation separates the source, comparison functional, structured approximant, and resulting invariant or predictor.

Otherwise, the invariant that is native to the structure being preserved should be used, and should be attached to the common architecture (1). This does not demote GW to an arbitrary menu item; GW is the relational mm-space functional, and the topology, normed linear structure, spectra, and prediction all have different invariances. Figure 1 depicts the shared architecture.

**Topological targets.** A bare topological space (X,τ) has no distances and no probability measure, so GW is not defined on it. Homeomorphism, homotopy equivalence, and shape equivalence are usually too rigid for approximation. Thus, a practical topological comparison passes through a signature:

(X,τ)⟼topologicalsignature⟼distancebetweensignatures.

For example, choose a filtration $F_{X}\left(r\right)$, compute the persistent homology ${\mathrm{PH}}_{k}\left(F_{X}\right)$ [53,54,55], and compare the persistence diagrams [56] by

$$D_{\mathrm{Top}}\left(X,Y\right)=\sum_{k}w_{k}\;d_{\mathrm{bottle}}\left({\mathrm{Dgm}}_{k}\left(F_{X}\right),{\mathrm{Dgm}}_{k}\left(F_{Y}\right)\right);$$

alternatively, use interleaving distance [57] when the filtration-level structure is the object of interest. Natural Solomonoff-side filtrations include algorithmic distance thresholds, prefix depth, description-length thresholds, posterior mass thresholds, and algorithmic mutual information thresholds. This tests preservation of connected components, holes, branches, persistent clusters, and multiscale topology. If a compatible metric and measure are imposed, the object re-enters the mm-space layer and GW applies to that representation.

**Metric targets without measure preservation.** If only distances matter, use Gromov–Hausdorff or Lipschitz distortion rather than GW:

$$d_{\mathrm{GH}}\left(X,Y\right),\;c\left(X,Y\right)=\underset{\phi }{\inf}\mathrm{Lip}\left(\phi \right)\mathrm{Lip}\left(\phi^{-1}\right).$$

Thus, GH is metric-only calibration, whereas GW is metric-plus-mass calibration. In the BAITML setting, $d_{\mathrm{GH}}$ is appropriate for KC-style relational geometry that does not commit to Occam mass, while $d_{\mathrm{GW}}$ is appropriate when probability weights such as $\mu \left(x\right)\propto 2^{-K(x)}$ are part of the model.

**Hilbert targets.** For Hilbert approximations, a supplement to GW is Hilbert distortion. Given (X,d), seek ϕ:X→H satisfying

$$a\;d\left(x,y\right)\leq {∥\phi \left(x\right)-\phi \left(y\right)∥}_{H}\leq b\;d\left(x,y\right)$$

and minimize $c_{2}\left(X,d\right)=\inf_{\phi }b/a$. Equivalently, a metric is Hilbertian when $d^{2}$ is conditionally negative definite, so one may measure the distance of $d^{2}$ to the CND cone. This is the structural diagnostic behind the Hilbert gap function in Section 9. GW measures mass-weighted relational cost, while Hilbert distortion or CND defect measures what is lost by forcing the object into Hilbert form.

**Banach targets.** For finite-dimensional normed spaces E,F, the classical linear comparison is the Banach–Mazur distance:

$$d_{\mathrm{BM}}\left(E,F\right)=\underset{T:E\to F}{\inf}∥T∥\;∥T^{-1}∥.$$

For a metric object represented inside a Banach space *B*, the flexible analogue is the Lipschitz distortion:

$$c_{B}\left(X,d\right)=\underset{\phi :X\to B}{\inf}\mathrm{Lip}\left(\phi \right)\mathrm{Lip}\left(\phi^{-1}\right).$$

This makes it meaningful to ask whether a Solomonoff geometry is better represented in $L^{1}$, $L^{2}$, a variation-norm/Barron space [58,59,60], or another Banach geometry. After a Banach candidate is represented as $\left(Z,∥z-z^{\prime }{∥}_{B},\mu_{Z}\right)$, GW can score its relational mm-space fit; the Banach distortion states what is gained or lost by insisting on that normed-linear presentation.

**Spectral and operator targets.** Once a geometry has been kernelized, operator distances become natural. For kernel or covariance operators $T_{1},T_{2}$, one can use

$$∥T_{1}-T_{2}{∥}_{\mathrm{HS}},\;∥T_{1}-T_{2}{∥}_{\mathrm{tr}},\;d_{\mathrm{spec}}^{2}\left(T_{1},T_{2}\right)=\sum_{i}{\left|\lambda_{i}\left(T_{1}\right)-\lambda_{i}\left(T_{2}\right)\right|}^{2}.$$

This is the appropriate calibration when the target is the complexity spectrum $\lambda_{i}\approx 2^{-\ell_{i}}$. GW checks relational structure before or at kernelization, while spectral distance checks whether the induced operator has the desired Occam/Solomonoff eigenvalue profile.

**Predictive targets.** If the goal is to approximate Solomonoff as a predictor, then the native comparison is predictive divergence rather than GW. Solomonoff convergence theory is built around cumulative KL,

$$\sum_{t=1}^{n}E_{\mu }D_{\mathrm{KL}}\;\left(\mu \left(\cdot |x_{<t}\right)\;∥\;\xi \left(\cdot |x_{<t}\right)\right),$$

and Hellinger gives a symmetric Hilbertian proxy,

$$H^{2}\left(p,q\right)=\frac{1}{2}\sum_{a}{\left(\sqrt{p(a)}-\sqrt{q(a)}\right)}^{2}.$$

GW asks whether a candidate preserves relational algorithmic geometry; predictive divergence asks whether it makes the same sequential predictions. The two questions coincide only under an additional transfer argument. Table 6 summarizes the native comparisons and the structures they are intended to preserve.

**The layered criterion.** A faithful surrogate may require three calibrations, matching three distinct factors:

$$\underset{\mathrm{relational}\;\mathrm{feature}\;\mathrm{dictionary}}{\underset{︸}{d_{\mathrm{GW}}\;\mathrm{small}}}\;+\;\underset{\mathrm{Occam}\;\mathrm{weights}\;\mathrm{and}\;\mathrm{spectrum}}{\underset{︸}{d_{\mathrm{spec}}\;\mathrm{small}}}\;+\;\underset{\mathrm{prediction}}{\underset{︸}{\mathrm{marked}/\mathrm{task}\;\mathrm{discrepancy}\;\mathrm{small}}}.$$

GW alone is neither a spectral nor a predictive guarantee. The middle label must also be read with Remark 7: the raw dictionary is fixed by the distance, whereas eigenfunctions depend jointly on that dictionary and its weights. Near-orthogonality controls eigenvalues; stability of individual eigenfunctions additionally requires spectral gaps.

**Scale invariance and the distortion viewpoint.** Bi-Lipschitz distortion is scale-invariant, whereas $L^{p}$ GW is not. This matters because a compressor-induced distance has a normalization-dependent scale. Two practical robustifications are to normalize each candidate distance matrix, for example by its diameter, before computing GW, or to use a relative/log cost

$$|\log\left(d_{X}\vee \epsilon_{0}\right)-\log\left(d_{Y}\vee \epsilon_{0}\right){|}^{p}$$

with a fixed floor $\epsilon_{0}>0$. The floor is necessary because couplings that split atomic mass encounter diagonal distances equal to zero. These choices define modified comparison problems and can change candidate rankings; the normalization and floor must therefore be declared before selection.

**Remark 3** (The relational layer is an inner product)**.**  *For p=2, the finite GW objective factorizes [30] as*

$$\sum_{i,i^{\prime },a,a^{\prime }}{\left(D_{ii^{\prime }}^{X}-D_{aa^{\prime }}^{Y}\right)}^{2}\Pi_{ia}\Pi_{i^{\prime }a^{\prime }}=\underset{\kappa (\alpha ,\beta ),\;\mathrm{independent}\;\mathrm{of}\;\Pi }{\underset{︸}{\alpha^{⊤}\left(D^{X}\;\circ \;D^{X}\right)\alpha +\beta^{⊤}\left(D^{Y}\;\circ \;D^{Y}\right)\beta }}-2{\langle D^{X},\Pi D^{Y}\Pi^{⊤}\rangle }_{F}.$$

*Thus minimizing finite $d_{\mathrm{GW},2}^{2}$ maximizes the Frobenius alignment ${\langle D^{X},\Pi D^{Y}\Pi^{⊤}\rangle }_{F}$. The relational comparison and its optimization are two expressions of the same alignment problem.*

## 9. Boundary Diagnostics for Metric–Measure Projections

This section records boundary diagnostics for forcing algorithmic or predictive explanation geometry into restricted metric–measure subclasses. They establish the limits of the developed mm-space realization. While they do not prove the other branches of Table 4, they do illustrate the category-native premise that different projections discard different information.

### 9.1. Algorithmic, Predictive, and Hybrid Explanation Distances

There is no single canonical distance on explanations. On syntactic descriptions, normalized algorithmic information gives the dissimilarity

$$a_{\mathrm{alg}}\left(h,g\right)=\frac{\max\{K(h|g),K(g|h)\}}{\max\{K(h),K(g)\}}$$

for program-theoretic convertibility, restricting to descriptions with positive denominator. It is not automatically an exact metric and is not invariant under semantic equivalence. For a metric–measure construction, fix a common support *Q* and a bounded genuine metric $d_{\mathrm{alg}}$ on it, for example a finite metric repair of this dissimilarity on a declared dictionary, followed by completion. Claims about this repaired metric do not assert an exact identification with normalized information distance. On the same support define

$$d_{\mathrm{pred}}^{2}\left(h,g\right)=\sum_{t\geq 1}\beta_{t}\;E_{x_{<t}∼M_{D}}H^{2}\;\left(\nu_{h}\left(\cdot |x_{<t}\right),\nu_{g}\left(\cdot |x_{<t}\right)\right)$$

for predictive behavior, using a common, candidate-independent history law and probability conditionals (including a termination symbol for semimeasures). Histories with zero reference probability may be assigned arbitrary common versions. Quotient by zero predictive distance when using $d_{\mathrm{pred}}$ alone. For α>0, the hybrid on *Q* is

$$d_{\alpha }=\alpha d_{\mathrm{alg}}+\left(1-\alpha \right)d_{\mathrm{pred}},\;\alpha \in \left[0,1\right].$$

Throughout this display, ${\left(\beta_{t}\right)}_{t\geq 1}$ is a fixed deterministic discount sequence with $\beta_{t}\geq 0$ and $\sum_{t\geq 1}\beta_{t}<\infty$; for example, one may take $\beta_{t}=\left(1-\rho \right)\rho^{t-1}$ with 0<ρ<1. This summability condition makes $d_{\mathrm{pred}}$ finite whenever the one-step Hellinger losses are uniformly bounded, as one may normalize $\sum_{t}\beta_{t}=1$. The predictive distance is Hilbertian through the square-root embedding of probability laws. The chosen exact metric $d_{\mathrm{alg}}$ need not be Hilbertian. Its Schoenberg defect must be checked for that metric; an obstruction for unnormalized information distance is not a proof for every normalized or compressor-based surrogate.

### 9.2. The Hilbert Gap Function

Define the Hilbert-restricted approximation gap

(11) $$g\left(\alpha \right)=\underset{M\in C_{\mathrm{Hilb}}}{\inf}d_{\mathrm{GW},p}\left((Q,d_{\alpha },\pi_{D}),M\right),$$

For every α, take the metric completion; at α=0, first take the zero-distance quotient, always pushing forward the same posterior probability. Here $C_{\mathrm{Hilb}}$ is the class of metric–measure spaces realized as subsets of Hilbert space with their Hilbert distance and a probability measure.

**Proposition 3** (Endpoint and perturbation structure of *g*)**.**  *Assume that the predictive metric $d_{\mathrm{pred}}$ is represented exactly in $C_{\mathrm{Hilb}}$; then, g(0)=0. Moreover, for all $\alpha ,\alpha^{\prime }\in \left[0,1\right]$,*

$$|g\left(\alpha \right)-g\left(\alpha^{\prime }\right)|\leq 2^{-1/p}|\alpha -\alpha^{\prime }|\;∥d_{\mathrm{alg}}-d_{\mathrm{pred}}{∥}_{\infty }.$$

*If the source has finite support and the infimum is restricted to Hilbert realizations on that fixed support with the same mass vector, then a positive posterior-mass obstruction to Hilbert embeddability, for example, a positive mass-weighted CND defect, implies g(1)>0 for this fixed-support/mass restriction.*

**Proof.**  The equality g(0)=0 follows by choosing the exact Hilbert realization of the predictive-Hellinger geometry. The Lipschitz bound is the GW distance perturbation estimate used in Section 10, together with

$$∥d_{\alpha }-d_{\alpha^{\prime }}{∥}_{\infty }\leq |\alpha -\alpha^{\prime }|\;∥d_{\mathrm{alg}}-d_{\mathrm{pred}}{∥}_{\infty }.$$

For the final statement discard zero-mass points and write $m_{i}>0$ for the fixed masses. On an objective sublevel, Minkowski’s inequality under any coupling gives

$$∥D_{Y}{∥}_{L^{p}\left(m⊗m\right)}\leq {∥D_{X}∥}_{L^{p}\left(m⊗m\right)}+2^{1/p}d_{\mathrm{GW},p}\left(X,Y\right).$$

Thus every entry of $D_{Y}$ is bounded. The Hilbertian pseudometric matrices form a closed set: their squared matrices are conditionally negative definite. A minimizing sequence therefore has a convergent subsequence in this bounded finite-dimensional set. If the infimum were zero, the limiting pseudometric quotient would be GW-isomorphic to the source. That quotient is Hilbertian, contradicting the assumed non-Hilbertian source. This proves positivity; attainment is asserted in the pseudometric closure, not necessarily among strictly positive metrics on the original labeled support.    □

The function *g* is a compact diagnostic for how much of the posterior geometry is genuinely algorithmic rather than merely predictive. It also prevents the reader from mistaking an exact Hilbert embedding of an ultrametric or Hellinger geometry for evidence that all Solomonoff-side geometry is Hilbertian.

### 9.3. Gaussian Atom Gap

A Gaussian or SGP approximation retains covariance geometry but discards atomic and higher-order posterior structure. This mismatch can be measured before kernelization. Let

$$\alpha \left(\mu \right)=\sum_{x\in \mathrm{Atoms}(\mu )}\mu {\left(\left\{x\right\}\right)}^{2}.$$

This is the mass at zero in the distance distribution $d_{\#}\left(\mu ⊗\mu \right)$.

**Remark 4** (Gaussian atom-gap question)**.**  *Fix p≥1, a dimension r≥1, constants 0<a≤b<∞, and a bounded source S. A precise restricted question concerns*

$$\underset{aI_{r}⪯\Sigma ⪯bI_{r}}{\inf}d_{\mathrm{GW},p}\left(S,(R^{r},∥\cdot {∥}_{2},N\left(0,\Sigma \right))\right).$$

*This infimum is positive when the source has an atom: the covariance set is compact, its Gaussian mm-spaces vary continuously in GW, and every candidate is nonatomic, whereas zero GW identifies metric probability spaces. The finite-dimensional Gaussian continuity follows by coupling $\Sigma^{1/2}Z$ with $\Sigma^{\prime 1/2}Z$. This restricted observation does not give a dimension-free lower bound from atom mass and distance spread alone; such a quantitative extension is left open.*

The empirical statistic $\hat{\alpha }=\sum_{i}{\hat{\mu }}_{i}^{2}=\exp\left(-H_{2}\left(\hat{\mu }\right)\right)$ measures posterior concentration. It detects atomic structure but does not determine all non-Gaussian features. The maximum-entropy characterization of a Gaussian applies to Euclidean densities with a fixed covariance and finite differential entropy; it is not an identity for arbitrary atomic metric–measure spaces.

## 10. Well-Posedness and Stability

### 10.1. Finite Candidate Classes

**Proposition 4** (Existence in a finite class)**.**  *Let $G=\{M_{1},\ldots ,M_{N}\}$ be a nonempty finite family of finite mm-spaces with finite code penalties. Then, the Solomonoff–Gromov objective (5) has a minimizer.*

**Proof.**  For each candidate $M_{j}$, the finite GW problem is a continuous optimization problem over a compact transportation polytope; hence, the infimum defining $d_{\mathrm{GW},p}$ is attained. Since the model class is finite, the minimum over j=1,…,N is attained.    □

### 10.2. Compact Parameter Families

**Assumption 1** (Compact continuous family)**.**  *Assume that* Θ *is nonempty and compact, the penalty multiplier is nonnegative, the code is bounded below and finite at at least one candidate, each $M_{\theta }=\left(Z,d_{\theta },\mu_{\theta }\right)$ is defined on a common compact set Z, $d_{\theta }$ is continuous in $\left(\theta ,z,z^{\prime }\right)$, $\theta \mapsto \mu_{\theta }$ is weakly continuous, and Code(θ) is lower semicontinuous.*

**Proposition 5** (Existence in compact families)**.**  *Under the compact continuous family assumption, the objective $J_{n}$ admits a minimizer.*

**Proof.**  For bounded compact metric spaces, $d_{\mathrm{GW},p}$ is continuous under uniform convergence of distances and weak convergence of measures. Therefore, $\theta \mapsto d_{\mathrm{GW},p}^{p}\left({\hat{S}}_{n},M_{\theta }\right)$ is continuous, while the code penalty is lower semicontinuous. The sum is lower semicontinuous on compact Θ, and hence attains its minimum.    □

### 10.3. Stability to Compressor Perturbations

Let $\hat{d}$ and $\tilde{d}$ be two empirical algorithmic distances on the same sample $X_{n}$. Suppose

$$∥\hat{d}-\tilde{d}{∥}_{\infty }=\max_{i,j}\left|\hat{d}\left(x_{i},x_{j}\right)-\tilde{d}\left(x_{i},x_{j}\right)\right|\leq \varepsilon .$$

**Proposition 6** (Stability of GW to empirical distance perturbations)**.**  *For every candidate mm-space M,*

$$\left|d_{\mathrm{GW},p}\left(\;\left(X_{n},\hat{d},{\hat{\mu }}_{n}\right),M\;\right)-d_{\mathrm{GW},p}\left(\;\left(X_{n},\tilde{d},{\hat{\mu }}_{n}\right),M\;\right)\right|\leq 2^{-1/p}\varepsilon .$$

*Consequently, the unpowered GW distance is Lipschitz-stable with respect to uniform perturbations of the empirical algorithmic distance. Since the objective $J_{n}$ uses $d_{\mathrm{GW},p}^{p}$, the powered distance has the corresponding local Lipschitz bound*

$$|a^{p}-b^{p}|\leq pR^{p-1}\left|a-b\right|$$

*whenever a,b≤R. Thus, on geometry classes with uniformly bounded diameter, the displayed perturbation bound induces a finite Lipschitz constant for the objective itself; the constant depends on p and the diameter bound.*

**Proof.**  For any coupling π,

$$\left|\right|\hat{d}\left(x,x^{\prime }\right)-d\left(y,y^{\prime }\right)\left|\;-\;\right|\tilde{d}\left(x,x^{\prime }\right)\;-\;d\left(y,y^{\prime }\right)\left||\;\leq \;\right|\hat{d}\left(x,x^{\prime }\right)\;-\;\tilde{d}\left(x,x^{\prime }\right)|\;\leq \;\varepsilon .$$

Taking $L^{p}\left(\pi ⊗\pi \right)$ norms, infimizing over π, and using the factor 1/2 in the definition gives the claim.    □

**Remark 5.**
  *This proposition is important practically. It says that changing the compressor or the finite approximation to Kolmogorov complexity perturbs the GW score only as much as it perturbs the pairwise algorithmic distance matrix. Therefore, cross-compressor stability can be reported as an empirical confidence measure.*

## 11. Category-Native Kernelization and Induction

Section 8 specifies what it means to compare the Solomonoff-side source with objects in different categories. This section completes the positional argument by specifying what the selected object induces. The metric–measure branch is developed first and carries the proved Hilbertian core. The later category-specific subsections are constructional dictionaries that state the native representation and prediction rule for each branch without silently exporting the mm-space theorems to it.

### 11.1. The Induced D2KE Kernel

Once $\hat{M}=\left(Z_{\hat{\theta }},d_{\hat{\theta }},\mu_{\hat{\theta }}\right)$ has been selected, define

$$k_{\hat{\theta }}\left(z,z^{\prime }\right)=\int_{Z_{\hat{\theta }}}e^{-\gamma d_{\hat{\theta }}\left(z,\omega \right)}e^{-\gamma d_{\hat{\theta }}\left(z^{\prime },\omega \right)}\;d\mu_{\hat{\theta }}\left(\omega \right).$$

**Proposition 7** (Positive definiteness of the induced kernel)**.**  *For any metric–measure space (Z,d,μ) and any γ>0, the D2KE kernel*

$$k\left(z,z^{\prime }\right)=\int_{Z}e^{-\gamma d(z,\omega )}e^{-\gamma d(z^{\prime },\omega )}\;d\mu \left(\omega \right)$$

*is positive semidefinite.*

**Proof.**  For finite coefficients $c_{1},\ldots ,c_{m}$ and points $z_{1},\ldots ,z_{m}$,

$$\sum_{i,j}c_{i}c_{j}k\left(z_{i},z_{j}\right)=\int_{Z}{\left(\sum_{i}c_{i}e^{-\gamma d(z_{i},\omega )}\right)}^{2}d\mu \left(\omega \right)\geq 0.$$

   □

Thus, the selected geometry induces an RKHS, kernel ridge regression estimators, GP priors, and kernel mean embeddings without requiring the original algorithmic distance to be positive definite.

### 11.2. Metric–Measure Payoff: Recovering the Earlier Kernels

The geometry-first construction recovers the earlier kernel papers by making two independent choices:

$$\begin{array}{c} \mathrm{distance}\;d\;\mathrm{sets}\;\mathrm{the}\;\mathrm{relational}\;\mathrm{features},\;\mathrm{measure}\;\mu \;\mathrm{sets}\;\mathrm{their}\;\mathrm{weights}. \end{array}$$

Thus, the recovery is not a new derivation of a different object, it is a factorization of the old constructions through the selected geometry:

$$\begin{array}{ccc} \mathrm{uniform}\;\mathrm{mass}\;\mathrm{on}\;\mathrm{algorithmic}\;\mathrm{landmarks} & ⟹ & \mathrm{KC}/D2\mathrm{KE}\;\mathrm{kernel},\\ \mathrm{Occam}\;\mathrm{mass}\;\mathrm{on}\;\mathrm{the}\;\mathrm{same}\;\mathrm{landmarks} & ⟹ & \mathrm{Solomonoff}\;\mathrm{Feature}\;\mathrm{Mixture},\\ \mathrm{near}\text{-}\mathrm{orthogonal}\;\mathrm{weighted}\;\mathrm{features} & ⟹ & \mathrm{Solomonoff}\;\mathrm{spectral}\;\mathrm{law}\;\lambda_{i}≍2^{-\ell_{i}}. \end{array}$$

The induced kernel

(12) $$k_{\left(d,\mu \right)}\left(z,z^{\prime }\right)=\int_{Z}\phi_{\omega }\left(z\right)\;\phi_{\omega }\left(z^{\prime }\right)\;d\mu \left(\omega \right),\;\phi_{\omega }\left(z\right)=e^{-\gamma d(z,\omega )}$$

is a *master template* that is parametrized by a *distance d* and *landmark measure* μ. The two classical objects of the programme are exactly the two canonical choices of the pair (d,μ), and the geometry-first pipeline returns each as a special case.

**Proposition 8** (KC-kernel recovery)**.**  *Take the selected geometry to be the empirical Solomonoff space itself, $\hat{M}={\hat{S}}_{n}=\left(X_{n},{\hat{d}}_{\mathrm{Sol}},{\hat{\mu }}_{n}\right)$, with ${\hat{\mu }}_{n}$ the uniform empirical measure. Then (12) is the uniform-landmark D2KE kernel of ${\hat{d}}_{\mathrm{Sol}}$. It recovers the KC construction when its features and normalization match those in Parts II–III. In the Solomonoff–Gromov objective, this is the degenerate selection $M_{\theta }={\hat{S}}_{n}$ (zero relational distortion, $d_{\mathrm{GW}}=0$) with uniform mass. Zero distortion does not force its selection when a positive complexity penalty is present.*

**Proposition 9** (Solomonoff/SFM kernel recovery and normalization)**.**  *On a nonempty countable dictionary, let $w_{\omega }=2^{-\ell (\omega )}$ with $0<W=\sum_{\omega }w_{\omega }\leq 1$. Define the finite measure $\nu_{\mathrm{Sol}}=\sum_{\omega }w_{\omega }\delta_{\omega }$ and its probability normalization $\mu_{\mathrm{Sol}}=\nu_{\mathrm{Sol}}/W$. Then*

$$k_{\mathrm{SFM}}\left(z,z^{\prime }\right)=k_{\left(d,\nu_{\mathrm{Sol}}\right)}\left(z,z^{\prime }\right)=\sum_{\omega }w_{\omega }\phi_{\omega }\left(z\right)\phi_{\omega }\left(z^{\prime }\right),\;k_{\left(d,\mu_{\mathrm{Sol}}\right)}=W^{-1}k_{\mathrm{SFM}}.$$

*GW uses the probability measure $\mu_{\mathrm{Sol}}$. The exact spectral identities in Theorem 2 use unnormalized feature weights $w_{\omega }$. Normalization multiplies all eigenvalues by $W^{-1}$; this does not alter comparability for a fixed dictionary, but W must be tracked when dictionaries vary. These are uncentered second moments, distinct from posterior feature covariance unless the feature mean is zero. Identifications with other parts of the series require the same feature and normalization conventions.*

**Proof.**  Both identities follow by substituting the discrete measures in Equation (12); linear scaling of the kernel scales its integral operator and eigenvalues by the same factor.    □

**Theorem 1** (Kolmogorov–Solomonoff unification)**.**  *Fix an algorithmic distance d on a landmark set* Ω *and the D2KE feature family*

$$\phi_{\omega }\left(z\right)=e^{-\gamma d(z,\omega )}.$$

*For a finite nonnegative landmark measure μ, let*

$$k_{\left(d,\mu \right)}\left(z,z^{\prime }\right)=\int_{\Omega }\phi_{\omega }\left(z\right)\phi_{\omega }\left(z^{\prime }\right)\;d\mu \left(\omega \right)$$

*be the master kernel (12). Then:*

*1.*  *(Shared relational feature family). The raw feature maps $\phi_{\omega }$, and hence the relational information made available to the kernelization, are determined by d. Changing μ changes the weighting of this fixed family, but need not leave the covariance-operator eigenfunctions literally unchanged without orthogonality or a perturbation bound with spectral gaps.*
*2.*  *(Two canonical measures). With empirical or uniform landmark mass, $k_{\left(d,\mu_{\mathrm{unif}}\right)}$ is the Kolmogorov/D2KE kernel of Parts II–III (Proposition 8). With Occam mass $d\mu_{\mathrm{Sol}}\left(\omega \right)\propto 2^{-\ell (\omega )}$, $k_{\left(d,\mu_{\mathrm{Sol}}\right)}$ is the Solomonoff feature mixture of Parts IV–VI (Proposition 9).*
*3.*  *(Spectrum from the measure, under hypotheses). Under the orthogonality or near-orthogonality hypotheses of Theorem 2, the spectrum is controlled by the landmark weights. With equally normalized orthogonal features, uniform mass gives a flat nonzero spectrum; the corresponding near-orthogonality bounds control departures from flatness. Under the stated feature conditions, Occam mass gives $\lambda_{i}≍2^{-\ell_{i}}$ in the stated perturbative regime.*
*4.*  *(GW selects the shared metric–measure datum). In the metric–measure specialization, Solomonoff–Gromov selection calibrates the relational geometry d together with the candidate mass. Once a relational geometry has been selected or fixed, the Kolmogorov/Solomonoff distinction is represented by the downstream choice of landmark measure: empirical/uniform mass gives the KC projection, while Occam mass gives the Solomonoff projection.*

*Consequently, the Kolmogorov and Solomonoff kernels are not rival constructions but rather the empirical/uniform mass and Occam mass projections of the same algorithmic metric–measure template; d carries algorithmic similarity, while μ fixes the weighting convention. This is the geometry-first unification of the kernel constructions of Parts I–VI.*

**Proof.**  The feature formula shows directly that *d* determines the functions $z\mapsto e^{-\gamma d(z,\omega )}$, while μ supplies their integration weights. The two canonical choices of μ are exactly Propositions 8 and 9. The spectral statement is Theorem 2. The final statement is a reading of the metric–measure objective: GW compares relational distances through couplings of the candidate masses, while the master kernel separates the distance-defined features from the measure-defined weights. Proposition 12 gives the corresponding Hilbert-space formulation after kernelization.    □

**Figure 2:**
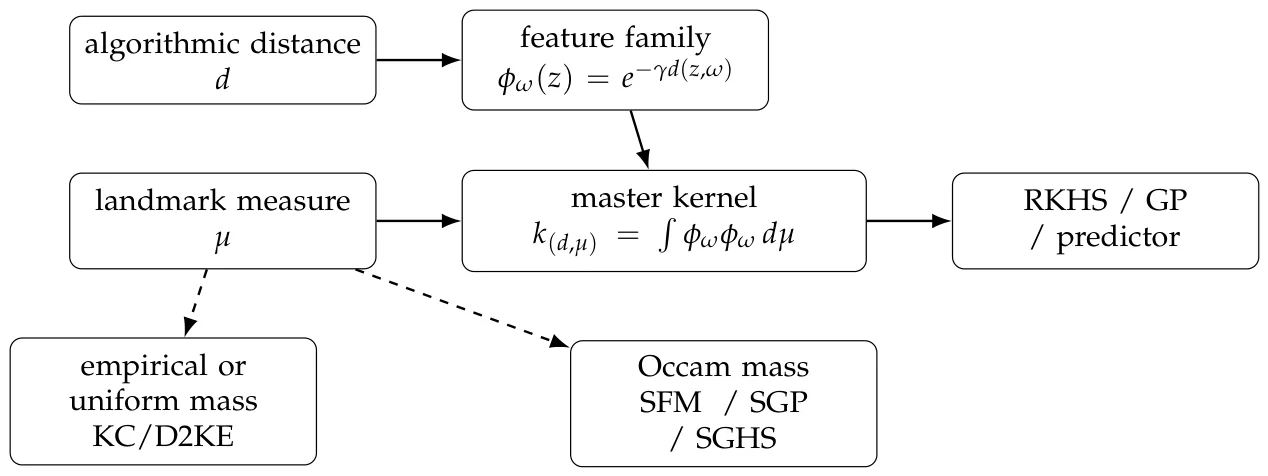
The metric–measure master kernel. The distance fixes the D2KE feature family, while the landmark measure supplies empirical/uniform or Occam weights.

Figure 2 depicts the master-kernel factorization and its two canonical projections.

The preceding proposition recovers the *feature-mixture* formula. A separate question is whether those feature weights become the *operator spectrum* $\lambda_{i}≍2^{-\ell_{i}}$. The answer requires a spectral hypothesis on the feature dictionary, since non-orthogonal features can move mass between eigenmodes. The following theorem records the exact and perturbative cases.

**Theorem 2** (Spectrum of the recovered Solomonoff kernel)**.**  *Let ${\left\{u_{\omega }\right\}}_{\omega \in \Omega }\subset L^{2}\left(X,\rho \right)$ be a feature dictionary indexed by a prefix-free* Ω, with weights $w_{\omega }>0$; set the weight-scaled features $v_{\omega }=w_{\omega }^{1/2}u_{\omega }$ and the operator $T=AA^{*}$, $\left(Ac\right)=\sum_{\omega }c_{\omega }v_{\omega }$. The nonzero eigenvalues of T coincide with those of the Gram matrix S, $S_{\omega \omega^{\prime }}={\langle v_{\omega },v_{\omega^{\prime }}\rangle }_{L^{2}\left(\rho \right)}$. Assume:

*(H1)*  * bounded features: $0\;<\;b\;\leq \;∥u_{\omega }∥\;\leq B<\infty$ for all ω;*
*(H2_δ_)*  *δ-near-orthogonality: for every ω,*
  $$\sum_{\omega^{\prime }\neq \omega }\left|\langle v_{\omega },v_{\omega^{\prime }}\rangle \right|\leq \delta \;\langle v_{\omega },v_{\omega }\rangle .$$

*Then, with $w_{\left(1\right)}\geq w_{\left(2\right)}\geq \cdots$ as the sorted weights and $\lambda_{1}\geq \lambda_{2}\geq \cdots$ as the sorted nonzero eigenvalues:*

*1.*  *(Exact case). If $\left\{v_{\omega }\right\}$ are orthogonal, define $a_{\omega }=w_{\omega }{∥u_{\omega }∥}^{2}$ and let $a_{\left[1\right]}\geq a_{\left[2\right]}\geq \cdots$ be these products in decreasing order. Then, $\lambda_{i}=a_{\left[i\right]}$. In particular, when $∥u_{\omega }∥\;\equiv \;1$, the product ordering is the weight ordering: uniform weights $w_{\omega }\equiv 1$ give $\lambda_{i}\equiv 1$ (the finite KC-HS spectrum is flat/degenerate), and Solomonoff weights $w_{\omega }=2^{-\ell (\omega )}$ give $\lambda_{i}=2^{-\ell_{i}}$ exactly.*
*2.*  *(Perturbation—finite global band). If* Ω *is finite, then under (H1) and (H2_δ_), every nonzero eigenvalue λ of T lies in*
  $$\left(1-\delta \right)\;b^{2}\;w_{\min}\;\leq \;\lambda \;\leq \;\left(1+\delta \right)\;B^{2}\;w_{\max},\;w_{\min}=\min_{\omega }w_{\omega },\;\;w_{\max}=\max_{\omega }w_{\omega }.$$
  *For Solomonoff weights, this reads $\left(1-\delta \right)\;b^{2}\;2^{-\ell_{\max}}\leq \lambda \leq \left(1+\delta \right)\;B^{2}\;2^{-\ell_{\min}}$. For countably infinite* Ω *with $\sum_{\omega }w_{\omega }<\infty$, there is generally no positive $w_{\min}$. The correct statement is local; every nonzero eigenvalue belongs to at least one interval*
  $$[\left(1-\delta \right)w_{\omega }∥u_{\omega }{∥}^{2},\left(1+\delta \right)w_{\omega }∥u_{\omega }{∥}^{2}],$$
  *and the only possible nonzero spectral points are discrete eigenvalues of finite multiplicity accumulating, if at all, at zero.*
*3.*  *(Perturbation—finite per-index band). If *Ω *is finite and the weight dynamic range is bounded as $w_{\max}\leq \kappa \;w_{\min}$ with κ≥1, then for every i,*
  $$\left(b^{2}-\delta \kappa B^{2}\right)\;w_{\left(i\right)}\;\leq \;\lambda_{i}\;\leq \;\left(B^{2}+\delta \kappa B^{2}\right)\;w_{\left(i\right)}.$$
  *In particular, for normalized Solomonoff weights $∥u_{\omega }∥\;\equiv \;1$, $w_{\omega }=2^{-\ell (\omega )}$, with δκ<1,*
  $$\left(1-\delta \kappa \right)\;2^{-\ell_{i}}\;\leq \;\lambda_{i}\;\leq \;\left(1+\delta \kappa \right)\;2^{-\ell_{i}},$$
  *so $\lambda_{i}≍2^{-\ell_{i}}$ per index.*

*Throughout, either* Ω *is finite or $\sum_{\omega }w_{\omega }<\infty$ (automatic for Solomonoff weights by Kraft) together with (H1), in which case T is trace class and its nonzero spectrum is discrete. In the infinite case, the Gershgorin localization applies to nonzero eigenvalues by the maximal-coordinate argument for $\ell^{2}$ eigenvectors. The spectral point zero may be an eigenvalue when the operator has a kernel, or it may lie in the continuous spectrum; no stronger classification is used here. For uniform weights on an infinite dictionary, the operator need not be compact, so the flat-spectrum statement is restricted to finite* Ω*.*

**Proof.**  The nonzero spectra of $AA^{*}$ and $A^{*}A=S$ coincide (standard), and *S* is real symmetric PSD with diagonal $S_{\omega \omega }=w_{\omega }{∥u_{\omega }∥}^{2}\in \left[b^{2}w_{\omega },B^{2}w_{\omega }\right]$ by (H1).

*(1) Exact case.* If $v_{\omega }$ are orthogonal, then $S=\mathrm{diag}(w_{\omega }∥u_{\omega }{∥}^{2})$, for which the eigenvalues are the sorted diagonal *products* $a_{\left[i\right]}$; only when the feature norms are constant does this sorting coincide with the weight order.

*(2) Global localization.* By Gershgorin’s circle theorem in the finite case, every nonzero eigenvalue λ satisfies $|\lambda -S_{\omega \omega }|\;\leq \sum_{\omega^{\prime }\neq \omega }\left|S_{\omega \omega^{\prime }}\right|\leq \delta S_{\omega \omega }$ for some index ω. Thus, $\lambda \in [\left(1-\delta \right)S_{\omega \omega },\left(1+\delta \right)S_{\omega \omega }]$. Taking the extrema gives the finite global band exactly as stated. For countably infinite trace-class *S*, a nonzero eigenvector has a coordinate of maximal absolute value (its coordinates tend to zero); applying the eigenvalue equation at that coordinate gives the same local interval. Because positive summable weights need not have a positive infimum, no uniform positive lower band is asserted in the infinite case.

*(3) Per-index band.* Split S=D+E with $D=\mathrm{diag}(S_{\omega \omega })$ and *E* the off-diagonal part (symmetric, zero diagonal). The eigenvalues of *D* are its diagonal entries; let $S_{\left(1\right)}\geq S_{\left(2\right)}\geq \cdots$ be their decreasing order statistics, so $\lambda_{i}\left(D\right)=S_{\left(i\right)}$. For every *i*, Weyl’s perturbation inequality for symmetric matrices gives

$$|\lambda_{i}-S_{\left(i\right)}{|\;\leq \;∥E∥}_{\mathrm{op}}\;\leq \;\max_{\omega }\sum_{\omega^{\prime }\neq \omega }\left|S_{\omega \omega^{\prime }}\right|\;\leq \;\delta \;\max_{\omega }S_{\omega \omega }\;=\;\delta \;S_{\left(1\right)}\;\leq \;\delta B^{2}w_{\max},$$

where ${∥E∥}_{\mathrm{op}}\leq \max_{\omega }\sum_{\omega^{\prime }\neq \omega }\left|S_{\omega \omega^{\prime }}\right|$ is the row-sum (Schur) bound for a symmetric matrix and the third inequality is (H2_*δ*_). Next, the pointwise bracketing $b^{2}w_{\omega }\leq S_{\omega \omega }\leq B^{2}w_{\omega }$ of (H1) passes to the order statistics: using the max–min representation $S_{\left(i\right)}=\max_{|T|=i}\min_{\omega \in T}S_{\omega \omega }$, which is monotone in each entry, and the fact that scaling by $b^{2},B^{2}>0$ preserves order,

$$b^{2}w_{\left(i\right)}\;\leq \;S_{\left(i\right)}\;\leq \;B^{2}w_{\left(i\right)}\;\left(\mathrm{all}\;i\right).$$

Combining the two displays with $w_{\max}\leq \kappa \;w_{\min}\leq \kappa \;w_{\left(i\right)}$,

$$\lambda_{i}\;\leq \;S_{\left(i\right)}+\delta B^{2}w_{\max}\;\leq \;B^{2}w_{\left(i\right)}+\delta \kappa B^{2}w_{\left(i\right)}=\left(B^{2}+\delta \kappa B^{2}\right)w_{\left(i\right)},$$

$$\lambda_{i}\;\geq \;S_{\left(i\right)}-\delta B^{2}w_{\max}\;\geq \;b^{2}w_{\left(i\right)}-\delta \kappa B^{2}w_{\left(i\right)}=\left(b^{2}-\delta \kappa B^{2}\right)w_{\left(i\right)},$$

which is the stated per-index band; the normalized Solomonoff specialization sets b=B=1. Without the dynamic-range control $w_{\max}\leq \kappa w_{\min}$, the additive error $\delta S_{\left(1\right)}$ need not be small relative to $w_{\left(i\right)}$ at large *i*; the global band of part 2 remains valid in the finite case, while the stated infinite-dimensional conclusion is local. There is also a sharper consequence of the row condition when δ<1: with D=diag(S),

$$|x^{⊤}\left(S-D\right)x|\leq \sum_{i}x_{i}^{2}\sum_{j\neq i}\left|S_{ij}\right|\leq \delta x^{⊤}Dx.$$

Hence (1−δ)D⪯S⪯(1+δ)D, and the min–max principle gives relative eigenvalue bounds without a dynamic-range factor. For a trace-class dictionary, extend this quadratic-form inequality from finitely supported vectors by continuity.    □

**Remark 6** (Scope and what the experiments show)**.**  *Two limitations concerning the informativeness of (H2_δ_) require attention. (i) Across length classes, the weight dynamic range can be enormous (w ranging over $2^{-1},\ldots ,2^{-L}$); since the radius/diagonal ratio for a deep feature against a shallow one scales like $2^{(\ell -\ell^{\prime })/2}\langle u_{\omega },u_{\omega^{\prime }}\rangle$, (H2_δ_) with small δ requires the cross-class coherence to decay with the length gap or a bounded effective dynamic range (multiplicity within length classes). When this fails, the Gershgorin band is loose, indeed vacuous, once δ≥1. (ii) The synthetic spectral-calibration study of Section 14 confirms the sharp statement, i.e., for the generalized synthetic orthogonal dictionary, the ratio $\lambda_{i}/2^{-\ell_{i}}$ is 1 to machine precision, and under increasing coherence it degrades gracefully, staying in [0.95,1.02] at coherence 0.05 and [0.75,1.02] at coherence 0.1, so that the true spectrum tracks $2^{-\ell_{i}}$ far more tightly than predicted by the worst-case Gershgorin envelope. Strictly positive exponential D2KE features at finite distances cannot be exactly orthogonal under a nonzero reference probability. The exact synthetic check therefore concerns a generalized feature dictionary. The theorem states that the correspondence is exact under orthogonality and robust under perturbation, with (H2_δ_) being the same near-orthogonality condition under which Part IV’s analogous result holds.*

**Remark 7** (What the distance and the measure each control)**.**  *Propositions 8 and 9 show that the two classical kernels differ only in the landmark measure: on the same GW-selected geometry, uniform μ yields the KC-kernel and $\mu \propto 2^{-\ell }$ (Occam) yields the Solomonoff kernel. The correct division of labor is that the distance d determines the raw feature dictionary $\left\{\phi_{\omega }\right\}$, the measure μ weights the feature covariance contributions, and the eigenvalues and eigenfunctions of the resulting operator are determined jointly by the weights and the feature Gram matrix. It is false in general that d fixes the eigenfunctions while μ fixes only the eigenvalues; for coherent (non-orthogonal) features, changing μ rotates the eigenvectors as well, as shown by an immediate two-feature counterexample. Only under explicit orthogonality of the features, simultaneous diagonalization, or a perturbation estimate with appropriate spectral gaps may one keep the basis fixed or approximately fixed. The row condition (H2_δ_) by itself controls eigenvalues, not individual eigenvectors:*

$$\underset{\mathrm{feature}\;\mathrm{dictionary}/\mathrm{relational}}{\underset{︸}{\mathrm{GW}\;\mathrm{selects}\;d}}\;\underset{\mathrm{spectrum}\;\mathrm{and},\;\mathrm{in}\;\mathrm{general},\;\mathrm{the}\;\mathrm{eigenbasis}}{\underset{︸}{\mathrm{Occam}\;\mathrm{reweighting}\;\mathrm{sets}\;\mu }}.$$

*GW model selection alone, being isometry-invariant, fixes only the relational layer; recovering the Solomonoff kernel (not merely a KC-kernel) requires the additional Occam choice of μ, and reading $2^{-\ell_{i}}$ as the eigenvalue law requires the conditioning hypotheses. Predictive (induction-level) fidelity additionally requires that the marking/labeling be matched, which is the role of the marked objective (10).*

### 11.3. Category-Specific Kernelization and Induction Functors

The selected space is not automatically a kernel, and a kernel is not automatically an induction rule. The full pipeline is

$$\begin{array}{c} \begin{array}{c} \mathrm{Solomonoff}\;\mathrm{object}⟶\mathrm{best}\;\mathrm{approximating}\;\mathrm{space}\\ ⟶\mathrm{kernel}/\mathrm{operator}/\mathrm{predictor}⟶\mathrm{induction}. \end{array} \end{array}$$

For a structured class C, let

$$A_{C}^{★}\in \underset{A\in C}{arg min}\left[D_{C}\left(S_{\mathrm{Sol}},A\right)+\lambda \mathrm{Code}\left(A\right)+\eta \;{\mathrm{PredLoss}}_{C}\left(A;D\right)\right].$$

To turn $A_{C}^{★}$ into a learning object, one must specify a representation or prediction functor

$$K_{C}:C⟶\left\{\mathrm{positive}\text{-}\mathrm{semidefinite}\;\mathrm{kernels},\;\mathrm{operators},\;\mathrm{regularizers},\;\mathrm{summaries},\;\mathrm{or}\;\mathrm{predictors}\right\}.$$

The Kolmogorov/Solomonoff distinction is often not a different geometry but a different weighting convention on the same family of features:

$$\begin{array}{c} \mathrm{Kolmogorov}\;\mathrm{projection}=\mathrm{algorithmic}\;\mathrm{features}\;\mathrm{plus}\;\mathrm{empirical}\;\mathrm{or}\;\mathrm{uniform}\;\mathrm{mass}, \end{array}$$

$$\begin{array}{c} \mathrm{Solomonoff}\;\mathrm{projection}=\mathrm{algorithmic}\;\mathrm{features}\;\mathrm{plus}\;\mathrm{Occam}\;\mathrm{mass}\;2^{-\mathrm{Code}}. \end{array}$$

For every feature sum or integral below, require measurable features and finite diagonal second moments, $\sum_{\omega }w_{\omega }{\left|\phi_{\omega }\left(x\right)\right|}^{2}<\infty$ for every input (or its integral analogue). Cauchy–Schwarz then ensures that cross terms converge. Kraft summability alone does not control unbounded features. Accordingly, a category may admit two related functors $K_{\mathrm{KC}}^{C}$ and $K_{\mathrm{Sol}}^{C}$. The following paragraphs state what those functors mean in each branch.

**Metric–measure spaces.** This is the developed case. For A=(Z,d,μ),

$$K_{C}\left(A\right)\left(z,z^{\prime }\right)=\int_{Z}e^{-\gamma d(z,\omega )}e^{-\gamma d(z^{\prime },\omega )}\;d\mu \left(\omega \right).$$

With algorithmic distance and empirical or uniform landmark mass, this is the KC/D2KE kernel. With the same features but Occam mass $d\mu_{\mathrm{Sol}}\left(\omega \right)\propto 2^{-\ell (\omega )}$, it is the Solomonoff feature mixture. The distance carries algorithmic similarity; the measure distinguishes empirical/Kolmogorov from Solomonoff weighting.

**Hilbert spaces.** If the selected approximation is already Hilbertian,

$$A=(Z\subset H,∥\cdot {∥}_{H},\mu ),$$

then the kernel may be the inner product $k_{H}\left(z,z^{\prime }\right)={\langle z,z^{\prime }\rangle }_{H}$. If only Hilbert distances are available, choose a base point $z_{0}$ and recover

$$k_{H}\left(z,z^{\prime }\right)=\frac{1}{2}\left[d_{H}{\left(z,z_{0}\right)}^{2}+d_{H}{\left(z^{\prime },z_{0}\right)}^{2}-d_{H}{\left(z,z^{\prime }\right)}^{2}\right].$$

When the embedding preserves NCD, CKC, AMI, or another algorithmic distance with empirical mass, this is a Kolmogorov-type Hilbert kernel. With posterior/Occam weighting, the natural Solomonoff version is a covariance kernel such as

$$k_{\mathrm{Sol},H}\left(x,y\right)={\mathrm{Cov}}_{h∼\pi_{D}}\left(\phi_{h}\left(x\right),\phi_{h}\left(y\right)\right),$$

which is a centered variant of the SFM construction. It equals the uncentered second-moment kernel only when the feature means vanish.

**Banach spaces.** A Banach space has a norm but no canonical inner product, so it need not yield an RKHS directly. Three routes remain natural. First, choose a measure ρ on dual functionals $\ell \in B^{*}$ and define

$$k_{B}\left(x,y\right)=\int_{B^{*}}\sigma \left(\ell \left(x\right)\right)\sigma \left(\ell \left(y\right)\right)\;d\rho \left(\ell \right).$$

An empirical dictionary produces the Kolmogorov projection, whereas $\rho \left(\ell \right)\propto 2^{-\mathrm{Code}(\ell )}$ yields

$$k_{\mathrm{Sol},B}\left(x,y\right)=\sum_{\ell }2^{-\mathrm{Code}(\ell )}\sigma \left(\ell \left(x\right)\right)\sigma \left(\ell \left(y\right)\right).$$

Second, one may retain an RKBS or duality pairing rather than force a Hilbert representation; this is appropriate for $L^{1}$, bounded-variation, Besov, Barron, and variation-norm geometries. Third, one may apply D2KE after Banach selection:

$$k_{B}\left(x,y\right)=\int_{B}e^{{-\gamma ∥x-\omega ∥}_{B}}e^{{-\gamma ∥y-\omega ∥}_{B}}\;d\mu \left(\omega \right),$$

which is positive semidefinite even when the Banach norm is not Hilbertian.

**Topological spaces.** A bare topology gives no kernel or predictor. It must be represented by features. Choose a filtration, compute persistent homology, and map each object to a persistence landscape [61], image [62], diagram feature, or related vector [63],

$$\Phi_{\mathrm{top}}\left(x\right)=\mathrm{persistence}\;\mathrm{feature}\;\mathrm{of}\;x.$$

Then, $k_{\mathrm{top}}\left(x,y\right)=\langle \Phi_{\mathrm{top}}\left(x\right),\Phi_{\mathrm{top}}\left(y\right)\rangle$ is a topological kernel. A Kolmogorov version uses filtrations induced by algorithmic distance, prefix depth, AMI, or compressor geometry. A Solomonoff version additionally weights classes, covers, branches, or filtration events by code:

$$k_{\mathrm{Sol},\mathrm{top}}\left(x,y\right)=\sum_{\eta \in T}2^{-\mathrm{Code}(\eta )}\Phi_{\eta }\left(x\right)\Phi_{\eta }\left(y\right).$$

**Graphs, trees, and ultrametrics.** Graphs and trees have native kernels: diffusion kernels $\left(e^{-tL}\right)\left(x,y\right)$ [64], reversible random-walk resolvents ${\left(I-\beta S\right)}^{-1}=\sum_{m\geq 0}\beta^{m}S^{m}$, where *S* is the symmetric realization of the transition operator and 0≤β<1, subtree kernels [65], and prefix kernels. For a prefix tree,

$$k_{\mathrm{pref}}\left(x,y\right)=\sum_{u⪯x,y}w\left(u\right).$$

Uniform or empirical w(u) gives a Kolmogorov/tree kernel; $w\left(u\right)=2^{-|u|}$, $2^{-K(u)}$, or a depth-summable modification of M(u) gives the tree-native Solomonoff kernel of Equation (16).

**Operator and spectral models.** If the selected object is a positive operator *T* with measurable eigenfunction representatives and $\sum_{i}\lambda_{i}{\left|e_{i}\left(x\right)\right|}^{2}<\infty$ at every input, its pointwise kernel is

$$k_{T}\left(x,y\right)=\sum_{i}\lambda_{i}e_{i}\left(x\right)e_{i}\left(y\right).$$

The Kolmogorov version lets the eigenvalues come from empirical geometry or learned covariance. The Solomonoff version requires the spectral calibration $\lambda_{i}\approx 2^{-\ell_{i}}$ developed in Section 16. This is the operator-level form of the same rule: empirical or geometry-derived weights give KC-type kernels, while description-length weights give Solomonoff kernels.

**Induction requires a predictive rule.** The category-specific functor supplies a kernel, operator, feature map, Banach/RKBS structure, topological summary, or graph diffusion. Induction begins only after one supplies a predictive mechanism: kernel ridge regression, a Gaussian-process prior, a Bayesian posterior over explanations, a Markov law, a regularized empirical risk problem, a context-tree rule, or a mixture over candidate spaces. Therefore, a complete inductive candidate is not merely a space *A* but a tuple

$$A=(A,D_{A},K_{A},P_{A},\mathrm{Code}\left(A\right)),$$

where $D_{A}$ is the native distortion, $K_{A}$ is the representation functor, and $P_{A}$ is the induced predictive law. The strongest Solomonoff-like construction is not a single selected object but a mixture over such candidates.

### 11.4. Category-Native Analogues of Solomonoff Gaussian Processes

The maturity map in Table 4 should be read constructively. The Solomonoff Gaussian process is the Gaussian/Hilbert realization of a more general rule: approximate the Solomonoff source, calibrate the representation by description length or Occam mass, and use the native predictor of the target category. This becomes Solomonoff-inspired TDA in topological spaces, Occam-weighted variation-norm learning in Banach spaces, algorithmic diffusion on graphs, prefix/context-tree prediction on trees, and a mixture over coded spaces at the universal level. These are projection rules rather than assertions of equivalence: each row indicates which data structure, comparison, representation, and predictor replace the Hilbert/Gaussian construction.

### 11.5. How Induction Looks in Each Approximation Category

Write

$$P_{C}\left(\cdot |D\right)={\mathrm{Ind}}_{C}\left(A_{C}^{★}\right),$$

where ${\mathrm{Ind}}_{C}$ is the native learning rule. Different categories preserve different projections of Solomonoff induction; none is the full universal semimeasure unless the category itself is the universal class of computable semimeasures; thus, this subsection provides a constructional dictionary. Universal dominance is available only at an appropriate mixture level, and a single Hilbert, Banach, topological, graph, spectral, or Gaussian candidate carries only the projection described below.

**Metric–measure induction.** For A=(Z,d,μ),

$$\left(Z,d,\mu \right)⟼k_{A}⟼H_{k_{A}}⟼\mathrm{KRR},\;\mathrm{GP},\;\mathrm{or}\;\mathrm{Bayesian}\;\mathrm{prediction}.$$

With the D2KE kernel, one may perform kernel ridge regression,

$$\hat{f}=\underset{f\in H_{k_{A}}}{arg min}\left[\sum_{i=1}^{n}\ell \left(f\left(z_{i}\right),y_{i}\right)+\rho {∥f∥}_{H_{k_{A}}}^{2}\right],$$

or Gaussian-process prediction, $f∼\mathrm{GP}(0,k_{A})$. The Kolmogorov version uses algorithmic/compression distance with empirical mass. The Solomonoff version keeps the same geometry but uses $\mu \left(\omega \right)\propto 2^{-\ell (\omega )}$, yielding the SFM/SKCO/SGP route.

**Metric induction without a native measure.** For A=(Z,d), one must add empirical, diffusion, or Occam weights before obtaining a probabilistic model. When $d^{2}$ is conditionally negative definite, one can use $k_{d}\left(z,z^{\prime }\right)=\exp\left(-\gamma d{\left(z,z^{\prime }\right)}^{2}\right)$; otherwise, a finite-landmark D2KE construction remains available. A purely metric alternative is the algorithmic Nadaraya–Watson rule

$$\hat{y}\left(x\right)=\frac{\sum_{i}w_{i}e^{-\gamma d(x,x_{i})}y_{i}}{\sum_{i}w_{i}e^{-\gamma d(x,x_{i})}}.$$

The Kolmogorov version uses NCD/CKC/AMI distance and empirical weights; the Solomonoff version uses $w_{i}\propto 2^{-K(x_{i})}$ or explanation weights $w_{h}\propto 2^{-\ell (h)}$. Metric induction approximates Solomonoff by proximity in algorithmic distance.

**Hilbert induction.** For Z⊂H, induction is ordinary Hilbert space learning:

$$\hat{f}=\underset{f\in H}{arg min}\left[\sum_{i}\ell \left({\langle f,z_{i}\rangle }_{H},y_{i}\right)+\rho {∥f∥}_{H}^{2}\right].$$

Equivalently, after kernelization, one uses RKHS regression or a Gaussian expansion:

$$f=\sum_{i}\sqrt{\lambda_{i}}\;\xi_{i}e_{i},\;\xi_{i}∼N\left(0,1\right).$$

The Hilbert-valued Gaussian expansion requires $\sum_{i}\lambda_{i}<\infty$; pointwise GP evaluation also requires the diagonal convergence stated above. The Solomonoff-calibrated version imposes $\lambda_{i}≍2^{-\ell_{i}}$ and $k_{\mathrm{Sol}}\left(x,y\right)=\sum_{i}2^{-\ell_{i}}e_{i}\left(x\right)e_{i}\left(y\right)$. This is a second-order covariance approximation, useful for regression and uncertainty quantification but lossy relative to the discrete universal mixture.

**Banach induction.** For $A=(B,∥\cdot {∥}_{B},\mu_{B})$, prediction is typically the regularized empirical risk

$$\hat{f}=\underset{f\in B}{arg min}\left[\sum_{i}\ell \left(f\left(x_{i}\right),y_{i}\right)+\rho {∥f∥}_{B}^{p}\right],\;p\geq 1.$$

This formula requires bounded evaluation functionals on the chosen function space. For $L^{1}$ or general BV equivalence classes, replace $f(x_{i})$ by specified bounded observation functionals; point values are not intrinsically defined. Existence of a minimizer additionally requires the usual coercivity and lower-semicontinuity/compactness hypotheses. A Solomonoff–Banach construction uses the Occam-weighted atomic gauge

$${∥f∥}_{\mathrm{Sol}\text{-}\mathrm{Var}}=\inf\left\{\sum_{\omega }2^{\ell (\omega )}\left|a_{\omega }\right|:f=\sum_{\omega }a_{\omega }\phi_{\omega }\right\}.$$

The series is required to converge in the declared ambient space. This gauge is a norm on its finite-valued span only under separation/nondegeneracy hypotheses; no norm claim is made without them. Banach induction thus approximates Solomonoff by sparse or variation-norm aggregation of simple explanations rather than by Gaussian smoothing.

**Topological induction.** For A=(Z,τ), prediction passes through summaries

(Z,τ)⟼filtration⟼persistentfeatures⟼predictor.

An algorithmic-distance filtration can be mapped to a persistence feature $\Phi_{\mathrm{top}}\left(x\right)$, after which $k_{\mathrm{top}}\left(x,y\right)=\langle \Phi_{\mathrm{top}}\left(x\right),\Phi_{\mathrm{top}}\left(y\right)\rangle$ supports KRR or classification; statistical foundations for such diagram-valued features are available [66,67]. The Solomonoff-topological version weights persistent classes, births, deaths, branches, covers, or filtration events by $2^{-\mathrm{Code}}$. It preserves multiscale shape and connectivity rather than all pairwise distances.

**Graph induction.** For A=(G=(V,E),w,μ), use graph diffusion, Laplacian regularization [68,69], graph kernels, or random walks. A semi-supervised learner can solve

$$\hat{f}=\underset{f:V\to R}{arg min}\left[\sum_{i\in L}\ell \left(f\left(i\right),y_{i}\right)+\rho f^{⊤}L_{G}f\right]$$

or use the diffusion kernel $k_{t}=e^{-tL_{G}}$ [70]. A Kolmogorov graph has edge weights $w_{ij}=e^{-\gamma d_{K}\left(x_{i},x_{j}\right)}$. A Solomonoff graph may use node masses $\mu_{i}\propto 2^{-K(x_{i})}$ or joint-explanation weights

$$w_{ij}=\sum_{\omega :\;\omega \;\mathrm{explains}\;i,j}2^{-\ell (\omega )}.$$

Graph induction approximates Solomonoff by diffusion over algorithmic-similarity networks.

**Tree and ultrametric induction.** For $A=(T,d_{\mathrm{tree}},\mu_{T})$, especially a prefix tree, induction is naturally context-tree or prefix-mixture prediction [71,72],

$$P\left(a|x_{<t}\right)=\sum_{u⪯x_{<t}}w\left(u|x_{<t}\right)P_{u}\left(a|u\right).$$

The prefix kernel is $k_{\mathrm{pref}}\left(x,y\right)=\sum_{u⪯x,y}w\left(u\right)$. Empirical w(u) gives a Kolmogorov tree kernel, while $w\left(u\right)=2^{-|u|}$, $2^{-K(u)}$, or a depth-summable modification of M(u) gives a Solomonoff prefix kernel. Tree induction preserves shared short explanatory prefixes.

**Operator and spectral induction.** For a positive operator *T* satisfying the pointwise diagonal-convergence hypotheses above, use

$$k_{T}\left(x,y\right)=\sum_{i}\lambda_{i}e_{i}\left(x\right)e_{i}\left(y\right)$$

and perform spectral KRR or GP prediction. The regularizer is

$${∥f∥}_{H_{T}}^{2}=\sum_{j}\frac{{\langle f,e_{j}\rangle }^{2}}{\lambda_{j}}.$$

Under Solomonoff calibration $\lambda_{j}≍2^{-\ell_{j}}$, complex modes are penalized exponentially. Spectral induction replaces universal mixture weights with operator regularization weighted by description length.

**Predictive-space induction.** If the category already consists of conditional laws $P_{A}\left(\cdot |x_{<t}\right)$, then the comparison is KL, Hellinger, deficiency, or regret, and induction is direct. The closest computable analogue of Solomonoff’s form is

$$P_{\mathrm{mix}}\left(a|x_{<t}\right)=\sum_{\theta }q\left(\theta |x_{<t}\right)P_{\theta }\left(a|x_{<t}\right),\;q\left(\theta |D\right)\propto 2^{-\mathrm{Code}(\theta )}P_{\theta }\left(D\right).$$

It is the algorithmic-prior counterpart of aggregating strategies and prediction with expert advice [73,74], and it preserves the mixture principle itself rather than only a geometry, covariance, or regularizer.

**Gaussian and SGP induction.** For $A=(H,\gamma_{C})$, where *C* is positive trace class, $Ce_{i}=\lambda_{i}e_{i}$, and the pointwise kernel below has finite diagonal, induction is GP regression [75] with covariance

$$k_{C}\left(x,y\right)=\sum_{i}\lambda_{i}e_{i}\left(x\right)e_{i}\left(y\right).$$

The posterior mean has the usual form $\hat{f}\left(x\right)=k_{x}^{⊤}{\left(K+\sigma^{2}I\right)}^{-1}y$. The Solomonoff-calibrated SGP takes $\lambda_{i}≍2^{-\ell_{i}}$, producing $k_{\mathrm{SGP}}\left(x,y\right)=\sum_{i}2^{-\ell_{i}}e_{i}\left(x\right)e_{i}\left(y\right)$. This retains Solomonoff-weighted covariance structure but not the higher-order structure of the universal mixture.

**Proposition 10** (SGP posterior mean, KRR, and representer form)**.**  *Let $k_{\mathrm{SGP}}$ be a fixed positive-semidefinite covariance kernel with RKHS $H_{\mathrm{SGP}}$, and let*

$$f∼\mathrm{GP}\left(0,k_{\mathrm{SGP}}\right),\;y_{i}=f\left(z_{i}\right)+\varepsilon_{i},\;\varepsilon_{i}\overset{\mathrm{iid}}{∼}N\left(0,\sigma^{2}\right),\;\sigma^{2}>0.$$

*Write $K_{ij}=k_{\mathrm{SGP}}\left(z_{i},z_{j}\right)$ and $k_{Z}\left(z\right)={\left(k_{\mathrm{SGP}}\left(z_{1},z\right),\ldots ,k_{\mathrm{SGP}}\left(z_{N},z\right)\right)}^{⊤}$. Then the posterior mean is*

(13) $$m_{N}\left(z\right)=E\left[f\left(z\right)|y\right]=k_{Z}{\left(z\right)}^{⊤}{\left(K+\sigma^{2}I\right)}^{-1}y=\sum_{i=1}^{N}\alpha_{i}k_{\mathrm{SGP}}\left(z_{i},z\right),\;\alpha ={\left(K+\sigma^{2}I\right)}^{-1}y.$$

*The same function is the unique minimizer*

(14) $$m_{N}=\underset{g\in H_{\mathrm{SGP}}}{arg min}\left\{\frac{1}{N}\sum_{i=1}^{N}{\left(y_{i}-g\left(z_{i}\right)\right)}^{2}+\frac{\sigma^{2}}{N}{∥g∥}_{H_{\mathrm{SGP}}}^{2}\right\}.$$

*If a reference measure provides the Mercer identification of the RKHS with the range of the covariance square root (quotienting any zero-observation directions), and the operator has eigenpairs $\left(e_{r},\lambda_{r}\right)$ with $\lambda_{r}>0$, then for $g=\sum_{r}a_{r}e_{r}$,*

$${∥g∥}_{H_{\mathrm{SGP}}}^{2}=\sum_{r}\frac{a_{r}^{2}}{\lambda_{r}}.$$

*Consequently, under Solomonoff calibration $\lambda_{r}≍2^{-\ell_{r}}$,*

$${∥g∥}_{H_{\mathrm{SGP}}}^{2}≍\sum_{r}2^{\ell_{r}}a_{r}^{2}:$$

*Occam weights in the SGP covariance become inverse-Occam penalties in the SGHS norm.*

**Proof.**  The joint Gaussian law of $\left(f\left(z_{1}\right),\ldots ,f\left(z_{N}\right),f\left(z\right)\right)$, together with the independent observation noise, gives the first two equalities in (13) by Gaussian conditioning. For the variational statement, decompose any $g\in H_{\mathrm{SGP}}$ as $g=g_{\|}+g_{⊥}$, where $g_{\|}$ belongs to the span of the kernel sections $k_{\mathrm{SGP}}\left(z_{i},\cdot \right)$. The reproducing property gives $g_{⊥}\left(z_{i}\right)=0$ for every *i*, while ${∥g∥}^{2}=∥g_{\|}{∥}^{2}+{∥g_{⊥}∥}^{2}$. Hence the minimizer of (14) has $g_{⊥}=0$. Substitution of $g=\sum_{i}\alpha_{i}k_{\mathrm{SGP}}\left(z_{i},\cdot \right)$ and the normal equations give $(K+\sigma^{2}I)\alpha =y$, which is exactly the conditional-mean coefficient vector. The spectral norm identity is the standard Mercer-coordinate description of the RKHS and yields the final equivalence when $\lambda_{r}≍2^{-\ell_{r}}$.    □

**Remark 8** (Where computability enters)**.**  *The posterior mean and the representer theorem are two descriptions of the same estimator, not two successive lossy projections. Finiteness follows from the N observations once the kernel is fixed. Computability additionally requires that $k_{\mathrm{SGP}}$ be supplied by a declared finite or effectively computable code, dictionary, or truncation. Exact Kolmogorov weights, an unrestricted universal mixture, or an untruncated exact Solomonoff covariance are not made computable merely by Gaussian conditioning. For a fixed computable kernel and Gaussian likelihood, however, prediction reduces to the finite linear system in* (13)*.*

**Mixture over coded spaces.** The strongest category-native construction is not to select one category or one space. Let $A={⋃}_{C}C$ be a countable coded collection of trees, graphs, Hilbert spaces, Banach models, kernels, topological models, and predictive laws, each carrying $P_{A}$. Programmatically,

$$P_{\mathrm{SpaceMix}}\left(\cdot |D\right)=\sum_{A\in A}q_{\beta }\left(A|D\right)P_{A}\left(\cdot |D\right),$$

with an energy combining native distortion, description length, and evidence. The metric–measure specialization is proved in Section 12. A cross-category mixture requires a fixed data-independent distortion calibration or another sequentially valid weighting rule; it is not obtained by substituting arbitrary empirical scores into the proved redundancy bound. Table 7 summarizes the per-category induction dictionary.

**Remark 9** (What each approximation preserves)**.**  *Tree induction preserves prefix/program hierarchy; metric induction preserves algorithmic similarity; metric–measure induction preserves similarity plus Occam mass; Hilbert and Gaussian induction preserve second-order covariance geometry; Banach induction preserves sparse or variation structure; topological induction preserves multiscale shape; graph induction preserves diffusion and adjacency structure; spectral induction preserves complexity decay; predictive induction preserves conditional laws; and mixture-over-spaces induction preserves the Solomonoff mixture principle itself.*

### 11.6. The Parent Object: A Schoenberg Metric–Measure Space

A basepointed Hilbertian metric and a reference measure determine a centered kernel, its RKHS, and its covariance operator. Applied to a kernel metric, this construction recovers a basepoint-centered version of the original kernel. Exact recovery of an arbitrary original kernel requires its feature-origin data as well.

**Definition 7** (Schoenberg metric–measure space)**.**  *A triple (X,d,ρ) is a Schoenberg mm-space if (X,d) is a complete separable metric space, ρ is a Borel probability measure on X, and d is of negative type, i.e., $-d^{2}$ is conditionally positive definite; equivalently, $e^{-\gamma d^{2}}$ is positive semidefinite for every γ>0 (Schoenberg); equivalently again, d is the kernel metric $d_{k}\left(x,y\right)=\sqrt{k(x,x)+k(y,y)-2k(x,y)}$ of some PSD kernel k (for the canonical algorithmic distance, this gate is known to fail; [76] states exactly this Schoenberg equivalence (his Lemma 32 (iii): “d is Euclidean iff d is a metric and $d^{2}$ is CND”) and proves $d_{K}$ does not satisfy it (Thm. 38); D2KE in Section 11.2 supplies one valid PSD representation, although it is not the only possible kernelization and need not preserve the original distances).*

**Proposition 11** (The functor F from a basepointed Schoenberg mm-space to RKHS, operator, and Gaussian)**.**  *Let (X,d) be separable with d of negative type ($d^{2}$ conditionally negative definite), let $x_{0}\in X$ be a basepoint, let ρ be a Borel probability measure on X with $d\left(\cdot ,x_{0}\right)\in L^{2}\left(\rho \right)$ and $d{\left(\cdot ,x_{0}\right)}^{2}$ measurable, and define the basepointed kernel*

$$k_{x_{0}}\left(x,y\right)\;=\;\frac{1}{2}\left[d{\left(x,x_{0}\right)}^{2}+d{\left(y,x_{0}\right)}^{2}-d{\left(x,y\right)}^{2}\right].$$

*Then:*

*1.*  *$k_{x_{0}}$ is positive semidefinite with $d_{k_{x_{0}}}=d$ and $k_{x_{0}}\left(x_{0},\cdot \right)\equiv 0$ (Schoenberg); different basepoints give different kernels with the same kernel metric, and the covariance origin depends on $x_{0}$.*
*2.*  *The RKHS $H_{k_{x_{0}}}$ is determined by d and $x_{0}$; as a function space modulo constants, it is basepoint-independent.*
*3.*  *Since $\int_{X}k_{x_{0}}\left(x,x\right)\;d\rho \left(x\right)=\int d{\left(x,x_{0}\right)}^{2}\;d\rho \left(x\right)<\infty$ by hypothesis, the integral operator $T_{k_{x_{0}}}f=\int_{X}k_{x_{0}}\left(\cdot ,y\right)f\left(y\right)\;d\rho \left(y\right)$ on $L^{2}\left(X,\rho \right)$ is self-adjoint, positive, and trace-class, with $\mathrm{tr}T_{k_{x_{0}}}=\int d{\left(\cdot ,x_{0}\right)}^{2}\;d\rho$; without this integrability hypothesis, an arbitrary Borel measure need* not *yield a trace-class operator.*
*4.*  The centered Gaussian measure $N(0,T_{k_{x_{0}}})$ on $L^{2}\left(\rho \right)$ is well defined, and its Cameron–Martin space is $\mathrm{Ran}(T_{k_{x_{0}}}^{1/2})$ with the range norm. If $I:H_{k_{x_{0}}}\to L^{2}\left(\rho \right)$ is the canonical inclusion, then $T_{k_{x_{0}}}=II^{*}$, and this Cameron–Martin space is isometrically the image of $H_{k_{x_{0}}}/\ker I$ under I, completed in the induced quotient/range norm.

*The frequently quoted normalization $k=1-\frac{1}{2}d^{2}$ is* not *generic; it is the special case of a constant-norm (spherical) embedding ∥Φ(x)∥≡1 and may be used only when that normalization is separately justified.*

**Proof.**  (1) By Schoenberg’s theorem, negative type of *d* is equivalent to the existence of a Hilbert embedding $\Phi_{0}:X\to H$ with $∥\Phi_{0}\left(x\right)-\Phi_{0}\left(y\right)∥=d\left(x,y\right)$. Center at the basepoint: $\Phi \left(x\right)=\Phi_{0}\left(x\right)-\Phi_{0}\left(x_{0}\right)$. Then, $\langle \Phi \left(x\right),\Phi \left(y\right)\rangle =\frac{1}{2}{[∥\Phi \left(x\right)∥}^{2}+{∥\Phi \left(y\right)∥}^{2}{-∥\Phi \left(x\right)-\Phi \left(y\right)∥}^{2}]=k_{x_{0}}\left(x,y\right)$, which is a Gram matrix; hence, PSD and $d_{k_{x_{0}}}{\left(x,y\right)}^{2}=k_{x_{0}}\left(x,x\right)+k_{x_{0}}\left(y,y\right)-2k_{x_{0}}\left(x,y\right)={∥\Phi \left(x\right)-\Phi \left(y\right)∥}^{2}=d{\left(x,y\right)}^{2}$. Changing $x_{0}$ translates Φ, which changes $k_{x_{0}}$ but not $d_{k_{x_{0}}}$. (2) is Moore–Aronszajn plus the observation that re-basing changes functions by additive constants. (3) $k_{x_{0}}$ is a measurable PSD kernel with $\int k_{x_{0}}\left(x,x\right)\;d\rho <\infty$; therefore, the associated integral operator is positive self-adjoint with $\mathrm{tr}T_{k_{x_{0}}}=\int k_{x_{0}}\left(x,x\right)\;d\rho \left(x\right)<\infty$ (monotone convergence over an orthonormal basis), and hence trace class. (4) A positive trace-class covariance on a separable Hilbert space determines a centered Gaussian measure; its Cameron–Martin space is $\mathrm{Ran}(T^{1/2})$ with ${∥h∥}_{CM}={∥T^{-1/2}h∥}_{L^{2}\left(\rho \right)}$. The inclusion *I* is Hilbert–Schmidt under the diagonal-integrability hypothesis and satisfies $T=II^{*}$. Polar decomposition identifies $\mathrm{Ran}(T^{1/2})$, with its range norm, with the included RKHS after quotienting by kerI and completing.    □

**Proposition 12** (Basepoint-centered KC and Solomonoff instances)**.**  *For $k\in \{k_{\mathrm{KC}},k_{\mathrm{Sol}}\}$, retain a common reference probability ρ and choose a basepoint $x_{0}$. Quotient any zero-distance pairs and impose the integrability hypotheses of Proposition 11. Then the construction $F(X,d_{k},x_{0},\rho )$ produces the kernel*

$$k^{x_{0}}\left(x,y\right)=k\left(x,y\right)-k\left(x,x_{0}\right)-k\left(x_{0},y\right)+k\left(x_{0},x_{0}\right)$$

*and its RKHS, integral operator, and centered Gaussian measure.*

**Proof.**  Substitute $d_{k}{\left(x,y\right)}^{2}=k\left(x,x\right)+k\left(y,y\right)-2k\left(x,y\right)$ into the basepoint formula. The remaining conclusions follow from Proposition 11.    □

**Remark 10** (Kernel origin, spectrum, and Gaussian support)**.**  *The metric and basepoint determine $k^{x_{0}}$, not an arbitrary original k. For example, k and k+c, c>0, have the same kernel metric and can have different RKHS dimensions. The reference measure remains necessary for the integral operator and Gaussian measure. Occam feature weights yield the stated eigenvalue calibration only under the conditioning hypotheses of Theorem 2. The Cameron–Martin space has Gaussian measure zero for infinite-rank covariance and measure one for finite-rank covariance. A PSD kernelization such as D2KE gives a Hilbertian kernel pseudometric; its zero-distance quotient is a metric space.*

**Corollary 1** (Retained feature maps and exact kernels)**.**  *Suppose a feature map Φ:X→H into a real Hilbert space is retained. Then $k\left(x,y\right)={\langle \Phi \left(x\right),\Phi \left(y\right)\rangle }_{H}$ is PSD and determines $H_{k}$. Its kernel pseudometric is $d_{k}\left(x,y\right)={∥\Phi \left(x\right)-\Phi \left(y\right)∥}_{H}$. Applying Proposition 11 to the metric quotient and a basepoint gives the translated-feature kernel ${\langle \Phi \left(x\right)-\Phi \left(x_{0}\right),\Phi \left(y\right)-\Phi \left(x_{0}\right)\rangle }_{H}$. Thus KC-HS and SGHS are recovered exactly from their respective retained kernels or feature maps; their metrics alone recover only the basepoint-centered constructions. If the feature norms are separately known to equal one, then $k=1-\frac{1}{2}d_{k}^{2}$.*

**Proof.**  The PSD property is the Gram identity; expanding the squared difference proves the metric formula and the final normalization.    □

**Remark 11** (Kernelization and the scope of algorithmic obstructions)**.**  *The unnormalized complexity metric studied in [76] is not Hilbertian, and that work proves a non-Euclidean power obstruction for exponents greater than 0.3. Its extension to normalized information or compression distances is not established there. A finite surrogate must therefore be checked separately for metricity and for conditional negative definiteness of its squared distances. D2KE supplies a PSD kernel for any measurable nonnegative dissimilarity and finite landmark measure, but it can change distances. It is one kernelization option, not the unique route to a Hilbert representation. A specified kernel determines its RKHS; its eigenfunctions also depend on the reference measure, and its Solomonoff spectral calibration still requires the feature assumptions of Theorem 2.*

### 11.7. Geometry-First Versus Kernel-First

**Figure 3:**
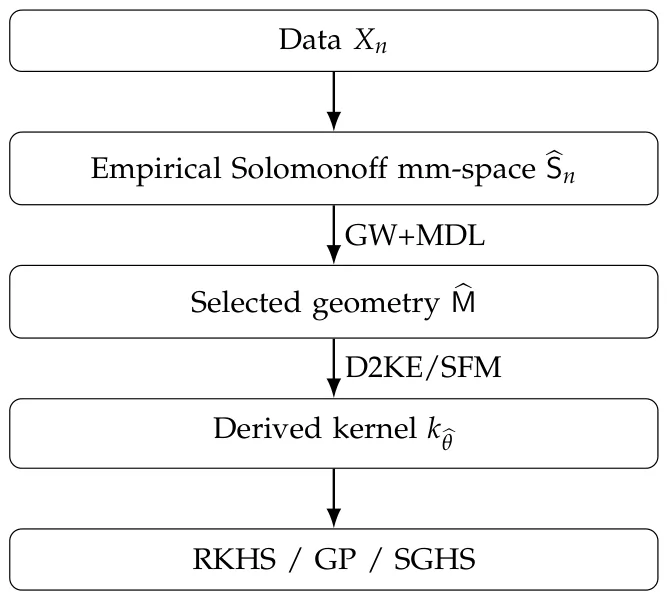
The implemented metric–measure pipeline from observations to an induced kernel predictor.

The kernel-first route, including Parts I–VI, searches over $k_{\theta }$ (equivalently, over its RKHS $H_{k_{\theta }}$) and reads off $d_{k_{\theta }}$ and $T_{k_{\theta }}$. The geometry-first route proposed here searches over $\left(Z_{\theta },d_{\theta },\mu_{\theta }\right)$ and *then* obtains $k_{\theta }$, $H_{k_{\theta }}$, and $T_{k_{\theta }}$. Figure 3 contrasts the two routes.

This ordering is the main conceptual contribution of the paper.

### 11.8. Stability of Kernelization

The next result states that if a candidate geometry is close to the empirical Solomonoff geometry in GW, then the induced D2KE kernels are close after transport by the optimal coupling.

**Proposition 13** (GW-to-kernel stability, finite version)**.**  *Let $M=(X,d_{X},\mu_{X})$ and $N=(Y,d_{Y},\mu_{Y})$ be finite mm-spaces with diameters bounded by D (the diameter bound is not needed for the constant below; it is retained for the transfer statements that reuse this setting). Let $k_{X},k_{Y}$ be their D2KE kernels with the same γ>0. For any coupling $\pi \in \Pi (\mu_{X},\mu_{Y})$, define the transported kernel discrepancy*

$$\Delta_{\pi }^{2}=\int {\left|k_{X}\left(x,x^{\prime }\right)-k_{Y}\left(y,y^{\prime }\right)\right|}^{2}\;d\pi \left(x,y\right)\;d\pi \left(x^{\prime },y^{\prime }\right).$$

*Then, there exists a constant $C_{\gamma ,D}$ such that*

$$\Delta_{\pi }\leq C_{\gamma ,D}{\left(\int |d_{X}\left(x,x^{\prime }\right)-d_{Y}\left(y,y^{\prime }\right){|}^{2}\;d\pi \left(x,y\right)\;d\pi \left(x^{\prime },y^{\prime }\right)\right)}^{1/2}.$$

*In particular, for an optimal GW coupling and the normalization of Definition 2,*

$$\Delta_{\pi^{★}}\leq \sqrt{2}\;C_{\gamma ,D}\;d_{\mathrm{GW},2}\left(M,N\right).$$

**Proof.**  Write the D2KE features $\psi_{\omega }^{X}\left(x\right)=e^{-\gamma d_{X}\left(x,\omega \right)}\in \left[0,1\right]$ so that

$$k_{X}\left(x,x^{\prime }\right)=\int \psi_{\omega }^{X}\left(x\right)\psi_{\omega }^{X}\left(x^{\prime }\right)\;d\mu_{X}\left(\omega \right),$$

and likewise for *Y*. Couple the landmark integration variable by the same π. For fixed coupled landmark pair (ω,ϖ), use $r\mapsto e^{-\gamma r}$ being γ-Lipschitz and all factors lying in [0,1]:

$$\left|\psi_{\omega }^{X}\left(x\right)\psi_{\omega }^{X}\left(x^{\prime }\right)-\psi_{\varpi }^{Y}\left(y\right)\psi_{\varpi }^{Y}\left(y^{\prime }\right)|\leq |\psi_{\omega }^{X}\left(x\right)-\psi_{\varpi }^{Y}\left(y\right)|+|\psi_{\omega }^{X}\left(x^{\prime }\right)-\psi_{\varpi }^{Y}\left(y^{\prime }\right)\right|$$

since |ab−cd|≤|a||b−d|+|d||a−c|≤|b−d|+|a−c| when |a|,|d|≤1. Each term is $\leq \gamma |d_{X}\left(x,\omega \right)-d_{Y}\left(y,\varpi \right)|$ (resp. with $x^{\prime },y^{\prime },\varpi$). Integrating the kernels’ difference against π⊗π on the points and using the same coupling to compare landmarks then applying Cauchy–Schwarz over the pair-coupling gives

$$\Delta_{\pi }\leq 2\gamma {\left(\;\int \int \;|d_{X}\left(x,x^{\prime }\right)-d_{Y}\left(y,y^{\prime }\right){|}^{2}\;d\pi \left(x,y\right)\;d\pi \left(x^{\prime },y^{\prime }\right)\right)}^{1/2}.$$

With the convention $d_{\mathrm{GW},2}^{2}=\frac{1}{2}\inf_{\pi }\;\int \int \;{\left|d_{X}-d_{Y}\right|}^{2}\;d\pi \;d\pi$, an optimal coupling yields

$$\Delta_{\pi^{★}}\leq 2\sqrt{2}\;\gamma \;d_{\mathrm{GW},2}\left(M,N\right).$$

Thus, one may take $C_{\gamma ,D}=2\gamma$ in the integral-discrepancy display and $2\sqrt{2}\gamma$ in the final GW-normalized display; diameter-dependent variants only change this constant.    □

**Remark 12.**
  *The constant $2\sqrt{2}\gamma$ is rigorous but pessimistic. The finite GW-to-kernel stability study of Section 14 measures $\Delta_{\pi^{★}}/d_{\mathrm{GW},2}\leq 0.69$ over random mm-space pairs at γ=1, inside the bound. This experiment is only a small finite sanity check, not a statistical estimate of a worst-case constant.*

**Remark 13.**
  *This proposition is the formal bridge from geometry selection to kernel approximation: small Solomonoff–Gromov distortion implies small discrepancy between the resulting transported kernels. It is the kernel-level analogue of saying that the candidate geometry preserves the empirical algorithmic relational structure.*

## 12. Mixtures over Coded Metric–Measure Models

The selector above returns one metric–measure geometry. A mixture retains uncertainty over a finite or countable coded family of mm-models, each carrying an induced predictor. This section proves the mixture result within that family. A mixture spanning the other rows of Table 4 is a programmatic extension of Equation (1), not a theorem that is claimed here.

The estimator (5) returns a *single* geometry $\hat{\theta }$, and hence a single RKHS/GP. With a genuine prior code, it is a MAP choice; with the experiment’s BIC/MDL score, it is a penalized-model-selection analogue. By contrast, Solomonoff induction is not a single model; it is the Bayesian mixture

$$M\left(x\right)\;=\;\sum_{\nu }2^{-K(\nu )}\;\nu \left(x\right)$$

over lower-semicomputable semimeasures with a universal description weighting. This universal-mixture representation is understood up to multiplicative constants relative to a universal monotone semimeasure. Selecting one geometry replaces a mixture by its mode, a *second* collapse on top of Gaussianization. The level of abstraction that moves closer to *M* than the RKHS route is to keep the mixture over spaces. Independently of this Solomonoff mixture argument, one might ask whether a single kernel *over geometries*, $e^{-\gamma d_{\mathrm{GW}}^{2}}$, would already suffice. On the candidate families of Section 14, this Gram is positive semidefinite to solver tolerance across instances, so a kernel over geometries is empirically admissible; the mixture is preferred for the reason given here, not because the Schoenberg gate fails one level up.

### 12.1. The Universal-Form Space-Mixture Posterior

**Definition 8** (Coded metric–measure model mixture)**.**  *Let A be a countable coded collection of complete metric–measure candidates*

$$A=(M_{A},D_{A},K_{A},P_{A},\mathrm{Code}\left(A\right)),$$

*where $D_{A}=d_{\mathrm{GW},p}^{p}\left(S_{\mathrm{Sol}},M_{A}\right)$ is a population-level mm-space distortion fixed independently of the realized prediction sequence. The map $K_{A}$ kernelizes $M_{A}$, the law $P_{A}\left(\cdot |D\right)$ is its induced predictor, and $L_{A}\left(D\right)$ is its evidence or likelihood on the observed data D. For an inverse temperature β≥0 satisfying $0<Z_{\beta }\left(D\right):=\sum_{A}e^{-\beta E_{A}\left(D\right)}<\infty$, define*

$$\begin{array}{cc} E_{A}\left(D\right) & =D_{A}+\lambda \mathrm{Code}\left(A\right)-\eta \log L_{A}\left(D\right),\\ q_{\beta }\left(A|D\right) & \propto \exp[-\beta E_{A}\left(D\right)],\\ P_{\mathrm{SpaceMix}}\left(\cdot |D\right) & =\sum_{A\in A}q_{\beta }\left(A|D\right)P_{A}\left(\cdot |D\right). \end{array}$$

On an infinite countable class, β=0 is excluded from this Gibbs definition because its partition function diverges. This is structurally analogous to a Solomonoff mixture under two substitutions: computable semimeasures ν become mm-model predictors $P_{A}$, and K(ν) becomes the computable code length Code(A). At β=1, λ=ln2, and η=1, the code and evidence terms give $2^{-\mathrm{Code}(A)}L_{A}\left(D\right)$, with the additional fixed GW-distortion penalty. Increasing β sharpens the full regularized energy. This convention differs from the evidence mixture $P_{\beta }$ of Proposition 15, which tempers only the distortion; the two agree at the calibration βλ=ln2, βη=1.

The data-dependent selector of Section 7 is the model-selection analogue obtained by replacing the fixed population distortion with $d_{\mathrm{GW},p}^{p}\left({\hat{S}}_{n},M_{\theta }\right)$. The sequential dominance result below does not permit that replacement inside its prior weights; data-independence of $D_{A}$ is essential.

**Proposition 14** (Single-space selection is the zero-temperature limit)**.**  *Assume that A is finite or countable with the infimum of the energy attained and isolated, and that $\sum_{A}e^{-\beta E_{A}\left(D\right)}<\infty$ for large β. As β→∞, $q_{\beta }\left(\cdot |D\right)$ concentrates on the minimizers of*

$$D_{A}+\lambda \mathrm{Code}\left(A\right)-\eta \log L_{A}\left(D\right),$$

*recovering a single MAP predictor when the minimizer is unique; with ties, the limit is the uniform mixture on the finite minimizer set. Choosing one tied minimizer requires a separate tie-breaking rule.*

**Proof.**  Let $E_{*}$ be the minimum and choose $\beta_{0}$ with finite partition function. The minimizer set has finite cardinality *m*, since each minimizer contributes $e^{-\beta_{0}E_{*}}>0$. For $\beta \geq \beta_{0}$, each nonminimal weight $e^{-\beta (E_{A}-E_{*})}$ tends to zero and is dominated by the summable sequence $e^{-\beta_{0}\left(E_{A}-E_{*}\right)}$. Dominated convergence shows that their total tends to zero. Each minimum has unnormalized relative weight one, so its limiting probability is 1/m.    □

### 12.2. Why the Mixture Is Closer: Dominance and Regret

**Proposition 15** (Space-mixture dominance)**.**  *Fix β≥0. Assume all component laws are probability laws on the same sequential sample space (with termination included when necessary), and all conditionals below have positive denominators along the evaluated history. Assume: (i) the candidate class A is finite or countable with Kraft-summable codes, $\sum_{A\in A}2^{-\mathrm{Code}(A)}\leq 1$; (ii) each GW distortion $D_{A}\geq 0$ is a* data-independent *constant of the candidate (a property of the population source and the candidate, not of $x_{1:n}$); and (iii) the sequential predictor is the Bayes conditional of the space-weighted joint mixture evidence*

$$P_{\beta }\left(x_{1:n}\right)=\sum_{A\in A}2^{-\mathrm{Code}(A)}e^{-\beta D_{A}}P_{A}\left(x_{1:n}\right),\;P_{\mathrm{SpaceMix}}\left(x_{t}|x_{<t}\right):=\frac{P_{\beta }\left(x_{1:t}\right)}{P_{\beta }\left(x_{1:t-1}\right)}.$$

*Under the calibration βλ=ln2, βη=1, this conditional coincides with the posterior-weighted mixture of Definition 8; for other parameter choices, the frozen-weight mixture is a different predictor and the bound below does not apply. Then, for every candidate $A^{★}\in A$,*

$$P_{\beta }\left(x_{1:n}\right)\;\geq \;2^{-\mathrm{Code}(A^{★})}e^{-\beta D_{A^{★}}}P_{A^{★}}\left(x_{1:n}\right),$$

*and consequently,*

$$\sum_{t=1}^{n}\;\left[-\log P_{\mathrm{SpaceMix}}\left(x_{t}|x_{<t}\right)\right]\;-\;\sum_{t=1}^{n}\;\left[-\log P_{A^{★}}\left(x_{t}|x_{<t}\right)\right]\;\leq \;\mathrm{Code}\left(A^{★}\right)\ln2+\beta D_{A^{★}}.$$

**Proof.**  The displayed evidence inequality is one term of the sum. By (i), $P_{\beta }$ is a subprobability with initial mass

$$W=P_{\beta }\left(\epsilon \right)=\sum_{A}2^{-\mathrm{Code}(A)}e^{-\beta D_{A}}\leq 1.$$

Because the conditional in (iii) divides by this same mixture evidence, the exact telescoping identity is

$$\sum_{t\leq n}-\log P_{\mathrm{SpaceMix}}\left(x_{t}|x_{<t}\right)=-\log P_{\beta }\left(x_{1:n}\right)+\log W.$$

Taking −log of the evidence inequality and subtracting $-\log P_{A^{★}}\left(x_{1:n}\right)$ gives the sharper regret bound $\mathrm{Code}\left(A^{★}\right)\ln2+\beta D_{A^{★}}+\log W$. The displayed proposition follows because logW≤0. Data-independence (ii) is what allows the distortion tilt to be a fixed prior reweighting; if the distortion were computed against a data-dependent target such as ${\hat{S}}_{n}$, the weights would depend on $x_{1:n}$, meaning that this sequential telescoping argument would fail and that no such uniform bound would hold in general.    □

In information-theoretic terms, this is a *redundancy* bound: the excess cumulative code length of the mixture code over the best included candidate is at most the candidate’s description length plus its distortion tilt, which is the coding-redundancy form of Solomonoff’s bound with a structured-approximation price.

This is the mm-model analogue of Solomonoff’s redundancy bound with an additional GW-distortion price. A single MAP space has no such mixture guarantee. The result is only competitiveness within the enumerated coded family, not universal dominance. Furthermore, it is not machine-invariant or a computable approximation theorem for the universal semimeasure. The β>0 distortion tilt is not exercised in the numerical validation programme of Section 14.

**Proposition 16** (Finite truncation of SpaceMix)**.**  *Let $A_{N}\subset A$ be a finite coded subset and define the truncated joint mixture*

$$P_{\beta ,N}\left(x_{1:n}\right)=\sum_{A\in A_{N}}2^{-\mathrm{Code}(A)}e^{-\beta D_{A}}P_{A}\left(x_{1:n}\right).$$

*For every included candidate $A^{★}\in A_{N}$,*

$$-\log P_{\beta ,N}\left(x_{1:n}\right)+\log P_{A^{★}}\left(x_{1:n}\right)\leq \mathrm{Code}\left(A^{★}\right)\ln2+\beta D_{A^{★}}.$$

*If $P_{\beta ,A}$ denotes the full countable mixture and*

$$r_{N}\left(x_{1:n}\right)=1-\frac{P_{\beta ,N}\left(x_{1:n}\right)}{P_{\beta ,A}\left(x_{1:n}\right)}\in \left[0,1\right),$$

*then the additional unnormalized negative log-evidence paid by truncating to $A_{N}$ is exactly*

$$-\log P_{\beta ,N}\left(x_{1:n}\right)+\log P_{\beta ,A}\left(x_{1:n}\right)=\log\frac{1}{1-r_{N}\left(x_{1:n}\right)}.$$

*These identities assume positive full and truncated evidence. For normalized sequential predictors, let $W_{N}=P_{\beta ,N}\left(\epsilon \right)$ and $W=P_{\beta ,A}\left(\epsilon \right)$. The cumulative log-loss difference is instead*

$${\mathrm{Loss}}_{N}-{\mathrm{Loss}}_{\mathrm{full}}=\log\frac{1}{1-r_{N}}+\log\frac{W_{N}}{W}.$$

*If truncated evidence vanishes, its loss is infinite. Finite SpaceMix remains competitive with each included candidate by the first inequality (normalizing its subprobability prior can only reduce its negative log-evidence).*

**Proof.**  The first display is the same one-term lower bound as Proposition 15, applied to the finite sum. For the truncation identity, write $P_{\beta ,N}=\left(1-r_{N}\right)P_{\beta ,A}$ and take the negative logarithms.    □

**Remark 14** (Computable reading of SpaceMix)**.**  *Proposition 16 is the version that can actually be implemented. The mm-model list may come from a finite dictionary, a prefix-code enumeration cut off by code length, beam search, reversible-jump exploration, or a hand-built collection of metric, graph-induced, tree, and manifold mm-spaces. The theorem gives no protection against a good candidate that was never enumerated.*

**Remark 15** (Three levels and what each collapse discards)**.** 
$$\begin{array}{c} \underset{\mathrm{Parts}\;I–\mathrm{VI}}{\underset{︸}{\mathrm{single}\;\mathrm{kernel},\;\mathrm{fixed}\;\mathrm{RKHS}}}\\ \cap \\ \underset{\mathrm{Section}\;7}{\underset{︸}{\mathrm{one}\;\mathrm{GW}\text{-}\mathrm{selected}\;\mathrm{geometry}}}\\ \cap \\ \underset{\mathrm{this}\;\mathrm{section}}{\underset{︸}{\mathrm{universal}\text{-}\mathrm{form}\;\mathrm{mixture}\;\mathrm{over}\;\mathrm{spaces}}}\\ ↓\;\left(\;A\;\to \;\mathrm{all}\;\mathrm{lower}\text{-}\mathrm{semicomputable}\;\mathrm{semimeasures},\;\mathrm{Code}\;\to \;K\;\right)\\ M. \end{array}$$

*The GP step discards everything beyond second order when the chosen category is Gaussian, while the arg min step discards the mixture (MAP) in every category. This section undoes the second collapse by mixing over spaces. If the components are Gaussian, then $P_{\mathrm{SpaceMix}}$ is a mixture of Gaussians and can therefore represent non-Gaussian and multimodal distributions; if the components include trees, graphs, Banach models, topological predictors, and direct predictive laws, then the mixture becomes broader. Full fidelity would additionally let A range over all lower-semicomputable semimeasures with true Kolmogorov weights, which this construction does not claim.*

**Remark 16** (Limits and an information-geometric reading)**.**  *$P_{\mathrm{SpaceMix}}$ remains a computable surrogate: the sum runs over a coded candidate class, not all programs, and Code is a description-length proxy for K. It is closer to M than a single RKHS or a single selected space in two precise senses: first, it has the mixture form, and second, it inherits the code-plus-distortion regret bound of Proposition 15; however, it is not equal to M. For a finite candidate list on a common observation space, mixing probabilities is affine in the probability coordinates. No smooth-manifold or global information-geometric structure is assumed for the universal semimeasure class. The dominance bound gives the precise operational comparison used here.*

## 13. The Universal Predictor and the Metric–Measure Projection Tower

The mixture of Section 12 is a mixture over coded *spaces and their induced predictors*. Lifting it one step further, to a mixture over *programs* or all lower-semicomputable semimeasures, gives the most primitive object of the theory and reorganizes the whole construction as a tower of projections, with the RKHS route at the bottom. Two things are easy to get wrong here and we are explicit about both: first the form of the posterior, and second, which transformations lose information and which retain their input kernel.

### 13.1. Level 1: The Universal Algorithmic Experiment

Fix a universal prefix machine for description complexity and a universal monotone machine $U_{m}$ for output prediction. The monotone form is

$$M\left(x\right)=\sum_{p\in P_{x}}2^{-|p|},$$

where $P_{x}$ consists of minimal input prefixes that make $U_{m}$ output a string beginning with *x*; it is prefix-free. Consistency carries the likelihood in this presentation. A universal measure-mixture presentation uses an effective enumeration of lower-semicomputable semimeasures $\nu_{p}$, with prefix description weights:

$$\tilde{M}\left(x\right)=\sum_{p}2^{-|p|}\nu_{p}\left(x\right),\;\pi_{D}\left(p\right)=\frac{2^{-|p|}\nu_{p}\left(D\right)}{\tilde{M}\left(D\right)}.$$

Assume positive evidence. With suitable universal description weights, $\tilde{M}$ and *M* dominate each other up to constants; they need not coincide. The posterior mixture identity is exact for $\tilde{M}$ and its component conditionals. For probability-valued prediction, include termination as an outcome, including any initial mass deficit; this convention is also used in the Hellinger and KL comparisons below. Conditioning the monotone program experiment likewise gives a posterior on consistent inputs; one must not insert a second likelihood factor into its consistency sum.

### 13.2. Level 2: The Posterior Explanation Geometry

Use the metric quotient and completion specified in Equation (3), with pushed-forward posterior mass:

$$S_{D}=\left(Q,d_{\exp},\pi_{D}\right).$$

A predictive zero-distance quotient is canonical relative to the declared history law. A syntactic algorithmic construction instead requires a separately specified exact metric; raw conditional program complexities do not descend automatically to semantic classes. The behavioral transfer results compare a declared measurable behavior map with its image under their faithfulness assumptions. They do not assert that an arbitrary data corpus reconstructs the universal explanation space.

### 13.3. Level 3: The Covariance Projection

Project the posterior-weighted feature family onto its second moment:

$$\Pi_{\mathrm{cov}}:\;\left(P,\pi_{D},\phi_{p}\right)\;⟼\;k_{D}\left(x,y\right)=\sum_{p}\pi_{D}\left(p\right)\left(\phi_{p}\left(x\right)-m_{D}\left(x\right)\right)\left(\phi_{p}\left(y\right)-m_{D}\left(y\right)\right),$$

with $m_{D}=\sum_{p}\pi_{D}\left(p\right)\phi_{p}$, assuming finite pointwise second moments. This centered covariance equals the uncentered SFM second moment minus $m_{D}\left(x\right)m_{D}\left(y\right)$; the two agree only for zero mean. Level 4 is the existing RKHS/GP layer $H_{k_{D}}$, $f∼\mathrm{GP}(0,k_{D})$, $T_{k_{D}}$. For Gaussian regression at Level 4, Proposition 10 shows that GP conditioning and KRR give the same finite representer expansion. This is a computational realization of the Level-4 object, not another projection of the universal experiment.

### 13.4. The Tower and What Each Projection Discards


$$\begin{array}{ccccc} P & \overset{\;\mathrm{posterior}\;}{\to } & \left(Q,\;d_{\exp},\;\pi_{D}\right) & \overset{\;\mathrm{behavior}\;}{\to } & \left(X,\;d_{\mathrm{Sol}},\;\mu_{D}\right)\\ & & ↓\;\Pi_{\mathrm{cov}} & & ↓\;D2\mathrm{KE}\\ & & k_{D} & ⟶ & H_{k},\;\mathrm{GP}\left(0,k\right),\;T_{k} \end{array}$$

**Proposition 17** (Information lost by covariance and unmarked comparison)**.**  *The map from general posterior feature laws to their covariance is not injective. Consequently, a covariance kernel alone does not in general determine the posterior feature law or its program identities. Unmarked GW comparison also does not identify external task labels. In contrast, a PSD kernel and its RKHS as an evaluated function space determine one another, and a centered Gaussian process determines its covariance kernel.*

**Proof.**  The scalar law assigning probability 1/2 to each of −1,1, and the law assigning probabilities 1/4,1/2,1/4 to $-\sqrt{2},0,\sqrt{2}$, both have mean zero and variance one but are different. Applying these random scalars to a fixed deterministic feature gives identical covariance kernels and different feature laws. Relabeling marks leaves an unmarked mm-space unchanged. Finally, the reproducing identity recovers k(x,y) from its RKHS, while k(x,y)=E[f(x)f(y)] recovers covariance from the centered GP.    □

**Figure 4:**
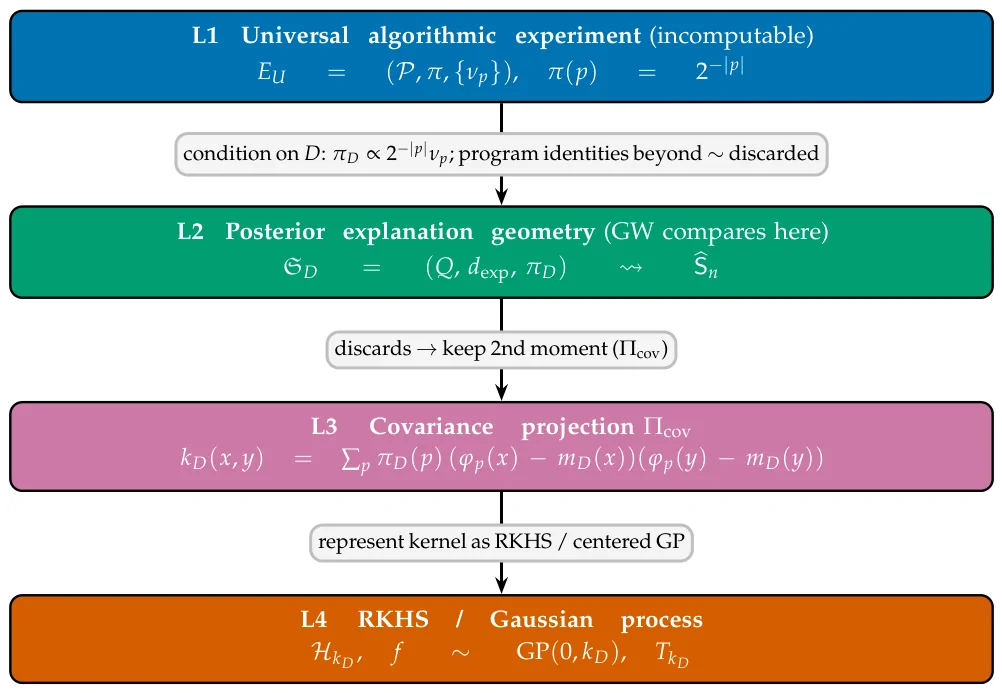
The projection tower of Proposition 17. The universal levels are generally incomputable. Covariance reduction can lose posterior information, whereas RKHS and centered-GP representation retain the specified kernel. Comparison lives at every floor: predictive deficiency Δ and regret $R_{T}$ at L1, Gromov–Wasserstein at L2, kernel norm/alignment/spectrum at L3–L4. GW is thus the metric–measure floor of a taller comparison ladder.

A covariance representation is generally insufficient to reconstruct the universal experiment, although subsequent RKHS and centered-GP representations retain the specified kernel. Mixing over models restores a mixture representation; it does not invert covariance reduction or recover omitted programs. Figure 4 distinguishes these operations.

### 13.5. The Comparison Principle at the Top

At Levels 3–4, one compares kernels (norms, alignment, spectra); at Level 2, geometries (GW); and at Level 1, one should compare *predictive experiments*. For a candidate predictor $P_{A}\left(\cdot |D\right)$, define the *Solomonoff deficiency*

$$\Delta \left(A;D\right)=\underset{L_{A}\left(D\right)}{\underset{︸}{-\log P_{A}\left(D\right)}}-\underset{L_{U}\left(D\right)}{\underset{︸}{\left(-\log M(D)\right)}}+K\left(A\right)\ln2$$

and sequentially define the cumulative predictive divergence

$$R_{T}\left(A\right)=\sum_{t=1}^{T}\mathrm{KL}\left(M\left(\cdot |x_{<t}\right)\;∥\;P_{A}\left(\cdot |x_{<t}\right)\right).$$

Small cumulative KL controls predictive discrepancy along the specified histories. A realized penalized log-evidence difference Δ alone does not guarantee future predictive closeness. These criteria compare probability predictions and require separate transfer assumptions to relate them to GW or kernel norms. The mm-model mixture $P_{\mathrm{SpaceMix}}$ of Section 12 controls a computable surrogate of this yardstick: Proposition 15 gives regret at most $\mathrm{Code}\left(A^{★}\right)\ln2+\beta D_{A^{★}}$ against any included coded candidate. This comparison is internal to the enumerated mm-model family; it is not a bound against the incomputable *M*.

### 13.6. Conceptual Order and Kernel Recoveries

The conceptual order is then

$$\begin{array}{c} \;\begin{array}{c} \mathrm{universal}\;\mathrm{posterior}\;\mathrm{over}\;\mathrm{programs}\\ ↓\\ \mathrm{posterior}\;\mathrm{algorithmic}\;\mathrm{geometry}\\ ↓\\ \mathrm{kernel}/\mathrm{RKHS}/\mathrm{GP}\;\mathrm{projection}, \end{array}\; \end{array}$$

Existing constructions occupy distinct levels: the KC/D2KE kernel applies bounded distance features to a declared algorithmic dissimilarity or metric surrogate; the uncentered SFM kernel weights those features by description length; its centered posterior variant is the covariance reduction above. RKHS and GP representations then retain the resulting specified kernel. The Schoenberg construction instead starts from a basepointed Hilbertian metric and returns its centered kernel. These identifications require the feature, normalization, and reference-measure conventions already stated.

**Remark 17** (Status of the tower)**.**  *Levels 1–2 are incomputable; the implementable content is the empirical ${\hat{S}}_{n}$ (compressor surrogate, Level 2), the mixture over candidate geometries (Section 12, a computable stand-in for the Level-1 posterior), and the Level-3–4 kernels. The tower distinguishes Solomonoff induction from its GP covariance projection: the GP represents covariance structure, while predictive fidelity is assessed through* Δ *and $R_{T}$.*

## 14. Results: Numerical Validation

The numerical summaries below are finite experimental observations. Their interpretation is limited to the stated corpora, splits, candidate families, and numerical solver settings; solver agreement at displayed precision is not an exact optimum certificate. Compression-based experiments use bz2 (with a three-compressor ensemble where stated); Gromov–Wasserstein is computed with the publicly available POT library [34] (multi-start Frank–Wolfe, entropic where noted). These are controlled probes of the finite surrogate pipeline, not a benchmark study and not evidence about the incomputable Solomonoff object itself.

**Computational conventions.** Strings are encoded as UTF-8 bytes; joint compression uses an eight-byte big-endian length, the first string, an eight-byte length, and the second string. NCD averages the two ordering-specific values, clips to [0,1.1], and has zero diagonal. The default compressor is bz2 at level 9. The recorded reference environment is Python 3.12.13 on macOS arm64, NumPy 2.4.6, SciPy 1.17.1, scikit-learn 1.9.0, and POT 0.9.7.post1. Unless overridden for a stated solver audit, the squared-GW routine uses six initializations with seed zero (product coupling, the identity when feasible, and random-cost optimal-transport couplings), POT’s square-loss solver with its version-specific default stopping settings, and the best feasible objective.

The 15-seed prediction protocol generates eight cellular-automaton strings per rule 30,90,110,184, on a periodic ring of width 24 over 24 recorded states, including the initial state. For replicate s=0,…,14, the initial-state seed is 1000rule+j+s, j=0,…,7. A permutation with seed 1000+s allocates 19 strings to training and 13 to testing. The periodic/noise comparison uses 20 strings per class, length 256, and period 31: each periodic motif is a random permutation of sixteen zeros and fifteen ones; noise bits are independent fair draws. Thus matching character frequencies is approximate, not exact. Training uses 60% of that corpus. Both tasks normalize NCD by the maximum over the full corpus and use all input observations as D2KE landmarks with γ=3; labels are held out, but this is a transductive protocol. Candidate selection minimizes training GW plus $2\times 10^{-4}C\left(k\right)$ over k=1,2,3,4. KRR adds $10^{-2}I$ to the training Gram matrix, uses one-hot targets and argmax for four classes, and a 0.5 threshold for binary targets. The 20-split comparison instead uses stratified splits, training-only scaling and landmarks, and a genuine one-neighbour baseline; its results are reported separately.

**Solver resolution.** All GW values are multi-start Frank–Wolfe *upper bounds* on a non-convex objective. On 30 random small instances, the certified bracket between the distance-law lower bound and the best feasible upper bound has mean width 0.0097, and Frank–Wolfe matches the exhaustive best-permutation upper bound on only 47% of instances; on the recovery corpus itself, the bracket is wider still (0.0068 lower vs. 0.0609 upper). Several selection decisions below ride on GW differences of order $10^{-3}$–$10^{-4}$, i.e., *below* certified resolution. Therefore, every selection claim in this section is heuristic-conditional: it is a statement about the multi-start Frank–Wolfe values made reproducible by fixed seeds, not a certified statement about exact GW. As a direct rank-stability audit, we repeated each candidate comparison over 30 independent solver-seed batches with eight starts per candidate: the three-family corpus selected k=3 in 30/30 batches (minimum winning score margin $3.72\times 10^{-4}$), while the ECA corpus selected the one-block candidate in 30/30 batches (minimum margin $6.65\times 10^{-4}$); the candidate GW values were identical to the reported precision on this platform. This supports numerical rank stability for these two finite comparisons, but does not close the exact-GW certification gap.

**Latent-structure recovery (E1)—calibrate-then-confirm, plus five-seed diagnostics.** Strings are generated from K=3 low-complexity cores (n=24 per corpus, five independent corpora). Four findings follow. First, the relational term alone does *not* identify the truth: over block candidates k∈{1,…,5} the fitted GW sequence is monotone decreasing (seed 1: 0.007,0.004,0.002,0.002,0.002; argmin k=5 on all five seeds)—the fitted values decrease in these runs. Monotonicity of exact minima is guaranteed only for nested admissible families; it is not a general property of arbitrary fitted partitions or local solver outputs. Second, identification in this experiment is carried by the BIC/MDL complexity score: adding $\frac{1}{2}\log_{2}n$ bits per fitted block parameter selects $k^{★}=3$ on a λ-window for four of the five seeds (e.g., seed 1: $\lambda \in [1.4\times 10^{-5},2.8\times 10^{-4}]$); for seed 2 *no* λ recovers k=3. This score penalizes fitted dimension but, because the real-valued block entries are not quantized and serialized, is not presented as a literal parameter prefix code. Third, the five-seed exploratory rule chooses λ by cross-validated relational risk without using ground truth: select $k^{★}\left(\lambda \right)$ on sub-train corpora and score the selected train-fitted block model on held-out relations. On the four recovering seeds it picks λ≈2–$3\times 10^{-4}$ and recovers K=3; on the failing seed it picks $1.26\times 10^{-4}$ and selects k=4. Fourth, a distinct calibrate-then-confirm protocol quantifies transfer under a matched synthetic generator: on 20 calibration corpora (60 strings each, three-way fit/validation/test splits), λ is fixed by a maximin rule jointly maximizing known structured k=3 recovery and known noise k=1 rejection. Thus ground truth is used at calibration, but the 40 confirmation corpora are fresh and never used for tuning. At the frozen $\lambda =3.2\times 10^{-5}$, the selector recovers k=3 in 34/40 runs (Wilson 95% interval [0.71,0.93]) and selects k=1 on 39/40 fresh noise corpora ([0.87,1.00]). These frequencies estimate transfer for this declared generator and candidate family; they are not an unsupervised real-data calibration guarantee.

**Memorizer control (why fitted dimension must be penalized).** With only the Kraft-valid model-index code (a few bits for *k*), extending the family to k=n produces a memorizing candidate with GW =0 at 10.9 bits, and it *wins* at the small-λ end. Adding the declared BIC/MDL fitted-dimension penalty raises the memorizer’s complexity score to 699 bits, and it loses at every λ on the grid. This control is informative over the operative λ window, which contains the frozen $3.2\times 10^{-5}$ and cross-validated $\approx 2\times 10^{-4}$ values, not in the unpenalized λ→0 limit, where any fixed additive penalty is eventually dominated by an exact-fit memorizer. This is evidence that fitted flexibility must be penalized; it is not a Kraft test over all real-valued block matrices.

**Structureless-data negative control (E2).** On i.i.d. uniform random strings (no algorithmic structure), the same pipeline with the same data-driven λ rule selects the trivial geometry k=1 (and k=1 wins for every $\lambda \geq 10^{-5}$ on the grid); at confirmation scale the latent-structure protocol (E1) selects k=1 on 39/40 fresh noise corpora. No spurious preference for structured geometries.

**Inductive block recovery (E3)—train-fitted and held-out-scored.** Fitting *k* blocks on a training half and scoring the *train-fitted* block distances against held-out relations (each held-out point assigned to its nearest training medoid; the held-out set never fits its own model): training GW is monotone in *k* (0.0068→0.0006), while the inductive held-out GW diagnostic is minimized at k=2—*not* the true K=3. The reported selection uses the *training* score $d_{\mathrm{GW},2}^{2}\left({\hat{S}}_{\mathrm{tr}},M_{k}\right)+\lambda C\left(k\right)$, not held-out GW plus the penalty; that training fit–complexity tradeoff selects $k^{★}=3$ on the window $\lambda \in [6.3\times 10^{-5},4.0\times 10^{-4}]$, containing the independently cross-validated $\lambda \approx 2\times 10^{-4}$. Held-out relational fit is a separate generalization diagnostic and under-segments in this run.

**Synthetic spectral calibration (E4).** With orthonormal features and Solomonoff weights $2^{-\ell_{i}}$, the spectrum of the generalized synthetic feature mixture tracks the assigned masses: the ratio $\lambda_{i}/2^{-\ell_{i}}$ lies in [1.00,1.00] at exact orthogonality and degrades gracefully to [0.75,1.02] at feature coherence 0.1. This is a controlled identity in the sense of Theorem 2—the weights are inserted by hand on synthetic features; it is not a recovery from compression data—and the conservative lower band can be vacuous when δκ≥1; the relative row-condition bound remains informative if δ<1.

**GW-to-kernel stability (E5).** Over 12 random finite mm-space pairs at γ=1, the transported-kernel ratio $\Delta_{\pi^{★}}/d_{\mathrm{GW},2}$ has mean 0.437 and maximum 0.687, below the constant $2\sqrt{2}\;\gamma =2.83$ of Proposition 13. A sanity check of the mechanism, not an estimate of the worst case.

**Pairing-conditional metric repair (E6).** On the recovery corpus, the self-delimiting NCD pairing is metric (0 triangle violations at tolerance $10^{-10}$), while naive concatenation produces 16 violations (worst excess 0.019); the subdominant metric closure removes all of them. The *powered* block-GW objective moves by $1.12\times 10^{-4}$ (from 0.00692 to 0.00681). Equivalently, the unpowered distance moves from 0.083196 to 0.082522, a change of $6.75\times 10^{-4}$, inside the unpowered bound $2^{-1/2}\delta /\mathrm{diam}=3.41\times 10^{-2}$. Applying the local power-function inequality gives the corresponding powered-objective bound $5.68\times 10^{-3}$. Repair is a guarded no-op under the default pairing and a genuine controlled correction under the naive one.

**Cross-compressor ensemble (E7).** Across zlib, bz2, and lzma on a controlled 14-string corpus, the top candidate (ultrametric) is stable for all three compressors, but the distance-level ensemble instability is large (0.28 on distances ≤1.1), and the ensemble’s planted-ambiguity detector caught 0 of 2 planted items—a negative finding reported as such. Because all three compressors are finite-window statistical (LZ/BWT-family) coders, agreement across them certifies *family-relative* robustness only, not compressor-independent algorithmic structure: data whose structure is algorithmic but not statistical (pseudorandom generator output, digits of π) would be misplaced identically by every member of the ensemble.

**Occam mass and its collapse diagnostic (E8).** Re-running recovery with the Occam-reweighted mass $\hat{\mu }\propto 2^{-\hat{K}}$ on compressed lengths collapses the effective sample size from 24 to 2.0 and destroys the recovery window entirely—precisely the collapse mode that the ESS diagnostic of Section 5 is designed to flag. On corpora of near-equal compressed lengths, the Occam and uniform weightings agree; on this corpus, the experiments support the uniform-mass (KC) branch of Theorem 1, and the Solomonoff-weighted branch is exercised only in the synthetic spectral-calibration study (E4). We report ESS alongside every Occam-weighted run.

**Marked-coupling mechanism check (E9).** The fused (marked) objective of Section 7 is run once on the labeled recovery corpus with η=1 (POT’s fused-GW solver at α=1/(1+2η), the mapping matching our $\frac{1}{2}$-relational convention): total objective 0.0021 with mark-loss term 0.0000 at four decimal places; the computed coupling has negligible cross-label mark loss at the reported precision. This rounded value does not certify exact zero loss or global optimality. This first marked experiment isolates the coupling mechanism; it is not by itself an evaluation of marked candidate selection or out-of-sample prediction. The real-data marked-selection study, introduced below, supplies that second test.

**Held-out prediction through selected geometry (E10)—baselines and intervals.** The results are summarized in Table 8. On periodic-versus-noise, prediction through the unmarked selected geometry reaches raw accuracy 0.992±0.011 and balanced accuracy 0.991±0.012, against 1.000±0.000 for the raw NCD kernel and NCD-3NN. On the four-class cellular-automaton task the selector collapses to k=1 and obtains raw accuracy 0.154±0.033. That raw number should not be interpreted as evidence of worse-than-chance signal: the globally balanced corpus is split without stratification, so the training-majority class is systematically depleted in the test set. The split-aware constant-training-majority null is 0.144±0.025. When every class occurs in the test set, a constant predictor has balanced accuracy exactly 1/4. The implementation averages recall over classes present in the test set. At seed 13 (zero-based), the test class counts are (6,0,3,4) and the training-majority class is the absent class 1, giving balanced accuracy zero. The other fourteen splits give 1/4, hence the reported null 0.233±0.033. In the reported scoring, the selected geometry obtains 0.247±0.039, effectively the four-class chance value 0.25, while raw NCD and NCD-3NN obtain 0.652±0.075 and 0.810±0.069.

The collapse has a direct structural explanation. With η=0, the objective in (5) sees only pairwise compression geometry and code length. It is unchanged by any permutation of the class labels, so it cannot distinguish two tasks with identical inputs and contradictory labelings. On the cellular-automaton data, training GW plus the MDL penalty favors the one-block candidate. The implemented extension assigns observations to their nearest training medoid, uses training-fitted block means for off-diagonal distances, and sets the diagonal to zero. Its D2KE kernel uses all observations as uniformly weighted landmarks. At k=1, the resulting *N*-point distance matrix has a single off-diagonal value *c*. Writing $q=e^{-3c}$, its kernel is

$$K=aI+b11^{⊤},\;a={\left(1-q\right)}^{2}/N,\;b=q^{2}+2q\left(1-q\right)/N.$$

For *m* training points, one-hot class targets, and ridge $r=10^{-2}$, every test point has class-*j* score $bn_{j}/\left(a+r+bm\right)$, where $n_{j}$ is the training class count. Thus exact arithmetic with a fixed tie rule gives the constant training-majority classifier. The recorded 0.154±0.033 and 0.247±0.039 are floating-point implementation outputs: tied training counts allow round-off to change the argmax across test points. They do not establish a gain over the exact one-block null. The certificate in Lemma 2 concerns the equilateral blow-up; the constant-prediction conclusion additionally uses this specific extension, kernel, and learner. The failure is not merely an unlucky optimizer: it is an instance of the label-blindness built into unmarked GW.

We also exercise the sufficient certificate of Proposition 2 on the same declared k∈{1,2,3,4} menu. Table 9 reports $\lambda_{★}=B\left(D\right)/\left(2+\log_{2}n\right)$ over 15 seeded training splits at the held-out-prediction setting (E10), $\lambda =2\times 10^{-4}$. The implication is sound in all 60 runs: whenever $\lambda >\lambda_{★}$, the selected model has k=1. It is deliberately not a characterization. On the ordinary ECA corpus it fires in only 4/15 runs although collapse occurs in 15/15. In a 61-point λ sweep on three ECA and three periodic-versus-noise seeds, the largest tested value still selecting k>1 lies below $\lambda_{★}$ by factors 1.22–3.14; no implication is violated. The exact identity in Lemma 2 is also checked numerically: the solver-to-B(D) ratio is one to numerical precision on every split.

The supplementary task-aware experiment uses a nested stratified validation split to rank already constructed block kernels and the raw geometry by predictive loss plus code length. It reaches 0.785±0.139 and usually retains the raw geometry, while raw D2KE–NCD reaches 0.838±0.067 and NCD-1NN reaches 0.755±0.043 on the *same* 20 splits. Every raw and block geometry considered by the nested selector is evaluated with the same KRR learner; the NCD-1NN row is a separate baseline, not the predictor used for the raw candidate. These results show that predictive alignment can prevent unmarked collapse, but the geometry-selection layer has not improved on the raw D2KE compression baseline. This selector is label-aware at the model-selection level; unlike the real-data marked-selection study below, it does not optimize the fused marked-GW coupling.

Accordingly, “geometric priority” is not a valid unconditional proposition. The defensible statement is that geometry supplies a structural prior, while supervised model selection requires a marked or held-out predictive term. The real-data marked-selection study below tests this requirement; no real-data superiority claim is made.

**Real-data marked selection on SMS spam (E11).** To evaluate label-aware geometric selection on real data, we conduct a fused/marked-GW experiment on a deterministic balanced subset of the public UCI SMS Spam Collection [77]: 100 ham and 100 spam messages. Ten repeated outer splits are stratified 70/30. Within each outer training set, a second stratified split chooses η∈{0,0.25,1,4}; the candidate menu is k∈{1,2,3,4}, $\lambda =2\times 10^{-4}$, the compressor is bz2, and only training labels and training landmarks form the block marks and D2KE predictor. The outer test labels are excluded from fitting and practical model selection; they are used for final scoring and the explicitly declared post-hoc oracle diagnostic. Balanced accuracy is primary. Because the ten splits reuse one fixed corpus, the displayed standard deviations are descriptive rather than independent-sample confidence intervals. Table 10 reports the results.

Adding marks to the coupling is *insufficient for the tested block family*: the unsupervised average-linkage block partitions remain label-mixed (the best training adjusted Rand index over *k* is 0.00045±0.00047), so their learned marks differ too little to overcome the distortion and complexity preference for the one-block model. The post-hoc test-label oracle confirms that changing only the rule that selects among the four implemented block kernels cannot repair the pipeline: every *k* obtains 0.500±0.000, against 0.910±0.048 for raw D2KE–NCD, and raw geometry is strictly better on all ten outer splits. This is evidence of candidate-family or *quotient inadequacy at the tested sample size*, not absence of predictive information in the raw NCD matrix. Its scope is the fixed 200-message subset, the 140 outer-training observations per split, average-linkage partitions, D2KE construction, and KRR learner; it does not distinguish inherent family inadequacy from sample starvation and does not rule out a larger sample, different partition generator, supervised quotient family, kernel, or learner. A sample-size curve is required before making a population-level inadequacy claim. The raw-geometry safeguard prevents collapse but exactly matches rather than improves upon raw D2KE–NCD; the character baseline remains better. The supported finite-experiment design rule is conjunctive:

$$\begin{array}{c} \begin{array}{c} \mathrm{task}\text{-}\mathrm{aligned}\;\mathrm{comparison}\;+\;\mathrm{adequate}\;\mathrm{candidate}\;\mathrm{class},\\ \mathrm{including}\;a\;\mathrm{safe}\;\mathrm{source}\;\mathrm{representation}\;\mathrm{when}\;\mathrm{adequacy}\;\mathrm{is}\;\mathrm{unproved} \end{array} \end{array}$$

Neither unmarked geometry nor marking an impoverished quotient family is sufficient.

### Mechanism Checks for Parts III–VI

The second suite asks whether the numerical pipeline reproduces the main *mechanisms* of Parts III–VI where the ground truth is known. Table 11 reports the corresponding checks. They are deliberately synthetic falsifiable regression tests of mechanisms, not benchmarks.

**What these experiments do and do not establish.** They establish, at toy scale and under fixed seeds, that BIC/MDL-penalized selection recovers planted structure on most but not all corpora; that the relational term alone never does; that the memorizer control fails without a fitted-dimension penalty; that PSD repair, spectral calibration, effective-dimension ordering, and pullback preservation behave as the theory predicts; and that the marked mechanism runs. They also establish two negative results: unmarked selection can be far worse than the raw compression kernel for prediction, and conventional character features beat all compression methods on the one natural-data task. They do not estimate distance to the true Solomonoff semimeasure, establish real-data superiority, or provide an unsupervised real-data rule for calibrating the latent-structure penalty, and are not certified at the exact GW solver level.

## 15. Error Decomposition

The ideal object is an incomputable Solomonoff mm-space S. The learned object is a computable finite geometry ${\hat{M}}_{\hat{\theta }}$. The total error can be decomposed into conceptually distinct terms.

Let $S^{\left(L\right)}$ be a depth-*L* truncation of the ideal Solomonoff space, ${\hat{S}}_{n,C}$ the empirical space built from compressor *C*, $M_{\theta^{★}}$ the best candidate in the chosen model class, and $\hat{\theta }$ the output of a numerical algorithm.

**Theorem 3** (Solomonoff–Gromov error decomposition)**.**  *For any p≥1,*

(15) $$\begin{array}{cc} d_{\mathrm{GW},p}\left(S,M_{\hat{\theta }}\right)\leq & \underset{\mathrm{Solomonoff}\;\mathrm{truncation}}{\underset{︸}{d_{\mathrm{GW},p}\left(S,S^{\left(L\right)}\right)}}+\underset{\mathrm{compressor}\;+\;\mathrm{sampling}}{\underset{︸}{d_{\mathrm{GW},p}\left(S^{\left(L\right)},{\hat{S}}_{n,C}\right)}}\\ \; & +\underset{\mathrm{model}\text{-}\mathrm{class}\;\mathrm{approximation}}{\underset{︸}{d_{\mathrm{GW},p}\left({\hat{S}}_{n,C},M_{\theta^{★}}\right)}}+\underset{\mathrm{selection}\;\mathrm{displacement}}{\underset{︸}{d_{\mathrm{GW},p}\left(M_{\theta^{★}},M_{\hat{\theta }}\right)}}. \end{array}$$

**Proof.**  This is the triangle inequality for $d_{\mathrm{GW},p}$, applied through the chain

$$S\to S^{\left(L\right)}\to {\hat{S}}_{n,C}\to M_{\theta^{★}}\to M_{\hat{\theta }}.$$

   □

The last term is a distance between candidate geometries, not an objective optimization gap. Two exact minimizers can have positive mutual GW distance. For example, a uniform two-point source with distance 2 and equal-code candidates with distances 1 and 3 has two minimizers with squared GW distortion 1/4, while the candidates have mutual GW distance 1. Converting objective excess to selection displacement requires an additional identifiability or error-bound hypothesis.

Downstream errors must be compared on a common scale. If a kernelization satisfies $∥T_{M}^{\pi }-T_{N}∥\leq L_{\kappa }d_{\mathrm{GW},p}\left(M,N\right)$, apply $L_{\kappa }$ to the geometric triangle bound before adding an operator approximation error. Predictor risk then requires its own stability estimate. Entropic objective bias and solver objective gaps are recorded separately; they are not distances between selected geometries. Each term has a distinct interpretation.

**Truncation error.** This is the error from replacing the universal infinite program prior by descriptions of length at most *L*. When the prior is prefix-free, tail mass is controlled by Kraft-type bounds, although the exact rate depends on how the truncation is defined and on whether one truncates programs, outputs, or equivalence classes.

**Compressor and sampling error.** This term combines finite sample error with the discrepancy between *K* and the computable compressor *C*. Comparing compressors measures robustness within that family; it does not measure discrepancy from incomputable *K*. Sampling error can be controlled separately when a population surrogate and sampling law are specified.

**Approximation error.** This is the price of the chosen geometry class. A tree class may approximate prefix structure well but continuous manifolds poorly. A manifold class may approximate smooth physical data well but fail on hierarchical program-like data. The decomposition is a geometric decomposition before kernelization. If the selected geometry is subsequently passed through D2KE, a heat kernel, a negative-type repair, or an SFM construction, there is an additional kernelization defect measured at the kernel/operator level, as in Proposition 11. This structural loss is not part of the GW triangle inequality itself.

**Optimization error.** Exact GW optimization is generally difficult. Entropic GW, low-rank couplings, sliced variants, and landmark approximations introduce computational error. This term records that cost explicitly rather than hiding it inside the definition of the model. In computations it should be split, when possible, into an optimization gap and a regularization/discretization bias, for example the difference between the entropically regularized GW value and the unregularized GW value. The finite centered entropic bias is bounded in Proposition 19; geometry-dependent refinements and certification of a particular numerical run remain separate questions.

### Consistency of Empirical GW

**Theorem 4** (Consistency of empirical GW, with rate)**.**  *Let S=(X,d,μ) be a compact metric–measure space with diam(X)≤D<∞. Let $X_{1},\ldots ,X_{n}∼\mu$ be i.i.d. and $S_{n}=\left({\left\{X_{i}\right\}}_{i=1}^{n},\;d{|}_{X_{n}},\;n^{-1}\sum_{i}\delta_{X_{i}}\right)$. Then,*

$$d_{\mathrm{GW},p}\left(S_{n},S\right)\underset{n\to \infty }{\to }0\;\mathrm{almost}\;\mathrm{surely}.$$

*Moreover, if $r_{n}$ is any rate satisfying*

$$E\;W_{1}\left({\hat{\mu }}_{n},\mu \right)\leq r_{n},$$

*then*

$$E\;d_{\mathrm{GW},p}\left(S_{n},S\right)\leq C\left(D,p\right)\;r_{n}^{1/p}.$$

*In particular, if (X,d) has upper box-counting (Minkowski) dimension $d_{\mathrm{box}}$, then for p=2 and every $s^{\prime }>\max\left(d_{\mathrm{box}},2\right)$ there is a space-dependent constant $C_{s^{\prime }}\left(X,d,\mu \right)<\infty$ with*

$$E\;d_{\mathrm{GW},2}\left(S_{n},S\right)\;\leq \;C_{s^{\prime }}\left(X,d,\mu \right)\;n^{-1/(2s^{\prime })}.$$

*Here, the constant necessarily depends on the space’s covering profile, not only on its diameter and dimension exponent. More explicitly, under a covering bound $N\left(X,\varepsilon \right)\leq A\varepsilon^{-d_{\mathrm{box}}}$, it may be taken to depend on $A,D,s^{\prime }$. The theorem asserts the strict-exponent bound only. Critical exponents require a separate entropy or matching analysis; Euclidean matching results such as [78] do not by themselves establish a critical logarithmic rate on every compact metric space with the same box dimension.*

**Proof.**  *Almost-sure convergence.* The identity map embeds $S_{n}$ and S into the common space (X,d); using the (suboptimal) coupling induced by the empirical-to-population optimal transport plan $\pi_{n}\in \Pi \left({\hat{\mu }}_{n},\mu \right)$,

$$\begin{array}{cc} d_{\mathrm{GW},p}^{p}\left(S_{n},S\right) & \leq \frac{1}{2}\;\int \int \;{\left|d\left(x,x^{\prime }\right)-d\left(y,y^{\prime }\right)\right|}^{p}\;d\pi_{n}\left(x,y\right)\;d\pi_{n}\left(x^{\prime },y^{\prime }\right)\\ & \leq {\left(2D\right)}^{p-1}W_{1}\left({\hat{\mu }}_{n},\mu \right). \end{array}$$

The last inequality uses

$$|d\left(x,x^{\prime }\right)-d\left(y,y^{\prime }\right)|\leq d\left(x,y\right)+d\left(x^{\prime },y^{\prime }\right),\;\left|d\left(x,x^{\prime }\right)-d\left(y,y^{\prime }\right)\right|\leq 2D.$$

Under $\pi_{n}$, each of the two triangle terms integrates to $W_{1}\left({\hat{\mu }}_{n},\mu \right)$; the resulting factor 2 cancels the $\frac{1}{2}$ of Definition 2, so the constant is ${\left(2D\right)}^{p-1}$ with no residual $\frac{1}{2}$. By the Varadarajan/Glivenko–Cantelli theorem, ${\hat{\mu }}_{n}\to \mu$ weakly a.s.; on a bounded metric space, this implies $W_{1}\left({\hat{\mu }}_{n},\mu \right)\to 0$ a.s., giving $d_{\mathrm{GW},p}\left(S_{n},S\right)\to 0$ a.s. Taking expectations in the display gives $E\;d_{\mathrm{GW},p}^{p}\left(S_{n},S\right)\leq {\left(2D\right)}^{p-1}r_{n}$, and Jensen’s inequality (concavity of $t\mapsto t^{1/p}$) yields $E\;d_{\mathrm{GW},p}\left(S_{n},S\right)\leq {\left({\left(2D\right)}^{p-1}r_{n}\right)}^{1/p}=C\left(D,p\right)\;r_{n}^{1/p}$. The stated p=2 rate follows from the empirical $W_{1}$ rates of [79,80]: for every $s^{\prime }>\max\left(d_{\mathrm{box}},2\right)$, one has $E\;W_{1}\left({\hat{\mu }}_{n},\mu \right)\leq C_{s^{\prime }}^{\prime }n^{-1/s^{\prime }}$ (with a space-dependent constant). Sharper and entropic-GW-specific sample-complexity results are available [81,82]. A scope caveat for dynamics is that the theorem’s sampling model is i.i.d. draws from a fixed population mm-space; data generated by a dynamical system—a single trajectory with dependent samples—are *not* covered as stated, and an ergodic-sampling variant (Birkhoff-type, with mixing controlling the empirical-measure rate) is an open problem recorded in Section 23.    □

**Remark 18.**
  *This isolates the* sampling *term of the error decomposition; the compressor term (${\hat{d}}_{\mathrm{Sol}}$ vs. the ideal algorithmic distance) and the truncation term are separate and are not removed by sampling alone (Section 23). The square root in this p=2 upper bound comes from the displayed $W_{1}$ comparison and Jensen inequality. It is not asserted to be an intrinsic lower-bound obstruction for GW. The diameter dependence in ${\left(2D\right)}^{p-1}$ is explicit: it is order one for normalized compression dissimilarities, which are designed to lie near [0,1] (the E7 corpus reaches only 1.1), whereas an unnormalized cost must be rescaled or retain its actual diameter in the bound.*

**Remark 19.**
  *The theorem does not solve the incomputability of the true Solomonoff space. It says that if a population algorithmic mm-space is fixed and sampleable, then its empirical relational geometry is statistically consistent. The additional compressor approximation error must be handled separately.*

## 16. Spectral Solomonoff Approximation

### 16.1. The Ideal Spectrum

Earlier parts of the BAITML programme emphasize the complexity–spectrum correspondence [3,4,5]:

$$\lambda_{i}≍2^{-\ell_{i}},$$

where $\ell_{i}$ is a description length associated with the *i*-th feature, program, or canonical equivalence class. In the simplest ideal ordering $\ell_{i}=i$, this becomes

$$\lambda_{i}≍2^{-i}.$$

This is the clean toy case: every additional bit halves the spectral mass. It serves as the ideal calibration because it expresses the exact Occam/Solomonoff intuition in spectral form.

**Remark 20** (The sorted envelope depends on the program-counting function)**.**  *The ordering $\ell_{i}=i$ assumes* one *description per length. With realistic multiplicity—a program-counting function $N\left(L\right)=\#\left\{\omega :\ell \left(\omega \right)\leq L\right\}≍2^{\alpha L}$ for some α∈(0,1) (an additional counting hypothesis, not a consequence of Kraft)—the i-th sorted weight sits at length $\ell_{i}\approx \alpha^{-1}\log_{2}i$, so*

$$\lambda_{i}≍2^{-\ell_{i}}≍i^{-1/\alpha },$$

*a* polynomial *envelope (exponent 1/α>1), not exponential-in-i. Kraft also permits counting exponent one: for sufficiently small c>0, the counts $a_{L}=\left\lfloor c2^{L}/L^{2}\right\rfloor$ satisfy $\sum_{L}a_{L}2^{-L}<1$ but $N\left(L\right)≍2^{L}/L^{2}$. Hence strict inequality α<1 is not forced by code summability. Under the stated counting ansatz, the exponent α is an entropy rate: $\alpha =\lim_{L}L^{-1}\log_{2}N\left(L\right)$ is the exponential growth rate of the description ensemble (the topological entropy of the program tree), so the sorted Solomonoff spectrum is governed by an entropy. The exponential law $\lambda_{i}≍2^{-i}$ is the zero-entropy case with one effective description per length, more precisely, $\ell_{i}=i+O\left(1\right)$ gives this exact comparability, whereas N(L)=Θ(L) gives only $2^{-\Theta (i)}$; N(L)=O(1) would instead allow only finitely many descriptions. This matters for the trichotomy below: whether the globally sorted Solomonoff spectrum looks exponential, polynomial, or plateau is a property of N(L), i.e., of the domain’s algorithmic content, not a universal constant (on exponent conventions across the series: Part III writes the polynomial regime of a* fixed *kernel operator as $\lambda_{i}≍i^{-2s/d}$ (Sobolev smoothness s, input dimension d), which is the generic operator exponent a=2s/d of Section 17. The program-counting exponent α here is a different object: 1/α governs the* sorted Solomonoff envelope *across length classes, and is unrelated to the decay exponent a of any single kernelization).*

**Remark 21** (Machine invariance is regime-level)**.**  *Changing the reference universal machine shifts description lengths by additive constants on stable code families, and changing compressors perturbs finite distance and spectrum estimates. Raw eigenvalues and individual mode identities are therefore not invariant claims. The robust statements are regime-level: exponential toy decay when there is one effective description per length, polynomial sorted envelopes when program-counting multiplicity grows, finite-shell plateaux under truncation, and stability of these conclusions under reasonable compressor swaps.*

### 16.2. Spectral mm-Spaces

A compact positive trace-class covariance operator *T* with eigenvalues ${\left(\lambda_{i}\right)}_{i\geq 1}$ defines a Gaussian measure on a Hilbert space and hence a probabilistic metric object. One may compare two such objects directly through their covariance spectra. For the purposes of this paper, define the spectral discrepancy

$$d_{\mathrm{spec}}^{2}\left(T,T_{\mathrm{Sol}}\right)=\sum_{i\geq 1}{\left(\lambda_{i}-2^{-\ell_{i}}\right)}^{2}$$

whenever the sum is finite after a common ordering or matching.

This quantity is not a replacement for GW on data spaces; it is an operator-level proxy. It measures whether the learned geometry, after kernelization, produces the correct Solomonoff-style spectral mass distribution.

### 16.3. GW Interpretation of the Spectral Trichotomy

The three classical regimes become three different kinds of GW/spectral mismatch.

**Polynomial spectra.** If $\lambda_{i}∼i^{-a}$, then high-index modes receive much more mass than an exponential Solomonoff spectrum. Such kernels tend to over-weight high-complexity hypotheses relative to Solomonoff calibration. Here, *a* is the decay exponent of a fixed kernel operator, not the program-counting exponent α above.

**Plateau spectra.** A finite-depth Solomonoff truncation has length classes. Within a fixed length class, many programs have comparable weight. Thus, a plateau followed by a cutoff is a natural finite-depth approximation *within a single length class*. This does not weaken the plateau results of Parts III and V: those results are *capacity and finite-resolution learning statements*, where the learner only sees a finite shell, truncation, or effective rank. The globally sorted Solomonoff envelope answers a different question, namely, how mass is distributed *across* all length classes, and by the remark above, may be polynomial with exponent 1/α. Hence, the right reading is additive: plateaux are the correct local/finite-capacity regime, while the global envelope records the growth of the program-counting function.

**Super-smooth spectra.** Gaussian/RBF-type spectra may decay too fast, collapsing mass onto very low-frequency modes and under-weighting moderately complex but short-program structure. Such kernels look Occam-like but are not necessarily Solomonoff-calibrated.

**Definition 9** (Spectral Solomonoff calibration)**.**  *A kernel k with covariance eigenvalues $\lambda_{i}\left(k\right)$ is spectrally Solomonoff-calibrated to depth L if there is a matching of its first $N_{L}$ modes to description classes of length at most L such that*

$$\sum_{i=1}^{N_{L}}{\left|\lambda_{i}\left(k\right)-c_{L}2^{-\ell_{i}}\right|}^{2}\leq \varepsilon_{L},$$

*where $c_{L}$ is the normalizing constant for the truncated description prior.*

### 16.4. Relation to Solomonoff–Gromov Selection

The GW objective selects a geometry by matching pairwise distances. The spectral calibration objective tests the kernel/operator induced by that geometry. A convincing Solomonoff approximation should pass both tests:

$$\mathrm{relational}\;\mathrm{calibration}:\;d_{\mathrm{GW}}\left({\hat{S}}_{n},M_{\theta }\right)\;\mathrm{small},$$

and

$$\mathrm{spectral}\;\mathrm{calibration}:\;d_{\mathrm{spec}}\left(T_{k_{\theta }},T_{\mathrm{Sol}}^{\left(L\right)}\right)\;\mathrm{small}.$$

This two-level criterion separates generic simplicity priors from genuinely algorithmic geometry.

**Remark 22** (Why two levels are necessary, and a third for prediction)**.**  *The two levels address distinct but interacting factors of the master kernel (12), as identified in Remark 7: relational calibration tests the distance-defined feature dictionary together with the mass-weighted coupling, while spectral calibration tests the eigenvalue multiset of the resulting operator. Neither alone suffices. GW is invariant under measure-preserving isometry and does not identify an operator basis; the eigenvalue-only discrepancy $d_{\mathrm{spec}}$ is blind to eigenfunctions; and changing the reference measure can rotate eigenfunctions when the features are coherent. For supervised use, a third condition is needed that matches the marking/labeling, which is supplied by the marked objective (10). Relational-plus-spectral calibration constrains the prior geometry and spectrum, but not which response attaches to which input.*

## 17. The Metric–Measure Learning Core

We now spell out how the spectral regimes of Parts III–VI reappear from the metric–measure viewpoint. The key point is simple but important: an mm-space does not have a unique spectrum by itself; it has spectra *after choosing a canonical operator generated by the geometry*. This is not a defect, and is exactly the same choice already made in the earlier papers when passing from an algorithmic distance to D2KE, SFM, SKCO, an SGP covariance, or a deep pullback kernel.

### 17.1. Operator Functors from mm-Spaces

Let M=(Z,d,μ) be a bounded mm-space. A *kernelization functor* assigns to M a PSD kernel

$$\kappa_{M}\left(z,z^{\prime }\right)=\Psi \left(d,\mu \right)\left(z,z^{\prime }\right),$$

and hence a compact integral operator

$$T_{M}f\left(z\right)=\int_{Z}\kappa_{M}\left(z,z^{\prime }\right)f\left(z^{\prime }\right)\;d\mu \left(z^{\prime }\right).$$

Examples include the D2KE kernel (4), heat kernels, Schoenberg kernels, diffusion kernels on graphs, and the SFM/Occam kernel (12). The *mm-space spectrum* is the spectrum of $T_{M}$ for the chosen functor:

$$\lambda_{1}\left(M\right)\geq \lambda_{2}\left(M\right)\geq \cdots \geq 0.$$

Different functors answer different questions: D2KE asks for a PSD Hilbertian lift of the distance, SFM asks for an Occam-weighted feature mixture, and heat kernels ask for diffusion regularity. The regime classification below applies to any such functor once fixed.

**Definition 10** (Spectral regime of a kernelized mm-space)**.**  *Fix a kernelization functor $M\mapsto T_{M}$. We say that M is:*

*1.*  Polynomial *with exponent a>1 if $\lambda_{j}\left(M\right)≍j^{-a}$ (three polynomial exponents appear across the series and should not be conflated. The operator decay a here is Part III’s $\lambda_{i}≍i^{-2s/d}$, i.e., a=2s/d (with a>1 in the RKHS-embedding regime s>d/2). It is distinct from the sorted-envelope exponent 1/α of the ideal-spectrum remark in Section 16, which comes from the program-counting function $N\left(L\right)≍2^{\alpha L}$ and governs how Solomonoff mass distributes* across *length classes rather than the decay of any one fixed operator).*
*2.*  *Plateau/truncated at level m if $\lambda_{1},\ldots ,\lambda_{m}$ are comparable and $\lambda_{m+1}$ is separated by a gap or cutoff.*
*3.*  *Supersmooth if $\lambda_{j}\left(M\right)≲\exp\left(-cj^{\rho }\right)$ for some c,ρ>0.*

Thus the three spectral regimes of Parts III–V are not lost in the mm-space language. They become regimes of the geometry-induced operator $T_{M}$. The advantage is that we can now ask *why* a regime appears: from program-counting growth, from a finite length shell, from diffusion geometry, from redundancy in the feature dictionary, or from a deep pullback.

**Proposition 18** (Eigenvalue transfer through stable kernelization)**.**  *Let M and N be finite mm-spaces and let κ be a kernelization functor satisfying a transported Hilbert–Schmidt stability bound*

$$∥T_{M}-T_{N}^{\pi }{∥}_{\mathrm{HS}}\leq C\;d_{\mathrm{GW},2}\left(M,N\right)$$

*for an optimal or near-optimal coupling π, and assume the transport preserves the spectrum $\lambda_{j}\left(T_{N}^{\pi }\right)=\lambda_{j}\left(T_{N}\right)$ for all j (automatic when π is induced by a measure-preserving bijection and an additional hypothesis on the kernelization functor for soft couplings, otherwise the conclusion holds with $\lambda_{j}\left(T_{N}^{\pi }\right)$ in place of $\lambda_{j}\left(N\right)$). Then,*

$${\left(\sum_{j\geq 1}{\left|\lambda_{j}\left(M\right)-\lambda_{j}\left(N\right)\right|}^{2}\right)}^{1/2}\leq C\;d_{\mathrm{GW},2}\left(M,N\right).$$

*Consequently, every* finite-resolution *spectral feature separated from its competitors by more than $2C\;d_{\mathrm{GW},2}\left(M,N\right)$ is stable under the perturbation: eigenvalue clusters, finite plateaux, cutoffs, and effective ranks at thresholds away from the spectrum. This is not an asymptotic regime-inheritance statement. Asymptotic regime labels require a tail condition in addition to Hilbert–Schmidt closeness. For example, a polynomial law $\lambda_{j}\left(M\right)≍j^{-a}$ transfers to N with the same exponent if*

$$|\lambda_{j}\left(M\right)-\lambda_{j}\left(N\right)|=o\left(j^{-a}\right),\;j\to \infty ,$$

*and analogous relative-tail bounds transfer super-smooth decay. GW-close kernelizations preserve only the spectral information visible above the perturbation scale; full tail regimes transfer only under tail-stable kernelization.*

**Proof sketch.**  After transporting $T_{N}$ to the support of M by π, the claimed inequality is the Hoffman–Wielandt/Weyl bound for compact self-adjoint Hilbert–Schmidt operators. The D2KE finite stability result of Section 11 is one instance of the assumed transported stability bound. For finite spaces a common realization is explicit: on $L^{2}\left(\pi \right)$, lift the first kernel to ${\tilde{k}}_{M}\left(\left(x,y\right),\left(x^{\prime },y^{\prime }\right)\right)=k_{M}\left(x,x^{\prime }\right)$, and the second similarly using $y,y^{\prime }$. The coordinate pullbacks are isometries because π has the prescribed marginals; each lifted operator has the original nonzero spectrum, with zeros added. Their Hilbert–Schmidt difference is the coupled kernel discrepancy. Arbitrary conditional averaging along a soft coupling need not preserve spectra, and this construction alone does not establish the task transport in the regression theorem. The finite-resolution statement follows because a perturbation of size ε cannot close a spectral gap larger than 2ε. The final assertion is separate: an $\ell^{2}$ perturbation of the eigenvalue sequence alone does not determine the tail exponent, so asymptotic transfer must be assumed or proved as a relative tail bound for the chosen kernelization functor.    □

### 17.2. Recovering Parts III–VI

The kernel constructions in Parts III–VI reappear in a fixed order once the three maps are separated:

algorithmicdatageometry⟶selectedmm-space⟶kernel/operator⟶learningtheory.

Parts I–II live at the first two arrows: MDL/code length chooses among computable descriptions, while KC/NCD/CKC/AMI provide the empirical algorithmic distances and the KC/D2KE kernel recovered in Proposition 8. Parts III–VI live after kernelization. Table 12 gives the recovery map before the details.

**Part III: D2KE and the CSZ-style trichotomy.** Part III begins with algorithmic distances and asks how to obtain a PSD kernel with a spectrum that falls into one of the complexity regimes. In the present notation, the route is

$${\hat{S}}_{n}=\left(X_{n},{\hat{d}}_{\mathrm{Sol}},{\hat{\mu }}_{n}\right)\;⟶\;\hat{M}\;⟶\;T_{D2\mathrm{KE},\hat{M}}.$$

The Part III regimes are precisely the regimes of $T_{D2\mathrm{KE},\hat{M}}$. Polynomial spectra correspond to geometries for which metric entropy or program-counting function grows polynomially across resolution; plateau spectra correspond to finite length shells or finite effective ranks; and supersmooth spectra correspond to analytic/diffusive geometries for which the kernelization kills high modes exponentially. Thus, D2KE is not merely a repair of non-PSD distances; it is the functor that turns algorithmic mm-geometry into the Hilbertian object on which the CSZ-style trichotomy lives.

**Part IV: SFM, SKCO, SGP, and SGHS.** Part IV is recovered by changing the *measure* in the same master kernel. Uniform landmark mass gives the KC/D2KE Hilbert space, while Occam mass $d\mu \left(\omega \right)\propto 2^{-\ell (\omega )}$ gives

$$k_{\mathrm{SFM}}\left(z,z^{\prime }\right)=\int \phi_{\omega }\left(z\right)\phi_{\omega }\left(z^{\prime }\right)\;d\mu_{\mathrm{Sol}}\left(\omega \right),$$

that is, the probability-normalized uncentered Solomonoff feature mixture and its integral operator. Under orthogonality or the near-orthogonality/Riesz hypotheses of Theorem 2, the eigenvalues of this operator track $2^{-\ell_{i}}$. This is a second-moment construction from Occam-weighted features; identifying it with the posterior-centered covariance projection requires zero feature mean and matching weights.

**Part V: learning rates and spectral collapse.** Part V studies what happens to learning after the projection to $T_{M}$. In the mm-space language, the central quantity is the effective dimension

$$N_{M}\left(\lambda \right)=\mathrm{tr}\;\left(T_{M}{\left(T_{M}+\lambda I\right)}^{-1}\right)=\sum_{j}\frac{\lambda_{j}\left(M\right)}{\lambda_{j}\left(M\right)+\lambda }.$$

Redundancy inflation is the statement that the chosen geometry/feature dictionary increases $N_{M}\left(\lambda \right)$ without adding predictive structure; degeneracy concentration may instead decrease it. The plateau results of Part V remain exactly the finite-capacity case $N_{M}\left(\lambda \right)\approx m$ below the shelf. The global Solomonoff envelope and the Part V plateau regime are compatible, as the former calibrates mass across length classes and the latter controls finite-resolution learning inside an effective shell.

**Part VI: deep KC and pullback geometries.** Part VI is recovered by allowing the candidate geometry itself to be a learned pullback. A representation $\Phi_{\theta }:X\to Z_{\theta }$ induces

$$d_{\theta }\left(x,x^{\prime }\right)=d_{Z}\left(\Phi_{\theta }\left(x\right),\Phi_{\theta }\left(x^{\prime }\right)\right),\;M_{\theta }=\left(\Phi_{\theta }\left(X\right),d_{Z},\Phi_{\theta \#}{\hat{\mu }}_{n}\right).$$

Solomonoff–Gromov selection chooses the pullback geometry with relational distances matching ${\hat{d}}_{\mathrm{Sol}}$, while the marked objective chooses the simplest pullback that is also predictive. Applying D2KE or SFM after this selection recovers the deep KC/Solomonoff kernels of Part VI.

### 17.3. A Learning Theory Directly on mm-Spaces

The preceding discussion suggests a direct extension of Parts III–V. Let G be a class of candidate mm-spaces, let κ be a kernelization functor, and let $H_{M}$ be the RKHS induced by $\kappa_{M}$. For observations $\left(x_{i},y_{i}\right)$, define

$$\hat{M}\in \underset{M\in G}{arg min}\left[d_{\mathrm{GW},p}^{p}\left({\hat{S}}_{n},M\right)+\lambda \;\mathrm{Code}\left(M\right)+\eta \;\mathrm{TaskLoss}\left(M\right)\right],$$

then learn $f\in H_{\hat{M}}$. The complexity is no longer assigned to a kernel alone; it is now assigned to the geometry plus its kernelization. Under squared loss and Gaussian observation noise, Proposition 10 identifies this downstream KRR estimator with the posterior mean of the SGP having covariance $\kappa_{\hat{M}}$. Thus the learning bounds below apply to the same finite predictor under its deterministic SGHS and probabilistic SGP readings.

**Definition 11** (mm-space effective dimension)**.**  *For a kernelized mm-space M, define*

$$N_{M}\left(\lambda \right)=\sum_{j}\frac{\lambda_{j}\left(M\right)}{\lambda_{j}\left(M\right)+\lambda }.$$

*Define the mm spectral dimension by*

$$\beta_{M}=\inf\left\{\beta \geq 0:N_{M}\left(\lambda \right)=O\left(\lambda^{-\beta }\right)\;\mathrm{as}\;\lambda ↓0\right\}.$$

*The infimum need not be attained. Logarithmic/supersmooth growth has dimension zero but can have unbounded effective dimension; its logarithmic factors must remain in rate calculations. If $\lambda_{j}≍j^{-a}$, a>1, then $\beta_{M}=1/a$.*

Capacity is now two-layered. The *inner* capacity is the effective dimension $N_{M}\left(\lambda \right)$ of the selected geometry; this is the usual CSZ/Caponnetto–De Vito quantity after kernelization [83,84]. The *outer* capacity is the complexity of the geometry class G itself, measured either by a Kraft code Code(M) in a countable class or by metric entropy of $\left(G,d_{\mathrm{GW}}\right)$ in a continuous class. The MDL term in the Solomonoff–Gromov objective is not cosmetic, it is the outer-capacity regularizer.

**Theorem 5** (Finite computable-class oracle bound)**.**  *Let $A_{1},\ldots ,A_{N}$ be a finite list of complete computable mm-model candidates. Candidate $A_{j}$ includes its mm-space, a fixed GW distortion $D_{j}\geq 0$, its fitted predictor $f_{j}$, and a prefix code length $L_{j}$ satisfying $\sum_{j}2^{-L_{j}}\leq 1$. Assume the candidates, codes, and predictors are fixed independently of an iid validation sample V of size $n_{\mathrm{val}}$, drawn from the distribution defining $R_{j}$. For a bounded loss ℓ∈[0,B], write*

$$R_{j}=E\left[\ell \left(f_{j}\left(X\right),Y\right)\right],\;{\hat{R}}_{V,j}=\frac{1}{n_{\mathrm{val}}}\sum_{(x,y)\in V}\ell \left(f_{j}\left(x\right),y\right),$$

*and define*

$$q_{j}\left(\delta \right)=B\sqrt{\frac{L_{j}\ln2+\log\left(2/\delta \right)}{2n_{\mathrm{val}}}}.$$

*Let*

$$\hat{j}\in \underset{1\leq j\leq N}{arg min}\left[{\hat{R}}_{V,j}+\gamma D_{j}+\lambda L_{j}+q_{j}\left(\delta \right)\right].$$

*Then, with probability at least 1−δ,*

$$R_{\hat{j}}+\gamma D_{\hat{j}}+\lambda L_{\hat{j}}\leq \underset{1\leq j\leq N}{\inf}\left[R_{j}+\gamma D_{j}+\lambda L_{j}+2q_{j}\left(\delta \right)\right].$$

*The same statement holds for a countable coded class by replacing the finite list with any prefix-code family and interpreting the infimum over candidates with finite code length; since the penalized argmin need not be attained over an infinite list, $\hat{j}$ is then taken to be any ϵ-minimizer and ϵ is added to the right-hand side. For sub-Gaussian losses, replace B by the corresponding sub-Gaussian scale up to the usual absolute constant.*

**Proof.**  Hoeffding’s inequality [85] with failure probability $\delta_{j}=\delta 2^{-L_{j}}$ gives

$$|{\hat{R}}_{V,j}-R_{j}|\leq q_{j}\left(\delta \right)$$

for all candidates simultaneously, since $\sum_{j}\delta_{j}\leq \delta$. On this event,

$$R_{\hat{j}}+\gamma D_{\hat{j}}+\lambda L_{\hat{j}}\leq {\hat{R}}_{V,\hat{j}}+q_{\hat{j}}+\gamma D_{\hat{j}}+\lambda L_{\hat{j}}.$$

The definition of $\hat{j}$ bounds the right-hand side by the same validation objective for any *j*, and the reverse concentration inequality converts ${\hat{R}}_{V,j}$ back to $R_{j}$, producing the displayed oracle inequality.    □

**Remark 23** (Role of the finite theorem)**.**  *This theorem is intentionally modest. It does not prove that a compressor has found the true Solomonoff posterior geometry, nor does it give a uniform theorem over arbitrary continuous geometry classes. Once a coded mm-model list and an independent validation protocol are fixed, it supplies the standard finite-sample Occam bound. The distortion $D_{j}$ is only a non-negative score fixed before validation; all metric–measure content lies in how that score and the induced predictor are constructed. The conditional results below identify the additional transport, stability, and concentration assumptions needed for a geometry-level oracle statement.*

**Theorem 6** (Conditional expected-risk template on a selected mm-space)**.**  *Let $M_{★}$ be an oracle population geometry and let $\hat{M}=M_{\hat{\theta }}$ be selected using data independent of the n-sample subsequently used for regression (equivalently, condition on a selected geometry obtained from an independent split). Suppose the kernels are bounded by 1, the regression noise is sub-Gaussian with scale σ, and the oracle covariance is a positive contraction. Assume the source condition*

$$f_{★}=T_{M_{★}}^{s}g,\;0<s\leq 1,\;{∥g∥}_{L^{2}\left(\mu_{★}\right)}\leq R.$$

*The restriction s≤1 is the qualification-one range of ordinary Tikhonov/KRR; for s>1 the bias saturates at the s=1 rate unless a higher-qualification regularizer is used.*

*To compare operators on different $L^{2}$ spaces without assuming equal Hilbert-space dimension, assume that the transport construction used in this statement supplies an isometric embedding*

$$U_{\pi }:L^{2}\left(\hat{\mu }\right)⟶L^{2}\left(\mu_{★}\right),\;U_{\pi }^{*}U_{\pi }=I,$$

and write $P_{\pi }=U_{\pi }U_{\pi }^{*}$ for the projection onto its range. Assume that the population problem is supported on that transported range,

$$P_{\pi }f_{★}=f_{★},\;T_{M_{★}}=P_{\pi }T_{M_{★}}P_{\pi },$$

*and define*

$$T_{\hat{M}}^{\pi }:=U_{\pi }T_{\hat{M}}U_{\pi }^{*}.$$

*A measure-preserving bijection gives the special surjective (unitary) case. A general soft GW coupling does not canonically supply even this isometric transport; using one is an additional construction, and is not inferred from small GW alone. If the two range-support conditions fail, then the same argument controls the projected population problem and the unprojected risk acquires an additional projection/approximation residual. Assume that the kernelization is operator-Lipschitz for this transport:*

$$∥T_{\hat{M}}^{\pi }-T_{M_{★}}{∥}_{\mathrm{op}}\leq L_{\kappa }\;d_{\mathrm{GW},2}\left(\hat{M},M_{★}\right).$$

*Also, impose the following additional conditional KRR sample-error estimate around the population ridge solution,*

$$E\;\left[∥{\hat{f}}_{\lambda }-A_{\lambda }\left(T_{\hat{M}}^{\pi }\right)f_{★}{∥}_{L^{2}\left(\mu_{★}\right)}^{2}\;|\;\hat{M}\right]\leq C_{\mathrm{sam}}\frac{\sigma^{2}N_{\hat{M}}\left(\lambda \right)}{n},\;A_{\lambda }\left(T\right)=T{\left(T+\lambda I\right)}^{-1},$$

*as an explicit hypothesis on the regression experiment. It does not follow merely from bounded kernels, finite rank, or independent splitting: random-design fluctuations can remain even with noiseless labels. For the trace-stability conclusion below, assume the ridge-scale trace stability*

$$\left|\mathrm{tr}\left(T_{\hat{M}}^{\pi }{\left(T_{\hat{M}}^{\pi }+\lambda I\right)}^{-1}\right)-\mathrm{tr}\left(T_{M_{★}}{\left(T_{M_{★}}+\lambda I\right)}^{-1}\right)\right|\leq L_{N,\lambda }\;d_{\mathrm{GW},2}\left(\hat{M},M_{★}\right).$$

*This trace/effective-dimension stability is a real assumption: operator-norm closeness alone does not prevent many small eigenvalues from inflating the variance. Then, for squared-loss prediction risk and expectation over the independent regression sample and its noise,*

$$E\left[E\left({\hat{f}}_{\lambda }\right)-E\left(f_{★}\right)|\hat{M}\right]\;\leq \;C\left[R^{2}\lambda^{2s}+\frac{\sigma^{2}N_{M_{★}}\left(\lambda \right)}{n}+\frac{\sigma^{2}L_{N,\lambda }}{n}d_{\mathrm{GW},2}\left(\hat{M},M_{★}\right)+R^{2}\frac{L_{\kappa }^{2}}{\lambda^{2}}d_{\mathrm{GW},2}^{2}\left(\hat{M},M_{★}\right)\right],$$

*Alternatively, if only $\mathrm{rank}T_{\hat{M}}\leq r$ is assumed in place of trace stability, the conclusion is*

$$E\left[E\left({\hat{f}}_{\lambda }\right)-E\left(f_{★}\right)|\hat{M}\right]\leq C\left[R^{2}\lambda^{2s}+\frac{\sigma^{2}r}{n}+\frac{R^{2}L_{\kappa }^{2}}{\lambda^{2}}d_{\mathrm{GW},2}^{2}\left(\hat{M},M_{★}\right)\right].$$

*Both branches retain the explicit sample-error hypothesis. Here C depends only on the constant in the stated sample-error estimate and not on n, λ, or the selected geometry. Thus, the geometry error must be optimized jointly with the ridge parameter. By Equation (15), one may substitute*

$$\delta_{\mathrm{geom}}:=\delta_{\mathrm{trunc}}\left(L\right)+\delta_{\mathrm{comp}}\left(C,L\right)+\delta_{\mathrm{samp}}\left(n\right)+\delta_{\mathrm{app}}\left(G\right)+\delta_{\mathrm{opt}}$$

*for the GW discrepancy only if these terms bound a chain between the selected and oracle geometries; here $\delta_{\mathrm{opt}}$ must bound selection displacement, not merely objective excess. If the geometry is random and an unconditional expectation is desired, average its discrepancy and squared discrepancy separately. A bound on $E\delta_{\mathrm{geom}}$ does not imply a bound on $E\delta_{\mathrm{geom}}^{2}$ by its square. Under the p=2 box-dimension hypothesis of Theorem 4, $E\;\delta_{\mathrm{samp}}\left(n\right)≲n^{-1/(2s^{\prime })}$ for every $s^{\prime }>\max\left(d_{\mathrm{box}},2\right)$, with a space-dependent constant. If $N_{M_{★}}\left(\lambda \right)≲\lambda^{-\beta }$, the fixed-geometry choice $\lambda_{n}≍n^{-1/(2s+\beta )}$ gives*

$$n^{-2s/(2s+\beta )}$$

*provided that both geometry contributions are of that order; in particular,*

$${\left(\frac{\delta_{\mathrm{geom}}}{\lambda_{n}}\right)}^{2}=O\;\left(n^{-2s/(2s+\beta )}\right).$$

*The trace contribution additionally requires $L_{N,\lambda_{n}}\delta_{\mathrm{geom}}/n$ to be no larger than the target rate. Otherwise, λ must be chosen by optimizing the full displayed bound. The empirical-GW rate in Theorem 4 is an upper bound, not a matching lower bound. It can verify the displayed requirement when it is sufficiently fast, but a slower upper bound cannot be reversed to prove that the fixed-geometry rate is impossible; that conclusion would require a lower bound or a sharper geometry estimate.*

**Proof.**  For 0<s≤1, spectral calculus gives the qualification-one bias bound

$$∥\left(I-A_{\lambda }\left(T_{M_{★}}\right)\right)f_{★}∥\leq R\lambda^{s}.$$

The resolvent identity yields

$$∥A_{\lambda }\left(T\right)-A_{\lambda }{\left(S\right)∥}_{\mathrm{op}}\leq \frac{{∥T-S∥}_{\mathrm{op}}}{\lambda },$$

and $∥f_{★}∥\leq R$ because $T_{M_{★}}$ is a contraction. Therefore, the operator-Lipschitz hypothesis bounds the squared population-filter perturbation by $R^{2}L_{\kappa }^{2}d_{\mathrm{GW},2}^{2}/\lambda^{2}$. The assumed sample-error estimate supplies the variance term, and trace stability bounds $N_{\hat{M}}\left(\lambda \right)$ by $N_{M_{★}}\left(\lambda \right)+L_{N,\lambda }d_{\mathrm{GW},2}$. Applying ${∥a+b+c∥}^{2}\leq {3(∥a∥}^{2}+{∥b∥}^{2}+{∥c∥}^{2})$ proves the trace-stability result. In the finite-rank branch, use $N_{\hat{M}}\left(\lambda \right)\leq r$ directly instead. The final substitutions use Equation (15) and Theorem 4.    □

**Remark 24** (Finite rank bounds the effective dimension)**.**  *Suppose $G_{r}$ is a class for which the kernelizations all factor through a common r-dimensional feature space*

$$k_{M}\left(x,x^{\prime }\right)={\langle \phi_{M}\left(x\right),\phi_{M}\left(x^{\prime }\right)\rangle }_{R^{r}},\;∥\phi_{M}\left(x\right)∥\leq 1.$$

*Then, every associated covariance operator has rank at most r; hence,*

$$N_{M}\left(\lambda \right)=\mathrm{tr}\left(T_{M}{\left(T_{M}+\lambda I\right)}^{-1}\right)\leq r$$

*for every λ>0. This bounds the effective-dimension factor conditional on a valid sample-error estimate. It does not establish that estimate. For example, let X be uniform on {0,1}, $k\left(x,x^{\prime }\right)=xx^{\prime }$, $f_{★}\left(x\right)=x$, n=1, λ=1, and noise variance zero. The population ridge solution is x/3, while the empirical solution is either zero or x/2; their expected squared $L^{2}$ difference is 5/144>0. Thus finite rank alone cannot justify a variance-only sample bound.*

**Corollary 2** (GW distortion drives risk on finite-rank D2KE classes)**.**  *Let $G_{r}$ be a class of landmark/D2KE kernelized metric–measure spaces with kernels that all factor through a common r-dimensional feature space with $∥\phi_{M}\left(x\right)∥\leq 1$. Let $M_{★}\in G_{r}$ be an oracle geometry satisfying the source condition in Theorem 6, with sub-Gaussian noise of scale σ. Assume that the D2KE kernelization is operator-Lipschitz along GW couplings on this class,*

$$∥T_{\hat{M}}^{\pi }-T_{M_{★}}{∥}_{\mathrm{op}}\leq L_{\kappa }d_{\mathrm{GW},2}\left(\hat{M},M_{★}\right),$$

*a condition supported at the finite kernel level by Proposition 13. Additionally, assume the independent split, isometric transport, range support, and conditional sample error conditions of Theorem 6 and suppose that*

$$d_{\mathrm{GW},2}\left(\hat{M},M_{★}\right)\leq \varepsilon .$$

*Then, kernel ridge regression with the induced kernel $\kappa_{\hat{M}}$ and ridge parameter λ satisfies*

$$E\left[E\left({\hat{f}}_{\lambda }\right)-E\left(f_{★}\right)|\hat{M}\right]\;\leq \;C\left[R^{2}\lambda^{2s}+\frac{\sigma^{2}r}{n}+R^{2}L_{\kappa }^{2}\frac{\varepsilon^{2}}{\lambda^{2}}\right].$$

*Thus, on this finite-rank D2KE class, GW distortion enters the prediction guarantee through the induced kernel operator, not merely as an additive validation score, and the bound reduces to the usual finite-rank KRR rate as ε→0 when λ is chosen in the fixed-geometry regime.*

**Proof.**  The finite-rank assumption gives $N_{\hat{M}}\left(\lambda \right),N_{M_{★}}\left(\lambda \right)\leq r$, so no separate trace-stability term is needed. The operator-Lipschitz assumption and $d_{\mathrm{GW},2}\left(\hat{M},M_{★}\right)\leq \varepsilon$ give $∥T_{\hat{M}}^{\pi }-T_{M_{★}}{∥}_{\mathrm{op}}\leq L_{\kappa }\varepsilon$. Substitution in Theorem 6 gives the result.    □

This theorem is the mm-space analogue of the CSZ/Parts III–V learning theory. The three regimes give the familiar consequences:

$$\begin{array}{ccc} \mathrm{polynomial}: & N\left(\lambda \right)≍\lambda^{-1/a} & \Rightarrow \;n^{-2s/(2s+1/a)},\\ \mathrm{plateau}: & N(\lambda )\approx m & \Rightarrow \;\mathrm{parametric}\;m/n\;\mathrm{variance}\;\mathrm{below}\;\mathrm{the}\;\mathrm{cutoff},\\ \mathrm{super}\text{-}\mathrm{smooth}: & N\left(\lambda \right)≲\log{\left(1/\lambda \right)}^{q} & \Rightarrow \;\mathrm{near}\text{-}\mathrm{parametric}\;\mathrm{rates}\;\mathrm{up}\;\mathrm{to}\;\mathrm{logarithms}. \end{array}$$

What this paper adds is the selection layer: the learner may choose the geometry for which the induced $N_{M}$, code length, and task loss best match the empirical Solomonoff structure.

### 17.4. How the Unification and Oracle Results Fit Together

Theorem 1 and the error/oracle results answer different questions. The unification theorem begins with a fixed distance, landmark measure, bandwidth, and D2KE feature family. It shows how empirical/uniform and Occam landmark measures recover the two earlier kernel constructions; its spectral clause retains the orthogonality or perturbative hypotheses of Theorem 2. It is *selection-free once those ingredients are fixed*, not a claim that D2KE is the unique category-independent kernelization.

The error decomposition and the finite and countable oracle statements instead concern how a geometry or induced predictor is chosen from data. They do not make the chosen representation canonical. Their conclusions require additional bridges: a declared candidate class and code, independent validation or another generalization device, a marked or predictive selection criterion for supervised tasks, and stability of the induced kernel learner. These assumptions are not consequences of Theorem 1.

The two layers compose only in the forward direction

$$\begin{array}{c} \mathrm{selected}\;\mathrm{geometry}⟶\mathrm{fixed}\;D2\mathrm{KE}/\mathrm{SFM}\;\mathrm{representation}\\ ⟶\mathrm{predictor}\;\mathrm{and}\;\mathrm{conditional}\;\mathrm{risk}\;\mathrm{control} \end{array}$$

The unification theorem supplies the middle map; the selection theorems control the first and last arrows under their separate hypotheses. Neither layer implies the other. The cellular-automaton and SMS prediction studies (E10–E11) expose a further prerequisite that an oracle inequality cannot supply: competitiveness is only relative to the included candidate family. Even if every quotient in that family destroys task-relevant structure, an internally valid oracle comparison can still select a predictively poor model.

### 17.5. Oracle Inequalities over Geometries

The preceding theorem is the fixed-oracle slice of the mm-space theory. The full selection statement has one more term: the price of searching over geometries. For countable geometry classes, this term is controlled by the same Kraft/MDL mechanism as the mixture dominance bound of Proposition 15; for continuous classes, it requires uniform predictive generalization control for the induced data-dependent learners.

The assumptions form the explicit ladder in Table 13. The final oracle theorem is not a consequence of the definition of Solomonoff–Gromov selection alone. Each row adds a separate bridge; if that bridge is unavailable, the valid conclusion stops at the preceding row.

**Theorem 7** (Conditional countable-geometry validation oracle)**.**  *Let G be a countable class of candidate geometries and let* Λ *be a fixed singleton or a countable regularization grid. For a=(M,λ), assign nonnegative code lengths*

$$\mathrm{Code}\left(a\right)=\mathrm{Code}\left(M\right)+{\mathrm{Code}}_{\Lambda }\left(\lambda \right),\;\sum_{a}2^{-\mathrm{Code}(a)}\leq 1,$$

*with ${\mathrm{Code}}_{\Lambda }=0$ for a fixed singleton. Condition on construction data that fix the candidates and their codes. Fit each predictor ${\hat{f}}_{a}$ on regression data, independently of an iid validation sample of size $n_{\mathrm{val}}$. All predictors must be evaluated on the same observed input space through declared measurable maps. Suppose the validation loss lies in [0,B]. Define*

$$q_{a}=B\sqrt{\frac{\mathrm{Code}(a)\ln2+\log(4/\delta )}{2n_{\mathrm{val}}}},\;0<\delta <1.$$

*Let $G_{a}\geq 0$ be a penalty fixed independently of validation, and let $\hat{a}$ be an $\varepsilon_{\mathrm{opt}}$-minimizer of*

$${\hat{E}}_{V}\left({\hat{f}}_{a}\right)+q_{a}+G_{a}.$$

*Then, with probability at least 1−δ/2,*

$$E\left({\hat{f}}_{\hat{a}}\right)\leq \underset{a}{\inf}\left\{E\left({\hat{f}}_{a}\right)+2q_{a}+G_{a}\right\}+\varepsilon_{\mathrm{opt}}.$$

*If, additionally, candidate bounds satisfy simultaneously with probability at least 1−δ/2*

$$E\left({\hat{f}}_{a}\right)-E\left(f_{★}\right)\leq H_{a},$$

*then with probability at least 1−δ,*

$$E\left({\hat{f}}_{\hat{a}}\right)-E\left(f_{★}\right)\leq \underset{a}{\inf}\left\{H_{a}+2q_{a}+G_{a}\right\}+\varepsilon_{\mathrm{opt}}.$$

*For the KRR interpretation, a permissible* additional *candidate-bound assumption is*

$$H_{a}=C\left[R_{M}^{2}\lambda^{2s_{M}}+\frac{\sigma^{2}N_{M}\left(\lambda \right)}{n}+G_{a}^{\mathrm{geom}}\right]+q_{a}^{\mathrm{KRR}},$$

*where $\Delta_{M}$ is a valid bound on the relevant transported geometry discrepancy and*

$$G_{a}^{\mathrm{geom}}=\frac{\sigma^{2}L_{N,\lambda }}{n}\Delta_{M}+\frac{R_{M}^{2}L_{\kappa }^{2}}{\lambda^{2}}\Delta_{M}^{2}.$$

*This high-probability assumption must be proved for the specified regression experiment, including the transport, source, sample-error, and trace hypotheses; it does not follow from the expected-risk template alone. In its finite-rank alternative, replace the effective-dimension and trace terms by $\sigma^{2}r/n$. Choosing $G_{a}$ as a computable upper bound on $G_{a}^{\mathrm{geom}}$ gives a risk-scaled geometry penalty. Any extra MDL penalty included in the selection objective remains in $G_{a}$ and hence in the comparator bound. For squared loss, bounded validation loss requires bounded responses and suitably bounded or clipped predictors; a uniform sub-Gaussian loss assumption permits the corresponding concentration radius instead.*

**Proof.**  Condition on all construction and regression data. For each candidate, Hoeffding’s inequality [85] gives

$$\mathrm{Pr}\{|{\hat{E}}_{V}\left({\hat{f}}_{a}\right)-E\left({\hat{f}}_{a}\right)\left|>q_{a}\right\}\leq \left(\delta /2\right)2^{-\mathrm{Code}(a)}.$$

Summing over the coded class bounds the failure probability by δ/2. On the complementary event, approximate minimization and $G_{\hat{a}}\geq 0$ give, for every *a*,

$$\begin{array}{cc} E({\hat{f}}_{\hat{a}}) & \leq {\hat{E}}_{V}\left({\hat{f}}_{\hat{a}}\right)+q_{\hat{a}}+G_{\hat{a}}\\ & \leq {\hat{E}}_{V}\left({\hat{f}}_{a}\right)+q_{a}+G_{a}+\varepsilon_{\mathrm{opt}}\\ & \leq E\left({\hat{f}}_{a}\right)+2q_{a}+G_{a}+\varepsilon_{\mathrm{opt}}. \end{array}$$

Taking the infimum proves the first assertion. Intersect with the assumed uniform candidate-risk event and substitute $H_{a}$ to prove the second. A code-weighted union bound on separately proved candidate tails is one way to obtain that event. This is a validation-selector result; the sequential mixture redundancy bound in Proposition 15 concerns a different predictor and loss guarantee. Related concentration and coding approaches include U-statistics, bounded differences, and PAC-Bayes [86,87,88].    □

**Remark 25** (Countability and validation are substantive)**.**  *The countability and validation split assumptions are not cosmetic. The theorem does not assert a uniform oracle inequality over arbitrary continuous geometry classes. A continuous parameter family requires either discretization by a coded net, a PAC-Bayes prior over parameters, or a uniform predictive generalization theorem. Establishing such a theorem for broad continuous geometry classes is part of the open statistical theory of Solomonoff–Gromov approximation.*

**Remark 26** (Unmarked GW is not a predictive oracle bound)**.**  *The theorem deliberately assumes a marked or predictive selection objective. Pure unmarked Solomonoff–Gromov selection can certify that the prior geometry or covariance geometry is relationally close to the empirical Solomonoff space, but cannot by itself identify which labels or function values attach to which points. This is the same loss recorded in Proposition 17: GW is isometry-invariant and does not identify external task marks. Predictive oracle inequalities require either marked GW, an empirical risk term, or, for a mixture predictor rather than a selected KRR model, the separate sequential log-loss bound of Proposition 15.*

**Remark 27** (Uniform geometric control and predictive complexity)**.**  *For every class G, even an uncountable one, the reverse triangle inequality gives*

$$\underset{M\in G}{\sup}\left|d_{\mathrm{GW},p}\left(\hat{S},M\right)-d_{\mathrm{GW},p}\left(S,M\right)\right|\leq d_{\mathrm{GW},p}\left(\hat{S},S\right).$$

*For powered distances uniformly bounded by R, multiply the right side by $pR^{p-1}$. Thus candidate-class entropy is not needed for this geometric comparison. The unresolved outer-capacity problem is uniform predictive-loss control when continuous geometry selection also changes the learned function class and its task alignment. Entropy or Rademacher complexity can enter that distinct generalization problem.*

## 18. Ultrametric Solomonoff Geometry

### 18.1. Prefix Trees

The natural geometry of prefix-free programs is a tree, and trees correspond to ultrametric spaces in a precise sense [89]; ultrametric GW and Wasserstein geometries provide useful comparison points [90,91]. Let $\Omega \subset {\left\{0,1\right\}}^{*}$ be a prefix-free code set. Define the prefix distance

$$d_{\mathrm{pref}}\left(p,q\right)=\left\{\begin{array}{cc} 0, & p=q,\\ 2^{-|p\wedge q|}, & p\neq q, \end{array}\right.$$

where p∧q is the longest common prefix of *p* and *q*. This is an ultrametric:

$$d_{\mathrm{pref}}\left(p,r\right)\leq \max\left\{d_{\mathrm{pref}}\left(p,q\right),d_{\mathrm{pref}}\left(q,r\right)\right\}.$$

For a nonempty code set, normalize the weights by $W=\sum_{p\in \Omega }2^{-|p|}\in \left(0,1\right]$. The metric completion, carrying the push-forward of these probability masses, is an ultrametric mm-space. Completion matters for infinite code sets, whose Cauchy sequences can converge to infinite strings.

The same tree carries a native positive semidefinite prefix kernel. For strings x,y, set

(16) $$k_{\mathrm{Sol},\mathrm{pref}}\left(x,y\right)=\sum_{u⪯x,y}2^{-|u|},$$

where u⪯x,y means that *u* is a common prefix of both strings. This is the inner product of shared-prefix indicator features weighted by Occam mass. More generally, one may use $2^{-K(u)}$, whose total sum is finite by choosing shortest prefix descriptions. For M(u), either restrict depth or insert a summable depth factor, for example $2^{-|u|}M\left(u\right)$, to ensure finite diagonal sums on infinite strings. Thus, the ultrametric presentation has its own Solomonoff kernel before any Euclidean or Gaussian projection is introduced.

### 18.2. Why Ultrametrics Matter

Many algorithmic data sets are hierarchical: files have reusable subroutines, strings share prefixes, programs share modules, grammars share production trees. While a Euclidean geometry often hides this hierarchy, an ultrametric geometry exposes it.

This preference is sharper than interpretability. Among finite metrics the Schoenberg gate of Definition 7—$d^{2}$ conditionally negative definite—is exactly what decides $L^{2}$-embeddability, and *ultrametrics always pass it* (every separable ultrametric space embeds isometrically into $\ell_{2}$ [92]; see also [93]), whereas generic graph and even tree metrics do not. The Hamming-distance star $d_{H}$ on {000,001,010,100} is already non-Euclidean; it is the very witness in [76] (Thm. 33). Both ultrametric and Euclidean candidates admit isometric Hilbert embeddings, whereas general graph shortest-path metrics need not. A separate kernelization can still change the original distances.

The Solomonoff–Gromov framework makes this testable. Fit both a Euclidean candidate and an ultrametric tree candidate to ${\hat{S}}_{n}$. If the ultrametric candidate has much smaller GW + MDL score, then the data are better described by hierarchical program structure than by smooth Euclidean variation.

### 18.3. Tree-Aligned Couplings

For a truncated prefix tree, an optimal or near-optimal GW coupling has a natural interpretation: it aligns clusters of data points with subtrees. This gives a geometric form of algorithmic clustering, in the spirit of the ultrametric characterization of hierarchical clustering [94]. Instead of clustering by Euclidean proximity or kernel similarity, one clusters by common explanatory prefixes.

**Remark 28** (Identifiability in tree-aligned coupling)**.**  *An exact isometry between finite ultrametric probability spaces induces a zero-distortion GW coupling. Its named branches are identifiable only modulo measure-preserving tree automorphisms. Correct depth alone is insufficient: repeated subtree shapes or equal masses can produce indistinguishable alignments. Recovering specific prefix clusters from perturbed data requires a separation condition and a task-relevant correspondence in addition to compressor and sampling control. Quantitative recovery under such hypotheses remains open.*

## 19. Algorithms

### 19.1. Basic Finite Algorithm

Algorithm 1 states the basic finite selection procedure.
**Algorithm 1** Finite Solomonoff–Gromov model selectionInput: data $x_{1},\ldots ,x_{n}$, and labels if a marked/predictive objective is used; compressor or algorithmic distance estimator; candidate geometry class G; penalties λ,η.  1.For predictive selection, reserve an iid validation split $I_{\mathrm{val}}$ independent of all construction and fitting data $I_{\mathrm{tr}}$. To invoke the conditional regression template as well, further split $I_{\mathrm{tr}}$ into independent geometry-construction and regression samples. Fix candidate codes and any regularization grid without validation data. Pure geometric selection uses its separately declared GW + MDL objective.  2.Compute the empirical algorithmic distance matrix on the construction set,$${\hat{D}}_{ij}^{\mathrm{Sol}}={\hat{d}}_{\mathrm{Sol}}\left(x_{i},x_{j}\right),\;i,j\in I_{\mathrm{tr}}.$$  3.Repair or symmetrize the matrix if necessary.  4.Choose an empirical measure ${\hat{\mu }}_{\mathrm{tr}}$, uniform or Occam-weighted, on the construction set.  5.For each candidate θ, compute or sample its distance matrix $D^{\theta }$ and mass vector $\mu_{\theta }$. If marked GW is used, declare and fit its candidate-side mark map $m_{\theta }$ using $I_{\mathrm{tr}}$ only; the marks must exist before the coupling is optimized. In supervised applications, include an uncoarsened source-geometry representation or another declared safe candidate whenever the quotient family has not been shown adequate; the real-data SMS study (E11) shows that adding marks cannot by itself repair a menu that has already discarded the predictive structure.  6.Solve the finite GW or marked GW problem between $\left({\hat{D}}^{\mathrm{Sol}},{\hat{\mu }}_{\mathrm{tr}}\right)$ and $\left(D^{\theta },\mu_{\theta }\right)$, using the already declared $m_{\theta }$ in the marked case. A joint or alternating mark–coupling fit is a different procedure and needs its own training-only objective and convergence analysis.  7.Construct each induced kernel using D2KE, heat kernelization, or SFM, and fit its predictor on the regression data. If only the finite validation comparison is sought, construction and regression may share $I_{\mathrm{tr}}$; invoking Theorem 6 requires their independence.  8.For Theorem 7, score each fitted pair *a* by ${\hat{E}}_{V}\left({\hat{f}}_{a}\right)+q_{a}+G_{a}$, with its stated confidence radius and a nonnegative penalty fixed before validation. A geometry-to-risk conclusion requires the stated candidate-risk bounds and a valid risk-scaled geometry penalty. Retain any extra code or raw-GW score in $G_{a}$ and in the comparator bound. Pure geometric selection instead uses its declared GW + MDL score. Compute validation-to-training-landmark distances only for evaluating the fitted predictor; validation labels do not enter construction or fitting.  9.Select $\hat{\theta }$ minimizing the resulting objective.10.Use the resulting RKHS/GP/SGHS for regression, classification, clustering, density estimation, or posterior inference.

**Cost scale.** For a finite empirical space with *n* data points and a candidate with *m* support points, storing dense distance matrices costs $O(n^{2}+m^{2})$ memory and the coupling costs O(nm). A naive GW objective evaluation uses $O(n^{2}m^{2})$ pair interactions. Entropic/conditional gradient implementations exploit matrix products and typically scale per major iteration, such as $O(n^{2}m+nm^{2})$ plus O(nm) coupling updates, with constants depending on the solver. Landmark approximations with *r* landmarks reduce distance construction/storage toward O((n+m)r) plus the cost of the reduced GW or core-set problem. These are implementation scalings, not new statistical guarantees.

### 19.2. Entropic Approximation

Exact GW is often computationally hard [95]. Entropic regularization has an information-theoretic reading worth stating here: up to an additive constant, the regularizer is the Kullback–Leibler divergence of the coupling from the product of its marginals, i.e., the *mutual information* of the alignment, so entropic GW is distortion minimization under a mutual-information budget, a rate–distortion form of the alignment problem [32,33,81,96]. It replaces the finite problem by

$$\min_{\Pi \in U(\alpha ,\beta )}\frac{1}{2}\sum_{i,i^{\prime },a,a^{\prime }}{\left|D_{ii^{\prime }}^{X}-D_{aa^{\prime }}^{Y}\right|}^{p}\Pi_{ia}\Pi_{i^{\prime }a^{\prime }}+\varepsilon \sum_{i,a}\Pi_{ia}\left(\log\Pi_{ia}-1\right).$$

For fixed marginals, the displayed entropy term equals KL(Π∥α⊗β)−H(α)−H(β)−1, so it has the same minimizers as the centered mutual-information penalty, while its raw objective value differs by a known constant. Here, KL is measured in nats because natural logarithms are used, so ε has the same units as C(Π), namely, the chosen distance raised to the *p*-th power.

**Proposition 19** (Finite entropic-bias bound)**.**  *Let $\alpha \in \Delta_{n}$ and $\beta \in \Delta_{m}$, let C(Π) denote the unregularized finite GW distortion including the 1/2 convention of Definition 2, and define*

$$C_{0}=\min_{\Pi \in U(\alpha ,\beta )}C\left(\Pi \right),\;C_{\varepsilon }=\min_{\Pi \in U(\alpha ,\beta )}\left\{C(\Pi )+\varepsilon \mathrm{KL}(\Pi ∥\alpha ⊗\beta )\right\}.$$

*With the usual conventions on zero-mass entries,*

$$0\leq C_{\varepsilon }-C_{0}\leq \varepsilon \min\left\{H\left(\alpha \right),H\left(\beta \right)\right\}\leq \varepsilon \log\min\left\{n,m\right\}.$$

*Consequently, if $d_{0}=C_{0}^{1/p}$ and $d_{\varepsilon }=C_{\varepsilon }^{1/p}$, then*

$$0\leq d_{\varepsilon }-d_{0}\leq {\left(\varepsilon \min\{H(\alpha ),H(\beta )\}\right)}^{1/p}.$$

**Proof.**  Non-negativity of relative entropy gives the lower bound. If $\Pi_{0}$ minimizes *C*, then

$$C_{\varepsilon }\leq C\left(\Pi_{0}\right)+\varepsilon \mathrm{KL}\left(\Pi_{0}∥\alpha ⊗\beta \right).$$

The relative entropy is the mutual information of a pair with marginals α,β, and hence is at most min{H(α),H(β)}, which is at most logmin{n,m}. The final display follows from ${\left(a+b\right)}^{1/p}\leq a^{1/p}+b^{1/p}$ for p≥1. □

The proposition bounds the regularization bias of the centered finite objective; it does not certify convergence of a particular non-convex numerical run. Sinkhorn-like or inexact gradient algorithms and their convergence conditions are studied in [96]. Accordingly, the error budget keeps the numerical solver gap in $\delta_{\mathrm{opt}}$ and the regularization bias in $\delta_{\mathrm{ent}}$, rather than identifying an entropic value with exact GW.

The bound also gives a transparent tuning rule. Writing $H_{\min}=\min\left\{H\left(\alpha \right),H\left(\beta \right)\right\}$, a desired tolerance ρ on the powered objective is guaranteed by $\varepsilon \leq \rho /H_{\min}$, while a tolerance *r* on the unpowered GW distance is guaranteed by $\varepsilon \leq r^{p}/H_{\min}$ (with zero bias when $H_{\min}=0$). In practice, continuation from larger to smaller ε, followed on small instances by an unregularized multi-start check, separates numerical convenience from the regularization bias budget.

### 19.3. Landmarks and Scalable D2KE

For large *n*, compute distances to landmarks rather than all pairwise distances. Let $L=\{\omega_{1},\ldots ,\omega_{m}\}$ be landmark programs or representative data points. Define

$$\Phi \left(x\right)=\left(\sqrt{\mu_{1}}e^{-\gamma {\hat{d}}_{\mathrm{Sol}}\left(x,\omega_{1}\right)},\ldots ,\sqrt{\mu_{m}}e^{-\gamma {\hat{d}}_{\mathrm{Sol}}\left(x,\omega_{m}\right)}\right).$$

Here $\mu_{i}\geq 0$, $\sum_{i}\mu_{i}=1$, and uniform landmarks use $\mu_{i}=1/m$. Inner products give the D2KE kernel for this landmark probability measure; approximation to another reference measure requires a quadrature or sampling argument. For GW itself, the feature map is used in one of two concrete ways: either to form the low-rank surrogate distance

$${\hat{D}}_{ij}^{\left(L\right)}={∥\Phi \left(x_{i}\right)-\Phi \left(x_{j}\right)∥}_{2}$$

and pass this smaller/structured distance representation to a GW solver, or to compute a core-set/landmark coupling first and extend it to the full sample. Thus, the landmark step is not a replacement of GW by ordinary MMD; it is a low-rank or core-set approximation to the distance matrices entering the GW problem. The resulting low-rank error is part of $\delta_{\mathrm{opt}}$ or the empirical distance approximation term, depending on how the landmarks are chosen. The Euclidean feature distance branch is an early Hilbertization, making it a potentially lossy accelerator, not an exact preservation of the category-native dissimilarity. Its distortion should be audited, for example by $\delta_{L}={∥{\hat{D}}^{\left(L\right)}-{\hat{D}}^{\mathrm{Sol}}∥}_{\infty }$, which feeds directly into the distance perturbation stability bound of Proposition 6. When preservation of non-Hilbert relational structure is essential, a core-set that retains the original dissimilarities is the safer approximation branch.

### 19.4. Barycenters

Given several datasets or tasks, one can define their empirical Solomonoff spaces

$${\hat{S}}_{n}^{\left(1\right)},\ldots ,{\hat{S}}_{n}^{\left(T\right)}.$$

A Solomonoff–Gromov barycenter is

$$\bar{S}\in \underset{M}{arg min}\sum_{t=1}^{T}w_{t}d_{\mathrm{GW},p}^{p}\left(M,{\hat{S}}_{n}^{\left(t\right)}\right)+\lambda \mathrm{Code}\left(M\right).$$

This barycenter is a shared algorithmic geometry across tasks, and could serve as a meta-learned prior.

## 20. Empirical Certification

A kernel with decaying eigenvalues is not automatically Solomonoff-like; many ordinary priors penalize complexity. The Solomonoff–Gromov framework adds an empirical certification criterion.

### 20.1. Relational Solomonoff Preservation

**Definition 12** (Relational Solomonoff preservation)**.**  *A candidate geometry $M_{\theta }$ preserves empirical Solomonoff structure at tolerance ε if*

$$d_{\mathrm{GW},p}\left({\hat{S}}_{n},M_{\theta }\right)\leq \varepsilon .$$

*A derived kernel $k_{\theta }$ preserves empirical Solomonoff structure if its underlying geometry or kernel-induced metric satisfies the same condition.*

This test is stronger than spectral decay alone; it requires the pairwise algorithmic relationships among the data to be reproducible by the model geometry.

### 20.2. Certification Protocol

Four requirements make the protocol falsifiable rather than self-certifying (a relational-preservation check on the same data and compressor is selection-biased, and does not ensure passage of an independently fixed tolerance): *(a) held-out relations*—the preservation inequality must be evaluated on relations not used for fitting or selection (the inductive held-out protocol, E3); *(b) disjoint reference*—the reference dissimilarity must come from a compressor (or ensemble member) not used in construction; *(c) null calibration*—the tolerance ε must be calibrated against the structureless-data negative control (E2), since a pass threshold that structureless data also pass certifies nothing; and *(d) explicit pass/fail*—each item below must state its threshold before the data are seen.

A candidate Solomonoff-type model should be evaluated by the following tests:

1. **Semimeasure legitimacy:** Weights should derive from prefix-free or approximately prefix-free code lengths.
2. **Aggregation:** The model should aggregate mass over multiple explanations, not only over the shortest description.
3. **Algorithmic information capture:** Pairs with high algorithmic mutual information should be close even when ordinary statistical correlation is low.
4. **Spectral calibration:** Induced operator spectra should match $2^{-\ell_{i}}$ or a depth-truncated variant.
5. **Relational GW preservation:** The learned geometry should have small GW distance to the empirical Solomonoff mm-space.
6. **Cross-compressor stability:** The selected geometry should be stable across reasonable compressors or complexity surrogates.

The fifth item is the new criterion contributed by this paper.

## 21. Relation to Existing BAITML Components

### 21.1. Relation to MDL Kernel Learning

MDL kernel learning [1,97,98], in the tradition of MDL model selection [99,100,101,102,103], says to choose the kernel that compresses the data. Solomonoff–Gromov model selection says to choose the geometry with a relational structure that explains the algorithmic distances of the data via the shortest description. Thus, the MDL object shifts from a kernel to an mm-space.

### 21.2. Relation to KC-Kernels and D2KE

KC-kernels start from algorithmic similarities or distances. D2KE converts non-PSD distances into PSD kernels. The present paper inserts a geometry-learning layer before D2KE:

$${\hat{d}}_{\mathrm{Sol}}⟶{\hat{S}}_{n}⟶\hat{M}⟶k_{\hat{M}}.$$

Thus D2KE remains essential, but is applied to a selected explanatory geometry rather than directly to the raw empirical distance matrix.

### 21.3. Relation to SFM and SGP

The Solomonoff feature mixture constructs kernels from weighted features or explanations. In the geometry-first view, those features live on a selected mm-space. A Solomonoff Gaussian process is then a GP for which the covariance is induced by the learned algorithmic geometry. This makes explicit that an SGP is a second-order probabilistic surrogate, not an exact replacement for the discrete Solomonoff prior. For Gaussian regression, Proposition 10 closes the theory-to-prediction chain: the SGP posterior mean is exactly the KRR solution in the induced SGHS and has one kernel section per observation. The covariance weights $2^{-\ell_{r}}$ therefore survive computationally as the inverse spectral penalties $2^{\ell_{r}}$, subject to the explicit computability qualification of Remark 8.

### 21.4. Relation to the Countability–Gaussianity Mismatch

A limitation of Gaussianizing Solomonoff induction is that a non-degenerate Gaussian measure assigns probability zero to any fixed countable set of computable functions. The present paper avoids overstating the relationship. The SGP is not claimed to equal Solomonoff induction as a measure on hypotheses; it is a covariance-level and geometry-level surrogate, and the Solomonoff–Gromov criterion measures how well this surrogate preserves algorithmic relational geometry, not whether it reproduces the exact universal semimeasure.

### 21.5. Relation to Learning Theory

Learning rates depend on the geometry and spectrum induced by the selected model. If GW selection chooses a geometry for which the spectrum is Solomonoff-calibrated, then the resulting learning theory can be interpreted as learning under algorithmic compressibility. If it chooses a polynomial, plateau, or super-smooth geometry, then the mismatch can be diagnosed through the spectral and GW criteria described above.

### 21.6. Mutual Support with Parts VII–XII

The recovery results above make the link to Parts III–VI explicit [3,4,5,6]. The later parts are Part VII (kernel discrepancies and the bidirectional bridge) [104], Part VIII (kernel flows as random dynamical systems, with algorithmic randomness [105]) [106], Part IX (Solomonoff–Barron spaces) [107], Part X (hardness and tractability, building on [108]) [109], Part XI (Rademacher complexity through Kolmogorov complexity) [110], and Part XII (operator-adapted multiresolution, building on gamblets [111]) [112]. The programme survey is in [113]. The later parts of the programme fit the same tower but at different levels: they do not compete with Solomonoff–Gromov selection, but rather supply the diagnostics, dynamics, approximation spaces, hardness limits, capacity bounds, and multiresolution bases needed after an explanatory geometry has been selected. Table 14 summarizes these relationships.

This provides a two-way reading. The selected algorithmic mm-geometry supplies an input to the later diagnostics and learning constructions, while Parts VII–XII provide the statistical tests, dynamics, approximation spaces, hardness limits, capacity bounds, and multiresolution numerics needed after that selection step.

## 22. Examples and Experiments

We pair each quantitative example with the experiment that tests it. Every numerical value reported in this section comes from the numerical-validation programme reported in Section 14. All empirical numbers should be read as finite sanity checks unless accompanied by dispersion over seeds and the full protocol.

### 22.1. Worked Examples and Status

**Strings generated by grammars.** Strings from a small grammar; a Euclidean embedding of character counts may miss the grammar, while an NCD-based empirical Solomonoff space places strings with shared rules close. *Result (latent-structure recovery, E1; Section 14):* On data from K=3 low-complexity cores, the computed relational GW values in these runs decrease with *k* and does *not* identify the truth; the BIC/MDL-penalized objective with a cross-validated λ recovers K=3 on four of five exploratory data seeds and fails on one. The larger 20-plus-40 protocol separately measures transfer after supervised synthetic calibration.

**Program outputs.** $x_{i}=U\left(p_{i}\right)$ for short programs; the ideal geometry is the prefix tree of the $p_{i}$. This is the latent-structure recovery setting (E1) instantiated with program-defined cores; the GW coupling aligns outputs with program structure and D2KE yields a PSD kernel on program-output similarity.

**Algorithmically related pairs and dependence testing.** For y=g(x) with a simple computable transformation, algorithmic similarity offers a representation-free description even when the objects do not share a convenient vector space. This does *not* imply that characteristic-kernel tests fail: a deterministic relation is detectable by a properly calibrated characteristic-kernel independence test. Any comparison of NCD-based and conventional HSIC must use the same permutation calibration, sample size, and power criterion. Such a powered comparison is not part of the present numerical validation and remains future work.

**Physical fields with hidden low-complexity laws.** For PDE-generated data, the best candidate geometry may be operator-adapted/multiresolution rather than a prefix tree; GW selection would compare a manifold, a graph, and an operator-adapted hierarchy against the empirical Solomonoff space. This case is a motivating direction, not an executed result; the operator-adapted instantiation is future work (Section 23).

### 22.2. Executed Experimental Programme

The following summarizes the completed experiments.

**Prefix-tree/latent-structure recovery and its failure mode.** The latent-structure and inductive held-out studies (E1 and E3; Section 14) show that the BIC/MDL-penalized objective with cross-validated λ recovers the true number of latent components on 4/5 exploratory seeds; the relational term alone never does, and one seed admits no recovering λ. In the separate frozen confirmation protocol, 34/40 structured corpora are recovered after a ground truth-assisted calibration stage.

**Spectral calibration.** The synthetic spectral-calibration study (E4; Section 14) finds that the recovered kernel’s spectrum equals $2^{-\ell_{i}}$ exactly in the orthogonal control and degrades gracefully under coherence; the weights are inserted by hand on synthetic features, so this validates the mechanism of Theorem 2, not a recovery from compression data.

**Family-relative cross-compressor stability.** The cross-compressor study (E7; Section 14) finds that the top candidate is stable across zlib, bz2, and lzma, but distance-level ensemble instability is large (0.28), the planted-ambiguity detector caught 0/2 planted items, and, because all three compressors are finite-window statistical coders, the agreement certifies family-relative robustness only, not compressor-independent algorithmic structure.

**Task performance and its limitations.** The held-out prediction and real-data marked-selection studies (E10–E11; Section 14) provide the quantitative task-performance evidence. On periodic-versus-noise data, the selected geometry is competitive with raw compression baselines. On the cellular automaton task, unmarked relational selection collapses to chance-level balanced accuracy. On real SMS data, even fused marking does not rescue the inadequate block family; the validation safeguard recovers only by retaining the raw geometry, and the character 3–5-g SVM remains strongest. The benefit of the geometry selection layer over a raw compression kernel is not established on any tested task.

**Negative controls.** The structureless-data, held-out-prediction, and real-data marked-selection studies (E2, E10, and E11; Section 14) show that the frozen latent-structure protocol selects the trivial geometry on 39/40 fresh noise corpora; unmarked cellular-automaton selection reaches chance-level balanced accuracy after one-block collapse; and, on the natural-language task, pure marked block selection also collapses, while the conventional character baseline wins. These controls constrain the interpretation without establishing broad external validity.

## 23. Discussion, Limitations, and Open Problems

### 23.1. Computability

The true Solomonoff distance is incomputable. The framework is therefore a surrogate framework. Every practical result depends on the chosen compressor, program dictionary, or finite approximation.

### 23.2. GW Complexity

Exact GW optimization is computationally hard in general. Practical use requires entropic approximations, low-rank couplings, slicing, landmarks, or restrictions to special geometry classes such as trees or ultrametrics.

### 23.3. Metric Repair

Compression distances may violate metric axioms. The theory assumes metric inputs; practical algorithms need robust repair or dissimilarity-GW variants. If a raw dissimilarity *D* is repaired to a metric $D^{\mathrm{rep}}$, then the error decomposition should include a repair distortion term such as

$$\delta_{\mathrm{repair}}={∥D-D^{\mathrm{rep}}∥}_{\infty }$$

or the corresponding distortion in the chosen GW cost. One may absorb this into the compressor/sampling term only if the repair procedure and its distortion are reported; otherwise, it is a separate approximation layer. The following bound shows that this layer is controlled rather than uncontrolled.

**Proposition 20** (Metric-repair stability)**.**  *Let D be a symmetric non-negative dissimilarity on $X_{n}$ with zero diagonal, and let $D^{\mathrm{rep}}$ be any repair with $∥D-D^{\mathrm{rep}}{∥}_{\infty }\leq \delta_{\mathrm{repair}}$. Write $\tilde{S}=\left(X_{n},D,{\hat{\mu }}_{n}\right)$ and $S^{\mathrm{rep}}=\left(X_{n},D^{\mathrm{rep}},{\hat{\mu }}_{n}\right)$ for the two (dissimilarity-) mm-spaces on the same support and mass. Here, $d_{\mathrm{GW},p}$ denotes the finite GW functional evaluated on the dissimilarity matrices, and metricity of D is not used in the perturbation argument. Then, for every candidate mm-space M,*

$$\left|d_{\mathrm{GW},p}\left(\tilde{S},M\right)-d_{\mathrm{GW},p}\left(S^{\mathrm{rep}},M\right)\right|\leq 2^{-1/p}\;\delta_{\mathrm{repair}}.$$

*This is the bound for the unpowered GW distance. If the two distances in the absolute value are at most R, then the powered objective used in Equation (5) obeys*

$$\left|d_{\mathrm{GW},p}^{p}\left(\tilde{S},M\right)-d_{\mathrm{GW},p}^{p}\left(S^{\mathrm{rep}},M\right)\right|\leq pR^{p-1}2^{-1/p}\;\delta_{\mathrm{repair}}.$$

*Thus, metric repair is controlled by the sup-norm repair distortion, with distinct constants and units for the unpowered distance and the powered Solomonoff–Gromov objective.*

**Proof.**  The two spaces share support and mass, so any coupling admissible for one is admissible for the other, and the two GW integrands differ only through the source cost, uniformly by at most $\delta_{\mathrm{repair}}$. This is Proposition 6 with $\varepsilon =\delta_{\mathrm{repair}}$; the $\frac{1}{2}$ normalization of Definition 2 supplies the $2^{-1/p}$ factor, and exchanging the two spaces gives the absolute value. Only perturbation of the cost matrix is used, so the bound holds for the dissimilarity-GW value even when *D* is not a metric. The powered bound follows from $|a^{p}-b^{p}|\leq pR^{p-1}\left|a-b\right|$. □

### 23.4. Infinite-Dimensional SGP GW

A full theory of GW between infinite-dimensional Gaussian process measures requires care. Finite-dimensional Gaussian and covariance-operator proxies are tractable, but the infinite-dimensional non-entropic GW problem remains delicate.

### 23.5. Universal Dominance

Solomonoff’s universal dominance is a property of the discrete universal semimeasure. A Gaussian or kernelized surrogate does not automatically inherit it. The correct claim is weaker: the surrogate preserves selected relational, spectral, and algorithmic-information features of Solomonoff induction. The mixture of Section 12 *does* inherit a dominance/regret bound (Proposition 15), but at the level of the candidate geometry class, with the computable code length in place of *K*, not the unrestricted universal dominance.

### 23.6. Space-Mixture Truncation

The space mixture $P_{\mathrm{SpaceMix}}$ is only computable after truncating the mm-model collection A to a finite subset with computable weights and component laws, or supplying an effective tail bound for an infinite sum. Evaluating each weight requires a fixed GW distortion, evidence, and a code length. Thus, practical use requires finite dictionaries, beam search, or another declared enumeration scheme. The unnormalized log-evidence price of truncation is the omitted-mass term in Proposition 16; normalized sequential log-loss includes the initial-mass correction given there; designing enumerations for which that mass is estimable or non-vacuously bounded remains open.

### 23.7. Scope of the Proved Results

We restate here, in one place, the limitations of the results. *(i) Incomputability.* The deficiency Δ and regret $R_{T}$ (Section 13) reference the uncomputable *M*; they are the correct targets, but are not evaluable against *M* itself, only against computable surrogates. The experiments check finite consequences and numerical selection behavior, not theorems in full generality or distance to *M*. *(ii) Spectrum hypothesis*. Theorem 2’s near-orthogonality condition (H2_δ_) is restrictive across length classes with large weight dynamic range. The exact product-spectrum case assumes orthogonality; the global two-sided and per-index bands are finite-dictionary statements; and part 2 also states a local localization for countably infinite dictionaries. The relative quadratic-form comparison in the proof gives an additional trace-class extension when δ<1. The stated conservative per-index band uses normalized features and δκ<1; the relative form bound alternatively gives this calibration for δ<1. Neither bound fixes eigenvectors without spectral gaps. The favorable synthetic spectral-calibration behavior (E4) is empirical, not a consequence of a tight worst-case band. *(iii) Compressor circularity.* The empirical reference ${\hat{S}}_{n}$ is built from a compressor, so the selection compares against a compressor surrogate; the cross-compressor study (E7, Section 14) is the strongest available hardening; it certifies family-relative robustness only, and its planted-ambiguity control failed, both of which are reported as such. *(iv) GW vs. prediction.* $d_{\mathrm{GW}}$ is isometry-invariant, and hence certifies relational covariance geometry rather than the predictor; predictive validity additionally requires task alignment and an adequate candidate class, as the cellular-automaton and SMS studies (E10–E11) show, and the loss of information is made precise by Proposition 17. The SMS post-hoc oracle audit (E11) is limited to the fixed average-linkage partitions, D2KE kernel, and KRR learner; it is not an impossibility theorem for all supervised quotients. *(v) No real-data task win.* We provide synthetic recovery with an honest failure rate (latent-structure and inductive studies, E1 and E3), controls (structureless data, compressor variation, and held-out prediction; E2, E7, and E10), and a nested real-data marked-selection study (E11). Pure fused marking still collapses on SMS; the safe selector matches raw D2KE only by retaining the source geometry, and character features remain strongest. A larger pre-registered and adversarially fair real-data evaluation and an estimable surrogate for $R_{T}$ remain open work; neither is claimed here. *(vi) Status of the position.* Category-native approximation is defended as an organizing framework with explicit commitments and a maturity map. Only the metric–measure branch is developed to the theorem level represented here; this paper does not claim a uniform cross-category oracle theorem.

### 23.8. Open Problems

1. Prove sharp finite-sample rates for empirical Solomonoff–Gromov convergence under compressor distortion, including explicit metric-repair distortion terms.
2. Extend the empirical-GW consistency theorem from i.i.d. sampling to ergodic/trajectory sampling (Birkhoff-type), the case relevant to the dynamical-systems interface of Parts VIII and X; a worked symbolic-dynamics example (logistic-map trajectory windows across the period-doubling cascade, with the tree candidate expected in the periodic regime and the trivial geometry in the chaotic regime) is the natural first target.
3. Develop uniform predictive generalization bounds for learners induced by continuous geometry classes, beyond the uniform geometric comparison in Remark 27.
4. Characterize when GW minimization over ultrametric candidates recovers the true prefix tree.
5. Develop differentiable GW objectives for parametric program feature families.
6. Establish infinite-dimensional IGW theory for trace-class covariance operators relevant to SGPs.
7. Prove lower bounds showing that no computable geometry can achieve zero GW distance to the full Solomonoff mm-space.
8. Determine when spectral Solomonoff calibration plus relational GW preservation implies useful learning rate improvements.
9. Design computable enumeration or search schemes for $P_{\mathrm{SpaceMix}}$ in which the omitted mixture mass $r_{N}\left(x_{1:n}\right)$ is estimable or bounded non-vacuously.
10. Design benchmark datasets that distinguish Solomonoff-type priors from generic complexity-penalizing priors.
11. Sharpen the finite entropy bound of Proposition 19 into geometry-dependent entropic bias rates and quantify low-rank or sliced-GW approximation errors while separating statistical error, regularization bias, and numerical solver error.
12. Replace the declared *k*-block BIC score by an explicit code for the fitted partition and quantized block parameters, and determine how the penalty-domination boundary changes under that honest code.
13. Characterize quotient adequacy by giving conditions under which a marked candidate family preserves enough task information for predictive competitiveness, and extend Proposition 2 beyond the *k*-block menu without mistaking a sufficient collapse certificate for a converse.
14. Develop one non-metric–measure branch to theorem level (for example, the topological, Banach/Barron, graph, or operator branch) with category-native stability and oracle guarantees.

The scope table in Table 1, assumption ladder in Table 13, and the predictive-collapse and real-data marked-selection analyses in Table 8, Table 9 and Table 10 collect the claim boundaries that matter for interpreting the technical results.

## 24. Conclusions

This paper argues for category-native Solomonoff approximation as the layer between an incomputable universal predictor and practical machine learning representations. The thesis is that choosing a computable shadow means choosing which invariants to preserve; therefore, metric–measure, Hilbert/Banach, topological, graph, tree, operator, and predictive representations should be compared and used through their native structures rather than being forced into a single universal proxy. Equation (1) makes that position operational by coupling native distortion, description length, and when needed, predictive loss.

The metric–measure branch demonstrates what it means to develop one branch of this programme. Compression-derived distances and empirical or Occam masses define an empirical mm-space; candidates are compared by GW distortion and penalized by a declared MDL score, and the selected geometry is kernelized by D2KE. For finite or countable classes, the manuscript proves well-posedness, perturbation and transfer results, validation bounds, recovery of the earlier Kolmogorov/Solomonoff kernels, and a redundancy bound for coded mm-model mixtures. The broader oracle inequality is conditional on marked selection, code-weighted concentration, and learner stability. These assumptions are design obligations, not consequences of the position itself.

The experiments make the position more precise. The structural-recovery, negative-control, spectral, stability, metric-repair, compressor, Occam-mass, and marked-coupling studies (E1–E9) check isolated mechanisms, mostly on synthetic data. The held-out cellular-automaton study (E10) shows that unmarked relational selection can collapse to a one-block geometry and reach chance-level balanced accuracy; its penalty-domination certificate is empirically sound, but visibly one-sided. The real-data SMS study (E11) shows that fused marks alone do not repair an inadequate quotient family: even a post hoc oracle over all four implemented block kernels remains at chance, the safe selector recovers only by retaining the raw geometry, and a conventional character baseline remains strongest. The lesson is not that representation should cease to be category-native but that the native object for a supervised problem must be task-aligned *and* that the candidate family must contain an adequate representation. Geometry is a structural prior, not a substitute for task alignment or family design.

The category-native claim is consequently both ambitious and bounded. It is ambitious because it proposes a common research architecture and treats kernels as one projection among several rather than the default language for every approximation, and is bounded because Table 4 distinguishes developed, partial, and constructional branches while not claiming any cross-category oracle theorem. This paper’s intended contribution is the vision together with one technically mature realization and an explicit account of what would confirm, refine, or falsify the remaining branches.

## Figures and Tables

**Table 1:** Status and scope of the manuscript’s principal claims.

Claim	Status	Scope or Additional Requirement
Category-native decomposition of comparison, representation, and induction	position/framework claim	defended as an organizing principle and research programme; not asserted as a single cross-category theorem
GW + MDL selection and finite perturbation bounds	proved	finite or compact mm-space candidate classes under the stated metric and solver assumptions
Held-out oracle inequality	proved finite/countable core	coded candidates fitted independently of the validation sample
GW-to-kernel and risk transfer	proved in finite form; conditional in the broad form	D2KE/operator stability and the displayed finite-rank or trace assumptions
Countable geometry oracle inequality	conditional theorem	marked/predictive selection, code-weighted concentration, and fixed-geometry learner control
Topological, Banach/Barron, graph, tree, and broad operator branches	constructional programme	proposed native objects and comparisons are part of the position; branch-specific stability and oracle results remain open

**Table 2:** Acronyms used in the manuscript.

Acronym	Meaning
AMI	algorithmic mutual information
AIT	algorithmic information theory
BIC	Bayesian information criterion
CND	conditionally negative definite
CKC	conditional Kolmogorov complexity kernel or conditional complexity surrogate
D2KE	distance to kernel and embedding
ESS	effective sample size
FGW	Fused Gromov–Wasserstein
GH	Gromov–Hausdorff
GW	Gromov–Wasserstein
HSIC	Hilbert–Schmidt independence criterion
KL	Kullback–Leibler
KC	Kolmogorov complexity-based
KRR	kernel ridge regression
MDL	minimum description length
MMD	maximum mean discrepancy
mm-space	metric–measure space
NCD	normalized compression distance
NID	normalized information distance
RKBS	reproducing kernel Banach space
RKHS	reproducing kernel Hilbert space
SFM/SKCO	Solomonoff Feature Mixture/Solomonoff Kernel Covariance Operator
SGP/SGHS	Solomonoff Gaussian Process/Solomonoff Gaussian Hilbert Space
SMS	Short Message Service
TDA	topological data analysis
TV	total variation
UCI	University of California, Irvine

**Table 3:** Notation distinguishing ideal, empirical, and selected objects.

Symbol	Meaning
*M*, *K*	ideal universal semimeasure and prefix Kolmogorov complexity; both are incomputable
${\hat{S}}_{n,C}$	empirical mm-space built from *n* observations and compressor *C*
$M_{\theta }$, $\hat{\theta }$	candidate mm-space and selected index
$d_{\mathrm{GW},p}$	order-*p* GW distance with the 1/2 normalization of Definition 2
Code(θ), λ	candidate code length or declared MDL score together with its penalty weight
$J_{n}$, $J_{n}^{\mathrm{mark}}$	unmarked and marked selection objectives
$\delta_{\mathrm{comp}},\delta_{\mathrm{samp}},\delta_{\mathrm{opt}}$	compressor, sampling, and optimization errors

**Table 4:** Maturity map of the category-native programme. The proposed comparisons are hypotheses to be developed and compared, not assertions that every row already has an end-to-end oracle theorem.

Target Category	Native Comparison Candidate	Inductive or Representational Object	Maturity in This Paper
Metric–measure	GW or marked/fused GW	D2KE kernel, induced operator, or coded mm-model mixture	developed realization with finite/countable theorems and conditional learning results
Hilbert/RKHS	kernel, operator, or subspace discrepancy	kernel learner, Gaussian process, covariance operator	recovered from the selected mm-geometry and connected to Parts I–VI
Banach/Barron	norm, Lipschitz, Banach–Mazur, or variation discrepancy	variation-norm regularizer and feature measure	constructional proposal; approximation-to-risk theorem remains open
Topology	bottleneck or interleaving distance on coded filtrations	persistence summary, kernel, or classifier	constructional proposal; Solomonoff-side filtration and predictive stability remain open
Graphs	graph-edit, diffusion, or spectral comparison	Occam-weighted Laplacian, diffusion, or graph kernel	constructional proposal; sampling and marked-selection theory remain open
Trees/ultrametrics	tree alignment or ultrametric distortion	prefix/context-tree predictor	partially developed through the ultrametric mm-space branch
Operators	Hilbert–Schmidt, trace, resolvent, or spectral comparison	spectral regularizer, covariance, or evolution operator	partially developed after kernelization; eigenfunction-sensitive control remains open
Predictive laws	held-out loss, log loss, deficiency, or regret	direct computable mixture predictor	closest to Solomonoff’s predictive semantics; unrestricted universality remains incomputable

**Table 5:** Triage of restricted metric–measure approximation problems.

Source Metric	Target Class	Status
Any mm-space	all mm-spaces	Trivial: choose the source itself, so GW can be zero.
Prefix ultrametric $d_{\mathrm{tree}}$	tree or ultrametric spaces	Mostly structural: useful for computable approximation and recovery, not for discovering that the object is tree-like.
Prefix ultrametric $d_{\mathrm{tree}}$	Hilbert spaces	Often exact in finite/proper settings because ultrametrics admit isometric Hilbert embeddings.
Predictive Hellinger distance	Hilbert spaces	Exact at unrestricted rank by the square-root embedding of probability laws. Nontrivial only with rank, code, or computability constraints.
Algorithmic distance, NID/NCD, CKC, AMI	Hilbert/RKHS kernels	Nontrivial when the metric has positive mass-weighted non-Hilbertian or non-PSD defect; D2KE/SFM kernelization is then needed.
Posterior explanation geometry	Gaussian/SGP	Nontrivial and lossy: Gaussianization keeps covariance structure but discards atoms and higher moments.
Topological candidate	GW	GW is defined only after choosing a compatible metric and measure; otherwise use persistence or another topological invariant as a calibration layer.

**Table 6:** Native comparison functionals and the structures they are intended to preserve.

Target Class	Preserved Structure	Native Comparison
Topological spaces	components, holes, branches, shape	persistent homology, bottleneck/interleaving distance
Metric spaces	distances without mass	Gromov–Hausdorff, Lipschitz distortion
Metric–measure spaces	distances weighted by mass	Gromov–Wasserstein
Hilbert spaces	Euclidean/Hilbert geometry	Hilbert distortion, CND defect, Hilbert-restricted GW
Banach spaces	normed linear geometry	Banach–Mazur distance, Banach distortion, RKBS diagnostics
RKHS/kernel models	kernel-induced geometry and embeddings	kernel alignment, MMD, operator norms
Gaussian/SGP models	covariance geometry and spectra	Gaussian GW/OT, Hilbert–Schmidt, trace, spectral distances
Predictive models	conditional laws and sequential forecasts	KL, Hellinger, total variation, log-loss regret

**Table 7:** Category-native induction dictionary: the selected object, its native induction rule, and its Solomonoff-calibrated version.

Category	Object	Induction Rule	Solomonoff Version
Metric	(Z,d)	nearest-neighbour, distance kernel, Nadaraya–Watson	algorithmic *d*, Occam weights $w\propto 2^{-K}$
Metric–measure	(Z,d,μ)	D2KE/SFM followed by KRR or GP	$\mu \propto 2^{-\ell }$
Hilbert	Z⊂H	linear/RKHS regression, Gaussian expansion	$\lambda_{i}≍2^{-\ell_{i}}$
Banach	${B,∥\cdot ∥}_{B}$	norm, RKBS, sparse/variation regularization	$\sum_{\omega }2^{\ell (\omega )}\left|a_{\omega }\right|$ penalty
Topology	(Z,τ)	persistent-feature kernel prediction	$2^{-\mathrm{Code}}$-weighted topological features
Graph	(G,w,μ)	diffusion, random walk, Laplacian learning	Occam-weighted graph diffusion
Tree	$\left(T,d_{\mathrm{tree}},\mu_{T}\right)$	context-tree, prefix mixture, prefix kernel	$\sum_{u⪯x,y}2^{-|u|}$
Operator	*T*	spectral KRR or GP	$\lambda_{i}≍2^{-\ell_{i}}$
Predictive	$P(\cdot |x_{<t})$	direct sequential prediction	$\sum_{\nu }2^{-K(\nu )}\nu$-style mixture
SGP	$\left(H,\gamma_{C}\right)$	GP posterior prediction	covariance spectrum $2^{-\ell_{i}}$

**Table 8:** Held-out prediction results (E10) and the split-aware interpretation of collapse. The 15-seed non-stratified rows use transductive input geometry with held-out labels and report means with 1.96 times the sample standard deviation divided by $\sqrt{15}$. Cellular-automaton replicates share generated strings because their initial-state seed ranges overlap, so these bars are descriptive and do not have established 95% coverage. The three 20-split stratified rows are means with descriptive per-split standard deviations over a fixed balanced dataset. They are grouped separately because the protocols and dispersion summaries are not inferentially identical.

Task	Selection or Baseline	Raw Accuracy	Balanced Accuracy	Interpretation
Periodic vs. noise	unmarked GW + MDL geometry	0.992±0.011	0.991±0.012	competitive, but no gain over raw NCD
Cellular automata (15 non-strat.)	unmarked GW + MDL geometry	0.154±0.033	0.247±0.039	k=1; chance-level balanced performance
Cellular automata (15 non-strat.)	training-majority null	0.144±0.025	0.233±0.033	explains the misleadingly low raw score
Cellular automata (15 non-strat.)	raw D2KE–NCD	0.559±0.094	0.652±0.075	local compression geometry retains signal
Cellular automata (15 non-strat.)	NCD-3NN	0.785±0.083	0.810±0.069	strongest method in this protocol
Cellular automata (20 strat.)	nested task-aware D2KE	0.785±0.139	0.785±0.139	usually retains raw geometry
Cellular automata (20 strat.)	raw D2KE–NCD	0.838±0.067	0.838±0.067	same splits and KRR learner as nested selection
Cellular automata (20 strat.)	NCD-1NN	0.755±0.043	0.755±0.043	same stratified splits; different learner

**Table 9:** Empirical audit of the sufficient penalty-domination certificate at $\lambda =2\times 10^{-4}$. “Fires” means $\lambda >\lambda_{★}$; soundness requires only that every firing imply k=1. The long-ECA row changes object length, not sample count. CV_off_ is the coefficient of variation of training off-diagonal NCD values.

Corpus	$n_{\mathrm{tr}}$	Mean CV_off_	$\lambda_{★}$ Range	Fires/Collapse
Periodic vs. noise	24	0.197	$\left[1.23,1.95\right]\times 10^{-3}$	0/15/0/15
Three-family	14	0.151	$\left[0.784,1.63\right]\times 10^{-3}$	0/15/0/15
ECA four rules	19	0.064	$\left[1.37,2.87\right]\times 10^{-4}$	4/15/15/15
ECA four rules, 48×48	19	0.036	$\left[0.698,1.02\right]\times 10^{-4}$	15/15/15/15

**Table 10:** Real-data marked selection on the balanced UCI SMS subset (E11). Entries are mean balanced accuracy ± descriptive standard deviation across ten repeated stratified splits. Accuracy is identical here because every test split is balanced. The post hoc oracle uses test labels only to take the maximum score over the four already fitted block-kernel pipelines; it is a diagnostic, not a usable selector.

Method	Balanced Accuracy	Interpretation
Unmarked GW + MDL block selection	0.500±0.000	selects k=1 in all 10 splits
Fused/marked GW + MDL block selection	0.500±0.000	selects k=1 in 9/10 splits and k=2 once
Post-hoc oracle over the four fixed block kernels	0.500±0.000	test-label diagnostic; every k∈{1,2,3,4} scores chance
Marked selection with raw-geometry safeguard	0.910±0.048	inner validation retains raw geometry in all 10 splits
Raw D2KE–NCD	0.910±0.048	no gain from quotient selection
NCD-1NN	0.895±0.030	strong local compression baseline
Character 3–5-g SVM	0.947±0.022	strongest tested real-data method

**Table 11:** Numerical mechanism checks for Parts III–VI.

Part	Mechanism Check	Numerical Result
III	D2KE/KC-kernel PSD repair	The raw distance matrix is indefinite as a Gram matrix (minimum eigenvalue −2.46), whereas D2KE is PSD (minimum eigenvalue $1.8\times 10^{-2}$) at computed selected-geometry GW 0.0021. Raw-Gram indefiniteness is not a test of Hilbert embeddability; the appropriate test is PSD of $-\frac{1}{2}JD^{\circ 2}J$, with $J=I-11^{⊤}/n$.
IV	SFM/Occam spectral calibration	Ratio $\lambda_{i}/2^{-\ell_{i}}\in \left[1.00,1.00\right]$ at exact orthogonality, degrading to [0.75,1.02] at coherence 0.1 (spectral-calibration study, E4); the exact identity holds only in the orthogonal control.
V	Effective-dimension ordering	At ridge $10^{-2}$, the decaying D2KE spectrum has effective dimension 3.52 against 4.36 for a spectrum-flattened kernel of equal trace: Occam-type decay reduces capacity, a finite equal-trace comparison that does not establish a population learning rate.
VI	Pullback spectrum preservation	Pulling the selected candidate’s D2KE kernel back through the optimal coupling preserves the top-5 spectrum to relative deviation $<10^{-3}$, the Part-VI preservation mechanism.

**Table 12:** Recovery of the kernel and learning-theory objects from Parts III–VI after metric–measure selection.

Part	Recovered Object	How It Appears Here
III	D2KE and spectral regimes	Kernelize the selected algorithmic mm-space and study the spectrum of $T_{D2\mathrm{KE},\hat{M}}$.
IV	SFM, SKCO, SGP, SGHS	Keep the same features but replace uniform landmark mass by Occam mass $2^{-\ell }$.
V	learning rates and spectral collapse	Apply fixed-kernel learning theory to the operator induced by the selected mm-space.
VI	deep KC and pullback kernels	Let the candidate geometry itself be a learned pullback $\Phi_{\theta }^{*}d_{Z}$.

**Table 13:** Assumption ladder connecting the metric–measure selector to the conditional learning-oracle statement.

Step	Additional Assumption	What It Buys, and How It Can Fail
GW + MDL selection	finite or compact mm candidate class; declared code or complexity score	existence of a selected geometry; says nothing yet about predictive risk
Finite validation bound	Kraft-coded candidates fitted independently of validation data	uniform held-out comparison by a code-weighted union bound; fails under test-set reuse or an uncoded continuous search
Geometry-to-kernel transfer	bounded finite mm-spaces and transported D2KE stability	small GW controls the induced-kernel discrepancy; an arbitrary kernelization need not be Lipschitz
Fixed-geometry learner bound	source/effective-dimension conditions and a valid transport between the relevant $L^{2}$ spaces	a KRR bias–variance statement conditional on the geometry; these quantities must be estimated or bounded for the chosen family
Countable geometry oracle	marked or independent predictive selection, code-weighted concentration, and finite-rank or trace stability	comparison with the best included geometry; unmarked GW, training error, or operator norm alone is insufficient

**Table 14:** Relationship between Parts VII–XII and the metric–measure selection layer.

Part	Object	Role Relative to Metric–Measure Selection
VII	MMD, KSD, HSIC	These are distributional, Stein, and dependence tests *after* kernelization. GW selects the basis-free relational geometry; MMD/KSD/HSIC supply basis-sensitive diagnostics and marked/task losses on the induced RKHS or on the coupling.
VIII	Kernel Flows/RDS	Kernel-flow dynamics become optimization dynamics on the selected family G or on the induced kernels $T_{M}$. The SG objective supplies the geometric energy landscape; Part VIII supplies convergence and random-dynamical-system tools for moving through it.
IX	Solomonoff–Barron spaces	The selected mm-space supplies the explanation/feature measure. Barron-type variation norms then quantify approximation complexity after the universal posterior geometry is projected to a feature dictionary.
X	Hardness and tractability	The SG selection problem inherits the hardness of GW, kernel search, and operator learning. Part X explains which restrictions–trees, ultrametrics, low-rank kernels, entropic relaxations, finite dictionaries–turn the geometry programme into a tractable one.
XI	Entropy and Rademacher complexity	The effective dimension $N_{M}\left(\lambda \right)$ and spectral dimension $\beta_{M}$ in Section 17 are the operator side of the entropy/Rademacher bounds. Part XI controls the uniform finite-sample cost of selecting over G and over the induced function classes.
XII	Gamblets and multiresolution	Gamblets provide operator-adapted multiresolution bases once an operator or geometry has been chosen. The SG layer explains which hierarchy should be chosen; the gamblet layer gives the computational basis in which the selected geometry becomes fast and stable.

## Data Availability

The only external dataset used in this study is the SMS Spam Collection, publicly available from the UCI Machine Learning Repository under CC BY 4.0, DOI: https://doi.org/10.24432/C5CC84 [77]. All remaining empirical inputs are synthetic; the experimental designs are described in Section 14 and Section 22. No private dataset was used.
